# SpaBatch: Deep Learning‐Based Cross‐Slice Integration and 3D Spatial Domain Identification in Spatial Transcriptomics

**DOI:** 10.1002/advs.202509090

**Published:** 2025-09-15

**Authors:** Jinyun Niu, Donghai Fang, Jinyu Chen, Yi Xiong, Juan Liu, Wenwen Min

**Affiliations:** ^1^ School of Information Science and Engineering Yunnan University Kunming Yunnan 650500 China; ^2^ School of Mathematics, Statistics and Mechanics Beijing University of Technology Beijing 100124 China; ^3^ School of Life Sciences and Biotechnology Shanghai Jiao Tong University Shanghai 200240 China; ^4^ School of Artificial Intelligence School of Computer Science Wuhan University Wuhan Hubei 430072 China

**Keywords:** 3D spatial domain identification, batch effect correction, contrastive learning, graph neural networks, multi‐slice spatial transcriptomics, triplet learning

## Abstract

With the rapid accumulation of spatial transcriptomics (ST) data across diverse tissues, individuals, and technological platforms, there is an urgent need for a robust and reliable multi‐slice integration framework to enable 3D spatial domain identification. However, existing methods largely focus on 2D spatial domain identification within individual slices and fail to adequately account for inter‐slice spatial correlations and batch effect correction, thereby limiting the accuracy of cross‐slice 3D spatial domain identification. In this study, SpaBatch is presented, a novel framework for integrating and analyzing multi‐slice ST data, which effectively corrects batch effects and enables cross‐slice 3D spatial domain identification. To demonstrate the power of SpaBatch, SpaBatch is applied to eight real ST datasets, including human cortical slices from different individuals, mouse brain slices generated using two different techniques, mouse embryo slices, human embryonic heart slices, HER2+ breast cancer tissues and mouse hypothalamic slices profiled using the MERFISH platforms. Comprehensive validation demonstrates that SpaBatch consistently outperforms state‐of‐the‐art methods in 3D spatial domain identification while effectively correcting batch effects. Moreover, SpaBatch efficiently captures conserved tissue architectures and cancer‐associated substructures across slices, and leverages limited annotations to predict spatial domain in unannotated sections, highlighting its potential for tissue‐structure interpretation and developmental biology studies. All code and public datasets used in this study are available at: https://github.com/wenwenmin/SpaBatch.

## Graphical abstract

SpaBatch is an end‐to‐end multi‐slice spatial transcriptomics data integration framework. It simultaneously performs embedding learning, spatial feature denoising and reconstruction, batch effect correction, and spatial domain optimization, effectively correcting batch effects and achieving accurate 3D spatial domain identification. Validation across multiple datasets shows that SpaBatch outperforms existing methods and demonstrates broad applicability and robustness across spatial transcriptomics data from different species, platforms, and tissue types.

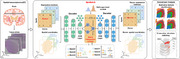

## 1 Introduction

Spatial transcriptomics (ST) is a rapidly advancing technology that enables the capture of gene expression within tissues while retaining spatial context. This offers critical insights into the spatial organization of cells and the architecture of tissue structures.^[^ ^1^ ^]^ In recent years, a variety of ST techniques with different spatial resolutions have been developed. For example, 10x Visium^[^ ^2^ ^]^ employs capture probes with a diameter of 55 μm to quantify transcript abundance at the spot level, where each spot generally encompasses multiple cells. Slide‐seq^[^ ^3^ ^]^ and its improved version Slide‐seqV2^[^ ^4^ ^]^ achieve higher resolution at approximately 10 μm per spot, approaching near single‐cell resolution. Stereo‐seq^[^ ^5^ ^]^ achieves a 500 nm (0.5 μm) spatial resolution, making a breakthrough in ST technology with its ultra‐high resolution and centimeter‐scale tissue coverage.

In ST data analysis, spatial domain identification is one of the core tasks. It aims to partition tissue sections into multiple functional regions, ensuring that spatial spots within the same domain exhibit similar gene expression patterns.^[^ ^6^ ^]^ In recent years, significant advances have been made in 2D spatial domain identification for individual slices. Methods incorporating spatial information by using graph neural networks^[^ ^7^, ^8^ ^]^ and deep learning approaches integrating ST data with histological images^[^ ^9^, ^10^, ^11^ ^]^ have notably improved the accuracy of spatial domain identification in single‐slice ST data analysis. However, the biological activities of organisms and tissues are inherently 3D.^[^ ^12^ ^]^ With the continuous accumulation of ST datasets generated under various conditions, technologies, and platforms, there is an urgent need to jointly analyze ST data from multiple tissue sections in order to uncover complex tissue architectures and decode biological patterns across individuals.^[^ ^13^ ^]^ Nevertheless, integrating ST data across multiple slices and 3D spatial domain identification pose substantial challenges, such as spatial misalignment and batch effects. Several methods have been proposed to address these issues, for example, GraphST^[^ ^14^ ^]^ performs multi‐slice coordinate alignment in the initial step and utilizes the PASTE algorithm^[^ ^15^ ^]^ to correct batch effects in the latent embeddings. However, the accuracy of this method largely depends on the precision of the spatial coordinate alignment. DeepST^[^ ^10^ ^]^ employs deep graph neural networks for representation learning and incorporates a domain‐adversarial network (DAN) to correct batch effects. However, its training procedure is time‐consuming and does not lead to more effective integration of different samples. STAligner^[^ ^16^ ^]^ performs representation learning on multi‐slice joint data using STAGATE^[^ ^8^ ^]^ and adjusts the latent embeddings based on the mutual nearest neighbor (MNN) approach. This method aims to minimize the distance between positive pairs while maximizing the distance between negative pairs. However, it may overlook non‐MNN pairs that originate from the same spatial domain and mistakenly include MNN pairs that belong to different domains. STG3Net^[^ ^17^ ^]^ utilizes deep graph neural networks for representation learning and performs pre‐clustering on the latent embeddings to define positive and negative pairs across different samples. It then applies triplet contrastive learning during training to progressively bring positive pairs closer and push negative pairs further apart, thereby correcting batch effects. However, the pre‐clustering process may result in incorrect cluster assignments, which can negatively affect subsequent training.

Besides, methods such as stlearn and STitch3D,^[^ ^9^, ^18^, ^19^, ^20^ ^]^ need to integrate ST data with additional data sources, such as scRNA‐seq profiles from the same tissue or histological images, limiting their widespread applicability. Existing methods often exhibit insufficient capabilities in correcting batch effect and suffer from instability in cross‐slice spatial domain identification. Moreover, most approaches are unable to output corrected gene expression data, which restricts their utility for downstream analyses.

**Figure 1:**
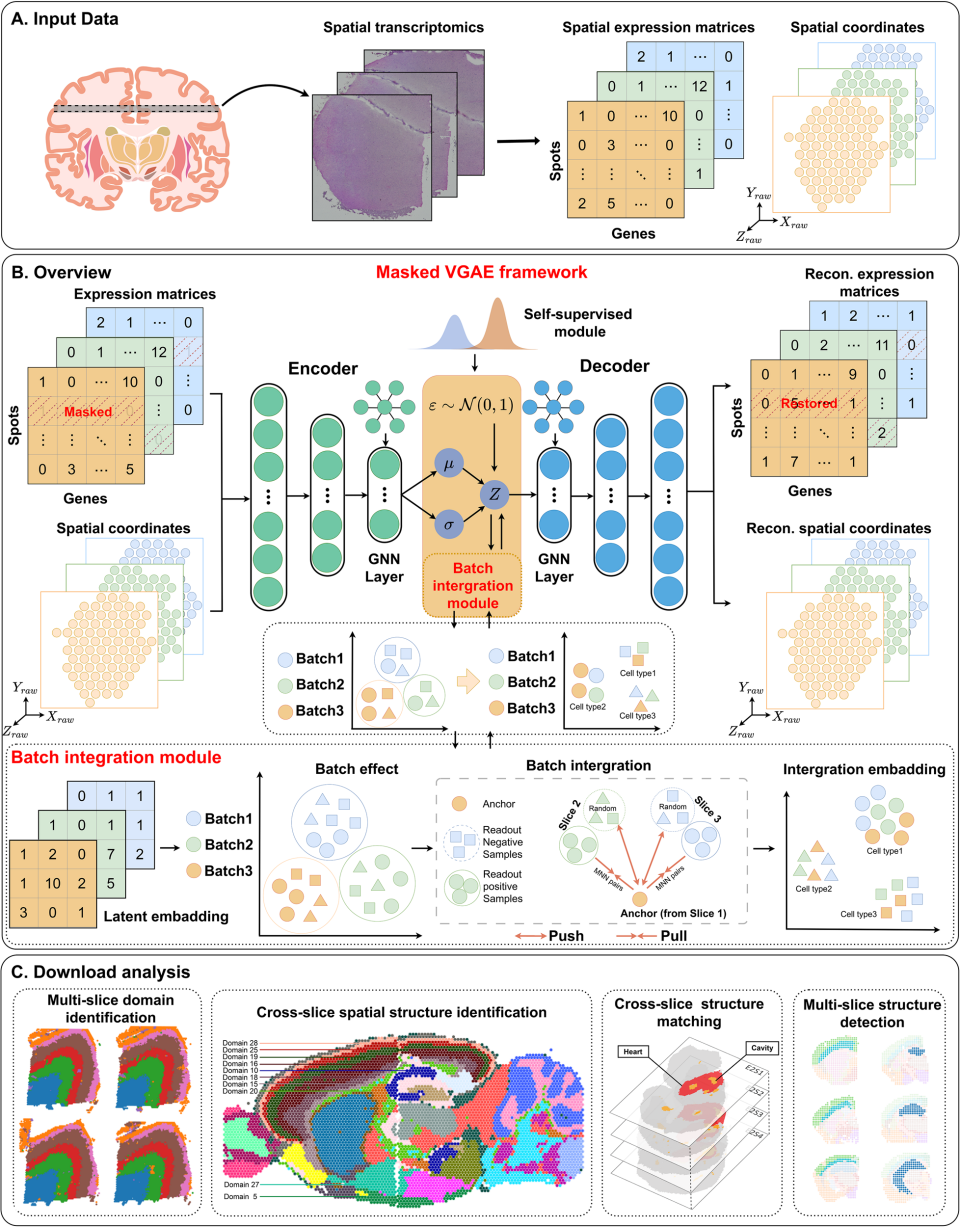
The architecture of SpaBatch. A) Input data and data preprocessing. B) Backbone network of SpaBatch. C) Downstream analysis. The brain slice illustration in panel A (Input Data, left) was adapted from Bioicons (https://bioicons.com) and is used under the Creative Commons Attribution 4.0 International License (CC BY 4.0).

To address these limitations, we propose a novel multi‐slice integrative analysis framework named SpaBatch (**Figure** **1**). Specifically, SpaBatch first applies a masking strategy to perturb the input gene expression profiles, thereby improving the model's robustness to missing values and technical noise commonly present in spatial transcriptomics data. During training, SpaBatch adopts a two‐stage strategy consisting of pretraining and fine‐tuning. In the pretraining stage, it employs a variational graph autoencoder (VGAE) to jointly model gene expression and spatial adjacency information, learning low‐dimensional latent embeddings that capture the underlying spatial transcriptomic landscape. In the fine‐tuning stage, a Deep Embedded Clustering (DEC) module is introduced to iteratively refine the embedding space and enhance spatial compactness within the identified domains. To further address inter‐slice variability and mitigate batch effects, SpaBatch incorporates a readout‐based triplet contrastive learning mechanism that aggregates multiple positive samples across slices while maximizing the distance between positive and negative pairs. The introduction of dual‐stage fine‐tuning enables the model to align biologically coherent structures across slices while preserving the discriminative spatial features necessary for accurate 3D spatial domain identification.

We applied SpaBatch to eight spatial transcriptomics (ST) datasets from diverse tissue types (human dorsolateral prefrontal cortex, mouse brain, human embryonic heart, HER2+ breast cancer), species, and platforms (10x Visium, ST, Stereo‐seq, MERFISH). The experimental results on the DLPFC dataset demonstrate that SpaBatch outperforms state‐of‐the‐art methods in both spatial domain identification (average ARI = 0.613 on sample 3 of the DLPFC dataset, while all other methods fall below 0.6) and batch effect correction (compared with methods such as STG3Net and DeepST, SpaBatch achieves the best‐balanced performance). In the mouse brain dataset, SpaBatch further leverages two distinct datasets derived from different ST platforms to comprehensively resolve complex spatial organization at both macro and micro levels–such as the trisynaptic hippocampal circuit–with validation through marker gene expression. When applied to a human embryonic heart dataset containing multiple slices across developmental stages (4.5–6.5 post‐conceptual weeks, PCW), SpaBatch effectively captures dynamic spatiotemporal changes during cardiac development. Interestingly, when integrating the HER2+ breast cancer dataset, SpaBatch adopts the semi‐supervised learning strategy that leverages the limited annotation from one annotated slice and identifies cancerous and non‐cancerous regions in other unannotated slices. SpaBatch also achieves robust clustering performance (median ARI = 0.361, representing a 38% improvement over the second‐best baseline), demonstrating its utility in pathological contexts. Downstream analyses reveal spatially variable genes (e.g., *TMSB10* in cortical layers of the DLPFC dataset,^[^ ^21^ ^]^ and *ERBB2* in tumor regions of HER2+ breast cancer dataset^[^ ^22^ ^]^), and Gene Ontology (GO) enrichment uncovers tissue‐specific biological processes (e.g., immune response within the tumor microenvironment). Overall, SpaBatch offers a flexible and effective tool for integrating ST data across multiple slices, enabling batch effect correction and enhancing the accuracy and stability of 3D spatial domain identification.

## 2 Experimental Section

### 2.1 Datasets and Data Preprocessing

We applied SpaBatch to eight ST datasets from different species, platforms, and abnormal tissue slices for integrated analysis to validate the model's performance (Table S1, Supporting Information).

(1) The human dorsolateral prefrontal cortex (DLPFC) dataset, measured by 10x Visium^[^ ^2^ ^]^ came from three independent neurotypical adult donors. Each donor including four adjacent slices, resulting in a total of 12 slices. The number of spots per slice ranges from 3498 to 4789. Manual spot‐level annotations from layer 1 to layer 6 and white matter (WM) provided by Maynard et al.^[^ ^21^ ^]^ were used as the ground truth.
(2) Section 1 of the sagittal mouse brain dataset, generated by the 10x Visium platform, comprises anterior and posterior brain slices with 2695 and 3355 spots, respectively. Notably, only the anterior slice in this section has manual annotations,^[^ ^23^ ^]^ which we adopt as the ground truth for evaluation. We additionally incorporated a mouse coronal brain dataset and integrated it with the mouse sagittal brain dataset. This dataset contains 2698 spots.
(3) Section 2 of the sagittal mouse brain dataset, generated by the 10x Visium platform, contains anterior and posterior slices with 2825 and 3289 spots, respectively.
(4) The coronal mouse whole brain dataset was provided by the ST platform, containing 35 coronal slices spanning the anterior–posterior (AP) axis.^[^ ^24^ ^]^ It includes manual annotations of 15 cluster types.^[^ ^25^ ^]^ These slices cover the entire brain region from the mouse olfactory bulb to the emergence of the cerebellum.
(5) The early mouse embryo dataset described by Stereo‐seq^[^ ^5^ ^]^ included four slices from the E9.5 stage (E2S1, E2S2, E2S3, and E2S4), with 5292, 4356, 5059, and 5797 spots, respectively. Manual annotations were provided for each slice.
(6) The human embryonic heart dataset was profiled by the 10x Visium platform.^[^ ^26^ ^]^ It includes two sets of slices from human embryonic hearts at 4.5‐5 post‐conception weeks (PCW) and 6.5 PCW. The 4.5‐5 PCW period includes four slices, each with approximately 60 spots. The 6.5 PCW period includes nine slices, with spots ranging from 100 to 212.
(7) The HER2‐positive breast cancer dataset was profiled by the 10x Visium platform.^[^ ^27^ ^]^ The dataset includes tumor samples from eight different patients, represented by groups A‐H. Groups A‐D each contain six slices, while groups E‐H each contain three slices. Only the first slice in each group has manual annotations.
(8) The mouse hypothalamic preoptic area data obtained by MERFISH^[^ ^28^ ^]^ sequencing technology includes three consecutive slices: hypothalamus‐0.09, hypothalamus‐0.14, and hypothalamus‐0.19, containing 5557, 5926, and 5803 spots respectively. Manual annotations were provided for each slice.^[^ ^29^ ^]^

The input of SpaBatch consists of the gene expression matrices of multiple slices and their spatial locations. First, we concatenate the gene expression matrices along the spot dimension to construct a multi‐slice gene expression matrix (hereafter denoted as the gene expression matrix). Genes expressed in fewer than 50 cells and with a total expression count less than 10 are removed. Subsequently, gene expressions are log‐transformed and normalized based on library size using the SCANPY^[^ ^30^ ^]^ package. Finally, top‐2000 highly variable genes are selected, and Principal Component Analysis (PCA) is applied to reduce the dimensionality of the gene expression matrix while preserving as much data variability as possible, denoted as *X* ∈ *R* ^*n* × *m*^.

### 2.2 Data Augmentation With Mask Mechanism

Spatial transcriptomics data are typically characterized by high sparsity and significant noise, which pose substantial challenges for robust feature learning. Traditional data augmentation methods, such as random dropout or noise injection, may disrupt the spatial structure or fail to effectively guide the model toward learning meaningful representations. Inspired by Masked Autoencoders (MAE),^[^ ^31^, ^32^ ^]^ we introduce a novel mask‐based augmentation strategy during the data preprocessing stage: this strategy randomly masks a subset of spots and replaces their gene expression vectors with learnable embedding vectors. This encourages the model to infer the expression of masked regions from their neighboring context, thereby enhancing its ability to cope with missing or corrupted data. Specifically, we randomly sample a set of masked vertices *V*_*m*_ from all spots based on the masking rate ρ. If *V*_*i*_ (spot *i*) belongs to *V*_*m*_, then $\tilde{x_{i}}=x_{\left[\text{mask}\right]}$, where *x*_[mask]_ denotes replacing the raw gene expression of spot *i* (*x*_*i*_) with a learnable vector; otherwise, $\tilde{x_{i}}=x_{i}$. Therefore, the gene expression matrix *X* is re‐defined as the masked gene expression matrix *X*_mask_ ∈ *R* ^*n* × *m*^, defined as:

(1) $$\tilde{x_{i}}=\left\{\begin{array}{cc} x_{\left[\text{mask}\right]}, & \text{if}\;V_{i}\in V_{m}\\ x_{i}, & \text{if}\;V_{i}\notin V_{m} \end{array}\right.$$

By adding a learnable vector to the masked spots, the model can dynamically adjust the representation of the masked spots during training, enabling them to learn features consistent with their surrounding neighbors. Through this mechanism, the model not only recovers the information of the masked spots but also enhances its generalization ability to the overall data.

### 2.3 Spatial Graph Construction

We construct a spatial graph by computing the Euclidean distances between spots and selecting the *k*‐nearest neighbors for each spot.^[^ ^23^ ^]^ If spot *i* and spot *j* are neighbors, then *A*_*ij*_ = *A*_*ji*_ = 1. The adjacency matrix is symmetric and stored in the form of a sparse matrix. We can adjust *k* so that the number of neighbors for each spot ranges between 6 and 12, making it adaptable to different ST scenarios and platforms.

We construct the adjacency matrix for spots in each slice separately by using the method described above, and then concatenate the adjacency matrices of each slice in a block diagonal form, denoted as *A* ∈ *R* ^*n* × *n*^. It is to combine information from different slices to better capture the spatial relationships across slices.

### 2.4 Latent Representation Learning Using SpaBatch

The latent representation learning of gene expression is achieved through a masked variational graph autoencoder (VGAE), which consists of an encoder and a decoder (Figure 1; Algorithm S1, Supporting Information). In the encoder, two fully connected layers are stacked to generate a low‐dimensional representation $Z_{f}\in R^{n\times d_{f}}$ from the masked gene expression matrix *X*_[*mask*]_ ∈ *R* ^*n* × *m*^. The Graph Convolutional Network (GCN) layer embeds the spatial graph *A* into *Z*_*f*_, capturing the spatial relationships among neighbors. The first GCN layer is used to learn a shared representation, following the approach of Kipf and Welling,^[^ ^33^ ^]^ and is expressed as:

(2) $$\begin{array}{cc} & Z_{g}^{\prime }=\text{GCN}\left(A,Z_{f}\right)=\text{ReLU}\left(\text{BN}\left(\hat{A}Z_{f}W_{0}\right)\right)\\ & \hat{A}=D^{-\frac{1}{2}}AD^{-\frac{1}{2}} \end{array}$$

where *D* is the degree matrix, and BN stands for Batch Normalization. The second GCN layer is formulated by:

(3) $$\begin{array}{cc} & \mu =GCN\left(A,Z_{g}^{\prime }\right)=\text{ReLU}\left(\text{BN}\left(\hat{A}Z_{g}^{\prime }W_{\mu }\right)\right)\\ & \log\left(\sigma^{2}\right)=GCN\left(A,Z_{g}^{\prime }\right)=\text{ReLU}\left(\text{BN}\left(\hat{A}Z_{g}^{\prime }W_{\sigma }\right)\right) \end{array}$$

with two different parameters, *W*_μ_ and *W*_σ_, to independently model the mean and variance.

Sampling directly from the distribution leads to non‐differentiable gradients. To construct *Z*_*g*_, the reparameterization trick is employed by:

(4) $$Z_{g}=\mu +\log\left(\sigma^{2}\right)*\varepsilon$$

where μ and log(σ^2^) are obtained from Equation (3), ε is a randomly generated Gaussian noise.

In SpaBatch, the final low‐dimensional latent embedding *Z* = *Z*_*f*_ + *Z*_*g*_. After obtaining the low‐dimensional latent embedding *Z*, an inner product decoder is used to reconstruct the adjacency matrix, denoted as $\tilde{A}$. To achieve this, the inner product decoder calculates the probability of an edge existing between each pair of nodes using the dot product of their respective latent vectors:

(5) $$P\left(A|Z\right)=\prod_{i=1}^{N}\prod_{j=1}^{N}p\left(A_{ij}|z_{i},z_{j}\right)$$

(6) $$\tilde{A}=p\left(A_{ij}=1|z_{i},z_{j}\right)=sigmoid\left(ZZ^{T}\right)$$

where *P*(*A*|*Z*) denotes the conditional probability of the observed adjacency matrix *A* given the latent variables *Z*. *z*_*i*_ and *z*_*j*_ are the latent representations of spot *i* and spot *j*, respectively, and *sigmoid*(·) ensures that ${\tilde{A}}_{ij}$ outputs a valid probability for the existence of an edge between spot *i* and spot *j*.

Then we use the following loss function to measure the differences between the approximated adjacency matrix $\tilde{A}$ and the raw adjacency matrix *A*:

(7) $$L_{graph}=||A-\tilde{A}{\left|\right|}^{2}$$

In addition to the reconstruction loss, the Kullback–Leibler (KL) divergence^[^ ^34^ ^]^ between the distribution of node representation vectors and a standard normal distribution is calculated. This term encourages the learned latent space to match a prior distribution. The KL divergence is given by:

(8) $$\begin{array}{cc} L_{KL}= & E_{q(Z|X_{\left[mask\right]},A)}\left[\log\;p\left(A|Z\right)\right]-KL[q\left(Z|X_{\left[mask\right]},A\right)||p\left(Z\right)] \end{array}$$

where $E_{q(Z|X_{\left[mask\right]},A)}\left[log\;p\left(A|Z\right)\right]$ is the binary cross‐entropy and $p\left(Z\right)=\prod_{i}N\left(0,I\right)$.

The decoder part employs a single layer of GCN to reconstruct the raw input gene expression matrix from the latent representation *Z*, denoted as $\tilde{X}$. The GCN layer can capture local and global dependencies within the gene expression data, thereby assisting in the accurate reconstruction of the raw gene expression matrix. The reconstruction loss is constructed under a masked self‐supervision framework by minimizing the difference between *X*_[*mask*]_ and ${\tilde{X}}_{\left[mask\right]}$. By employing the Scaled Cosine Embedding (SCE) loss as the objective function, it is formulated with a predefined scaling factor γ in the following manner:

(9) $$L_{sce}=\frac{1}{|V_{m}|}\sum_{v_{i}\in V_{m}}{\left(1-\frac{x_{[mask]i}{\tilde{x}}_{[mask]i}^{T}}{\left\|x_{[mask]i}\right\|\left\|{\tilde{x}}_{[mask]i}\right\|}\right)}^{\gamma },\gamma \geq 1$$

where γ is used to adjust the model's sensitivity to larger errors. $|V_{m}|$ is the number of spots in the masked set. *x*_[mask]*i*_ and ${\tilde{x}}_{[\text{mask}]i}$ represent the feature vectors of the *i*‐th spot in *X*[mask] and $\tilde{X}\left[\text{mask}\right]$, respectively.

We construct a Variational Graph Autoencoder (VGAE), whose overall loss function consists of three components:

(10) $$L_{\text{VGAE}}=L_{\text{graph}}+L_{\text{KL}}+L_{\text{sce}}$$

The objective of model training is to minimize this joint loss function, thereby simultaneously achieving graph structure preservation and latent space regularization.

#### 2.4.1 Self‐Supervision Module

During the pretraining phase, SpaBatch learns the low‐dimensional embeddings of gene expression in the latent space *Z* through a variational graph autoencoder (VGAE). Subsequently, deep embedded clustering (DEC)^[^ ^35^ ^]^ is introduced to refine the local clustering details of *Z*. In the formal training phase, the model defines a clustering layer in the latent space, represented as ${\left\{\phi_{j}\right\}}_{j=1}^{J}$, where *J* denotes the number of clusters. The self‐supervised module first performs K‐means clustering on *Z* and initializes the cluster centroids as the mean of samples in each cluster. These centroids are stored in the clustering layer and are further refined through iterative optimization to enhance clustering accuracy.

We use the Student's *t*‐distribution similarity^[^ ^36^ ^]^ to quantify the relationship between spots and cluster centroids. Based on this similarity, it is transformed into the probability distribution *q*_*ij*_ that the spot *i* belongs to the specific cluster *j*, as defined by the following formula:

(11) $$q_{ij}=\frac{{\left(1+||z_{i}-\phi_{j}|{|}^{2}\right)}^{-1}}{\sum_{j^{\prime }}{\left(1+||z_{i}-\phi_{j^{\prime }}|{|}^{2}\right)}^{-1}}$$

where *z*_*i*_ represents the embedding vector of spot *i* in the low‐dimensional embedding *Z*, and ϕ_*j*_ corresponds to the *j*‐th cluster centroid. The value *q*_*ij*_ is used to compute the soft assignment probability, describing the likelihood of spot *i* being assigned to cluster *j*.

Additionally, the self‐supervised module generates a target distribution by assigning higher weights to high‐confidence samples. This distribution is constructed by enhancing the peaks of the current soft assignment distribution, aiming to improve the model's ability to distinguish between different clusters. The target distribution *p*_*ij*_ is defined as:

(12)

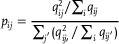

where *p*_*ij*_ represents the probability of assigning the *i*‐th spot to the *j*‐th cluster in the target distribution. The term ∑_*i*_ *q*_*ij*_ represents the total assignment probability for cluster *j*, normalized across all spots to ensure a valid probability distribution.

The self‐supervised module minimizes the Kullback–Leibler (KL) divergence between the target distribution *p*_*ij*_ and the soft assignment probability *q*_*ij*_. The objective function is defined as:

(13) $$L_{DEC}=KL\left(P|Q\right)=\sum_{i}\sum_{j}p_{ij}log\frac{p_{ij}}{q_{ij}}$$

#### 2.4.2 Triplet Learning Based on Readout Aggregation Strategy

To address batch effects across multiple ST slices, inspired by Deep Graph Infomax (DGI),^[^ ^37^ ^]^ we apply a triplet learning framework based on a readout aggregation strategy. Specifically, we first establish pairwise relationships between slices and compute the cosine similarity between spots from different pairs based on their low‐dimensional embeddings. If a spot *i* in slice A and a spot *j* in slice B appear in each other's nearest neighbor sets, they are defined as mutual nearest neighbors (MNNs) and selected as anchor points.

Unlike STAligner,^[^ ^16^ ^]^ which relies on pointwise MNN pairs and is potentially sensitive to misaligned matches, the readout aggregation strategy of SpaBatch captures the local microenvironment around the positive samples, thereby addressing key limitations of STAligner: (1) It mitigates the impact of individual erroneous MNNs by emphasizing the spatial consistency of the neighborhood, rather than relying solely on isolated pairwise matches, thus enhancing robustness to spatial noise; (2) Even if some true correspondences are not identified as MNNs, their information may still be incorporated indirectly if they lie within the aggregated neighborhood.

For anchor *i*, we construct a cross‐slice adjacency graph for each paired relationship between the slice to which *i* belongs and other slices, using the low‐dimensional embeddings obtained from the pre‐training stage. (1) We first select the α most similar neighbors in the cross‐slice adjacency graph of anchor *i*, forming the positive sample set $S_{i}^{+}$. (2) We then aggregate $S_{i}^{+}$ into a single positive representation $z_{i}^{+}=\text{Readout}\left(S_{i}^{+}\right)=\frac{1}{\alpha }\sum_{z\in S_{i}^{+}}$, which encodes the microenvironment of the positive neighborhood. (3) Similarly, we construct the negative sample set $S_{i}^{-}$ by randomly sampling α dissimilar spots, and aggregate them into a single negative representation $z_{i}^{-}=\text{Readout}\left(S_{i}^{-}\right)=\frac{1}{\alpha }\sum_{z\in S_{i}^{-}}$. (4) The triplet loss is employed to minimize the distance between anchor‐positive pairs and maximize the distance between anchor‐negative pairs in the latent space:

(14) $$\begin{array}{c} L_{\text{Tri}}=\frac{1}{N_{\text{Tri}}}\sum_{i=1}^{N_{\text{Tri}}}\max\left({\left\|z_{i}-z_{i}^{+}\right\|}_{2}-{\left\|z_{i}-z_{i}^{-}\right\|}_{2}+\tau ,\;0\right) \end{array}$$

where *z*_*i*_, $z_{i}^{+}$, and $z_{i}^{-}$ represent the anchor point, the positive sample, and the negative sample, respectively. *N*_Tri_ denotes the number of triplets set. τ is the margin (with a default value of 1.0), ensuring that the distance difference between negative samples is sufficiently large.

### 2.5 Overall Loss Function

In the pretraining phase, we optimize only the loss functions $L_{VGAE}$ to obtain the low‐dimensional embeddings of spots in the latent space. In the training phase, we optimize the VGAE loss while updating the self‐supervised module every 20 epochs and the triplet loss every 500 epochs, ultimately obtaining the final latent embeddings. The overall loss function is represented as:

(15) $$\begin{array}{c} L_{overall}=\lambda_{1}L_{VGAE}+\lambda_{2}L_{DEC}+\lambda_{3}L_{Tri} \end{array}$$

where $L_{VGAE}$ includes three loss functions in the backbone network: $L_{graph}$, $L_{KL}$, and $L_{sce}$. The coefficients λ_1_, λ_2_, and λ_3_ balance the contributions of VGAE loss, DEC loss, and triplet loss, respectively. They are set empirically to ensure effective representation learning, clustering, and batch effect correction.

### 2.6 3D Spatial Clustering

After learning latent representations from the ST data with SpaBatch, low‐dimensional embeddings are generated and subsequently clustered to identify spatial domains. Clustering is performed using the mclust package (v6.0.1) in R.^[^ ^38^ ^]^ To comprehensively assess clustering performance, SpaBatch adopts multiple evaluation metrics, including the Adjusted Rand Index (ARI), Average Clustering Consistency (ACC), and V‐measure.

### 2.7 Evaluation Criteria

**ARI**. The Adjusted Rand Index (ARI)^[^ ^39^ ^]^ can be used to measure the similarity between the clustering outcomes and manual annotations. The range of ARI is [− 1, 1], where values closer to 1 indicate better clustering performance, and values closer to 0 suggest that the clustering is similar to random assignment. The ARI is calculated as follows:

(16) $$ARI=\frac{TP+TN-E}{TP+TN+FP+FN-E}$$

where *TP*, *TN*, *FP*, and *FN* are the number of true positive, true negative, false positive and false negative sample pairs, respectively. *E* is the expected similarity, it is calculated as follows:

(17) $$E=\frac{\left(TP+FP)\times (TP+FN)+(FN+TN)\times (FP+TN\right)}{TP+TN+FP+FN}$$

**ACC**. Average Clustering Consistency (ACC) is also used to assess the consistency of clustering results, by combining Normalized Mutual Information (NMI) and Adjusted Mutual Information (AMI) as follows:

(18) $$ACC=\frac{NMI+AMI}{2}$$

NMI measures the amount of shared information between two clustering results and ranges from [0, 1], with larger values indicating better clustering. AMI, as an adjusted version of mutual information, removes the influence of randomness and ranges from [− 1, 1], with larger values indicating better clustering.^[^ ^39^ ^]^

**V‐measure**. V‐measure is a clustering evaluation metric based on information theory.^[^ ^40^ ^]^ It consists of two components: Homogeneity (HOM) and Completeness (COM). V‐measure calculates the harmonic mean of these two metrics to comprehensively assess the consistency and completeness between the clustering results and the manual annotations, thereby evaluating the quality of the clustering. The value of V‐measure ranges from [0, 1], with a higher value indicating better clustering results. It is calculated as follows:

(19) $$\text{V-measure}=\frac{2\cdot HOM\cdot COM}{HOM+COM}$$

**iLISI and cLISI**. Integration Local Inverse Simpson's Index (iLISI) and Cell‐type Local Inverse Simpson's Index (cLISI) are two important metrics used to evaluate the integration performance of single‐cell or spatial transcriptomics data. Derived from the LISI (Local Inverse Simpson's Index) framework, these metrics are primarily designed to assess the effectiveness of batch effect removal and the preservation of cell types (or spatial domains) after data integration.^[^ ^41^ ^]^

iLISI is used to evaluate the effectiveness of batch effect removal after data integration. It measures the degree of mixing of different batches within the local neighborhood of each cell. A higher iLISI value indicates better batch mixing. cLISI is used to assess the separation of cell types (or spatial domains) after data integration. It measures the homogeneity of cell types within the local neighborhood of each cell. A lower cLISI value indicates better separation of cell types (or spatial domains).

### 2.8 Implementation Details

In this study, all experiments were conducted on a single NVIDIA RTX 4090Ti GPU. The mask rate was set to 0.2. We adjusted the parameter *k*, used to construct the spatial graph, setting the number of neighbors between 6 and 12, making it adaptable to different ST scenarios and platforms. According to our tests, when *k* is set to 8, SpaBatch achieves the best performance across most datasets. The fully connected layers of the encoder had dimensions of 64 and 16, while the graph convolution layers were set to 64 and 16. The number of clustering centroids in the self‐supervised module was set to 20. The learning rate and weight decay were set to 5e‐4 and 1e‐4, respectively, and optimization was performed using Adam. We recorded the running time and memory usage of SpaBatch under different datasets. As the number of spots increased, both computation time and memory consumption rose steadily, indicating that SpaBatch demonstrates good resource utilization efficiency. On a spatial transcriptomics dataset with approximately 20 000 spots, the training process typically completed within 4 minutes, with memory usage around 15 GB (Table S2, Supporting Information).

### 2.9 Baseline Methods

We compared SpaBatch with state‐of‐the‐art methods (Table S3, Supporting Information). Here are the descriptions of the methods and the parameter settings:

- **Scanpy** ^[^ ^30^ ^]^ tool performs standard preprocessing on the raw ST data, including normalization, log‐transformation, and selection of 3000 highly variable genes for subsequent dimensionality reduction and clustering analysis. To reduce dimensionality, principal component analysis (PCA) reduces the highly variable gene expression matrix to 20 dimensions. Finally, the mclust clustering algorithm is applied in this low‐dimensional space to identify spatial domains in the ST data. This process does not involve explicit nonlinear dimensionality reduction or batch effect correction steps.
- **STAligner** ^[^ ^16^ ^]^ is an extension of STAGATE^[^ ^8^ ^]^ designed for multi‐slice ST integration tasks, and it demonstrates superior performance compared to STAGATE in multi‐slice settings (Figures S1 and S2, Supporting Information). While STAGATE is based on graph attention networks and effectively identifies spatial domains within individual tissue slices, STAligner builds upon this framework by introducing a cross‐slice batch effect correction mechanism based on mutual nearest neighbors (MNNs). STAligner adopts STAGATE as the backbone network to learn low‐dimensional embeddings of gene expression and performs batch effect correction in the latent space. Data preprocessing follows the default settings of STAligner, selecting the top 5000 highly variable genes as input features. The “rad_cutoff” parameter is adjusted dynamically for each dataset to ensure that the number of neighbors per spot falls between 6 and 12, thereby achieving optimal model performance.
- **STG3Net** ^[^ ^17^ ^]^ uses a masked graph convolutional autoencoder as the backbone module, combined with generative adversarial learning and a global neighbor selection strategy to construct triplets for robust multi‐slice spatial domain identification and batch correction. We used the default parameter settings.
- **SEDR** ^[^ ^23^ ^]^ is a graph neural network‐based spatial embedding method. SEDR applies Harmony^[^ ^42^ ^]^ to perform batch correction on the learned low‐dimensional embeddings. We followed the parameter settings provided in the authors' code, specifically setting “using_dec” to False and the number of “epochs” to 200.
- **DeepST** ^[^ ^10^ ^]^ extracts features from H&E images based on spatial location information and constructs a neighbor graph from the image features to enhance gene expression. Since DeepST requires H&E images as input, we applied it only to the DLPFC and sagittal mouse brain datasets. We used the default parameters set in the demonstration code in the online tutorial.
- **SpaGIC** ^[^ ^43^ ^]^ learns meaningful point latent embeddings by maximizing the mutual information between edges and local neighborhoods of the graph structure and minimizing the embedding distance between spatially adjacent points. It integrates graph convolutional networks and self‐supervised contrastive learning techniques. SpaGIC uses Harmony to batch‐correct the learned low‐dimensional embeddings. We set “mse_weight,” “graph_weight,” and “nce_weight” to 60, 0.01, and 0.01, respectively. The number of “epochs” was set to 500, and “n_neighbor” was set to 5, all consistent with the default settings.
- **STitch3D** ^[^ ^18^ ^]^ leverages the ICP or PASTE algorithm to optimize the alignment between multiple slices. It incorporates both slice‐spot and slice‐gene factors, and reconstructs gene transcription expression by utilizing cell composition components informed by scRNA‐seq data.

## 3 Results

### 3.1 SpaBatch Effectively Corrects Batch Effects and Precisely Identifies Spatial Domains in the DLPFC Dataset

To quantitatively evaluate SpaBatch in spatial domain identification and batch effect correction for multi‐slice joint analysis, we first applied it to the human dorsolateral prefrontal cortex (DLPFC) dataset measured by 10x Genomics Visium.^[^ ^2^ ^]^ We compared SpaBatch with six state‐of‐the‐art methods, evaluating their performance in spatial domain identification by metrics of ARI, ACC and V‐Measure. Additionally, we assessed their performance in batch effect correction and multi‐slice integration results by metrics of iLISI and cLISI.^[^ ^41^ ^]^

**Figure 2:**
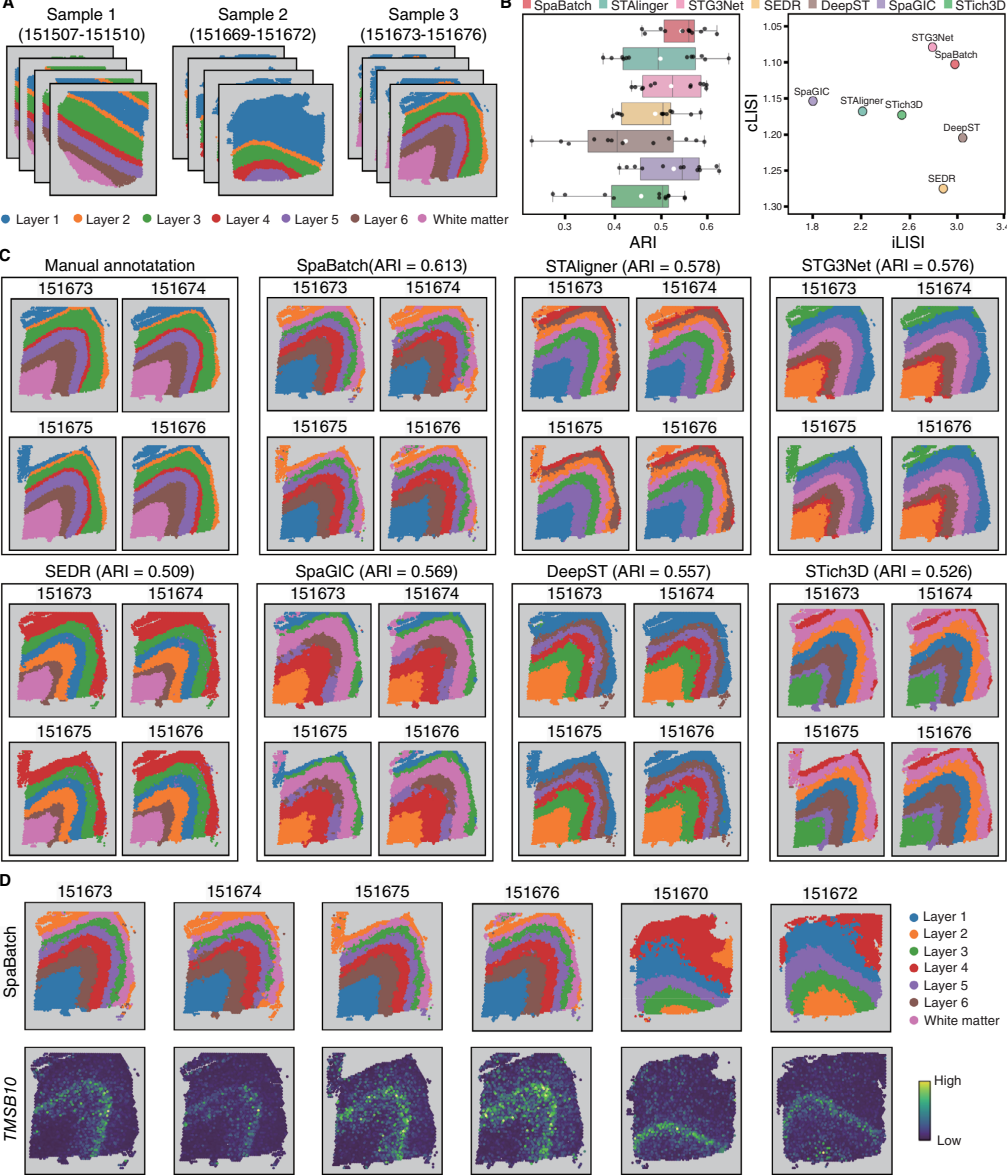
Results of multi‐slice joint analysis on the DLPFC dataset. A) Three samples of the DLPFC dataset and their manual annotations. B) Boxplots of ARI values calculated by SpaBatch and other methods across 12 slices in three samples of the DLPFC dataset. In the boxplots, the central line and the solid white dot represent the median and the mean, respectively. The swarm plot illustrates the accuracy distribution across all slices (left). The iLISI and cLISI scores calculated for SpaBatch and other methods on three samples of the DLPFC dataset are shown (right). The x‐axis represents batch mixing scores, and the y‐axis represents spatial domain mixing scores. Points closer to the top‐right corner indicate better performance. C) Results of SpaBatch and baseline methods in integrating the four slices of Sample 3 from the DLPFC dataset and identifying the spatial domains. D) Spatial domains identified by SpaBatch across six slices (top) and the spatial expression of the layer 5 marker gene *TMSB10* (bottom).

We divided the samples from different donors into three groups, with each sample containing four adjacent slices (**Figure** **2**A). We first evaluated the performance on sample 3 and observed that the spatial domains identified by SpaBatch, STAligner, and STG3Net were well‐mixed within the same cortical layer, while different cortical layers were ordered according to the spatial structure of layer 1 to layer 6 and the white matter layer (WM). Compared to the latter two methods, SpaBatch produced more precise boundaries and shapes. Other methods exhibited inaccuracies in spatial domain identification within layers 1‐4. SpaBatch achieved the highest clustering accuracy in terms of ARI, with an average value of 0.613, surpassing STAligner (ARI = 0.578) and STG3Net (ARI = 0.576), and significantly outperforming the other methods (Figure 2C). We also conducted the same experiment on sample 1 and sample 2. The results showed that SpaBatch outperformed other methods in the 3D spatial domain identification (Figure S3, Supporting Information). We conducted experiments on samples 1‐3 respectively and calculated the ARI values for all the 12 slices (Table S4, Supporting Information). The results demonstrated that SpaBatch outperformed all other methods, achieving the highest median and mean ARI scores, thereby further highlighting its advantage in spatial domain identification on the DLPFC dataset compared to other methods (Figure 2B; Figure S4A, Supporting Information). Additionally, we further tested the robustness of SpaBatch by comparing the clustering accuracy across different random seeds and found that SpaBatch was not sensitive to variations in random seeds (Figure S4B, Supporting Information).

**Figure 3:**
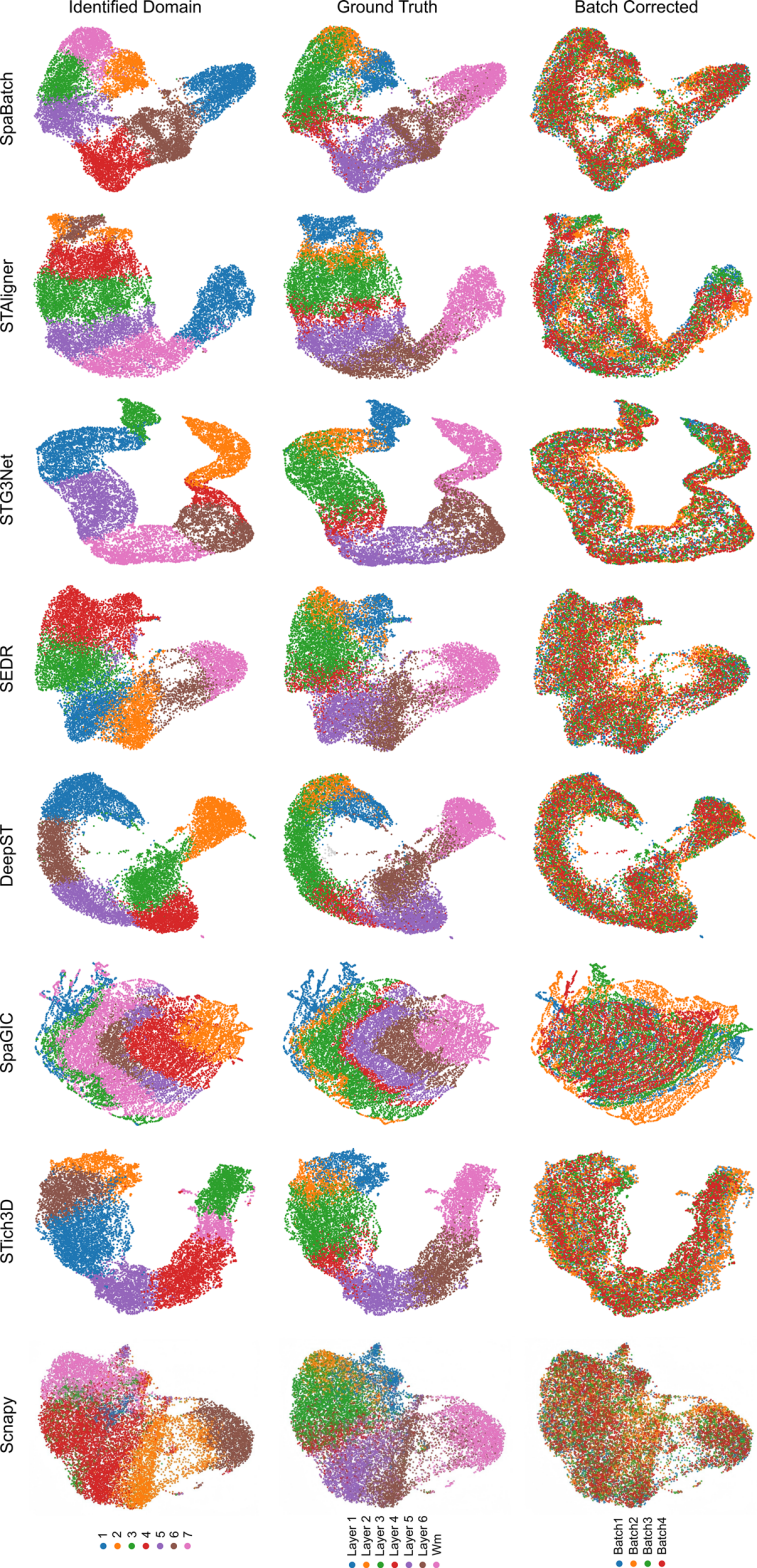
UMAP visualization of sample 3 (slices 151673‐151676) embeddings colored by identified domains (left), ground truth (middle), and batch corrected (right).

The Uniform Manifold Approximation and Projection (UMAP)^[^ ^44^ ^]^ visualization results from Scanpy showed no obvious separation between batches in the UMAP space, indicating that batch effects in the DLPFC dataset were not significant (Figure 3). However, compared to deep learning‐based models, Scanpy's clustering boundaries were relatively blurred, and its clustering results exhibited poorer compactness and spatial coherence. This highlights the limitations of traditional methods in handling complex spatial transcriptomics patterns. Other deep learning methods produced well‐organized layer structures that highly corresponded with manual annotations. In particular, SpaBatch demonstrated outstanding capability in accurately capturing spatial domain structures, with its UMAP visualization closely matching the manually annotated layer distributions. Moreover, SpaBatch achieved smooth and uniform mixing across different batches, which was well reflected in the cross‐slice batch integration. It is noteworthy that although some deep learning models, such as STAligner and SpaGIC, improved clustering compactness to some extent, their batch‐corrected UMAP visualizations still showed a tendency for samples from different batches to separate again. This “over‐correction” phenomenon suggests that these models may have overemphasized batch correction during the integration process. The values of iLISI and cLISI also indirectly demonstrated the ability of SpaBatch to achieve effective slice mixing and batch effect correction. While the higher cLISI score of STG3Net highlighted its strength in batch effect correction, it came at the expense of reduced iLISI, indicating potential loss of biological signals. On the other hand, the higher iLISI score of DeepST reflected better preservation of biological signals but revealed poor performance in cLISI, suggesting insufficient batch effect correction. SpaBatch demonstrated a balanced performance, excelling in cLISI while maintaining a high iLISI score. This balance showcased the ability of SpaBatch to perform batch effect correction effectively while preserving critical biological signals (Figure 2B right). SpaBatch not only achieved higher accuracy in spatial domain identification but also ensured that the integration across batches maintained a high degree of consistency and coherence. This advantage continued to hold across other samples (Figures S5 and S6, Supporting Information).

We further identified layer‐marker genes by performing differential expression analysis and previous reports,^[^ ^21^ ^]^ such as *AQP4* (layer 1), *CARTPT* (layer 2), *ENC1* (layer 3), *PCP4* (layer 4), *TMSB10* (layer 4 and layer 5), and *MBP* (WM), which exhibited significant expression differences across different cortical layers (Figure S7, Supporting Information). By visualizing the layer‐marker gene *TMSB10* in layer 5, we demonstrated that SpaBatch can effectively identify layer‐marker genes and depict the shared organizational structures between different samples (Figure 2D).

### 3.2 SpaBatch Comprehensively Depicts the Mouse Brain From Both Macro and Micro Perspectives

**Figure 4:**
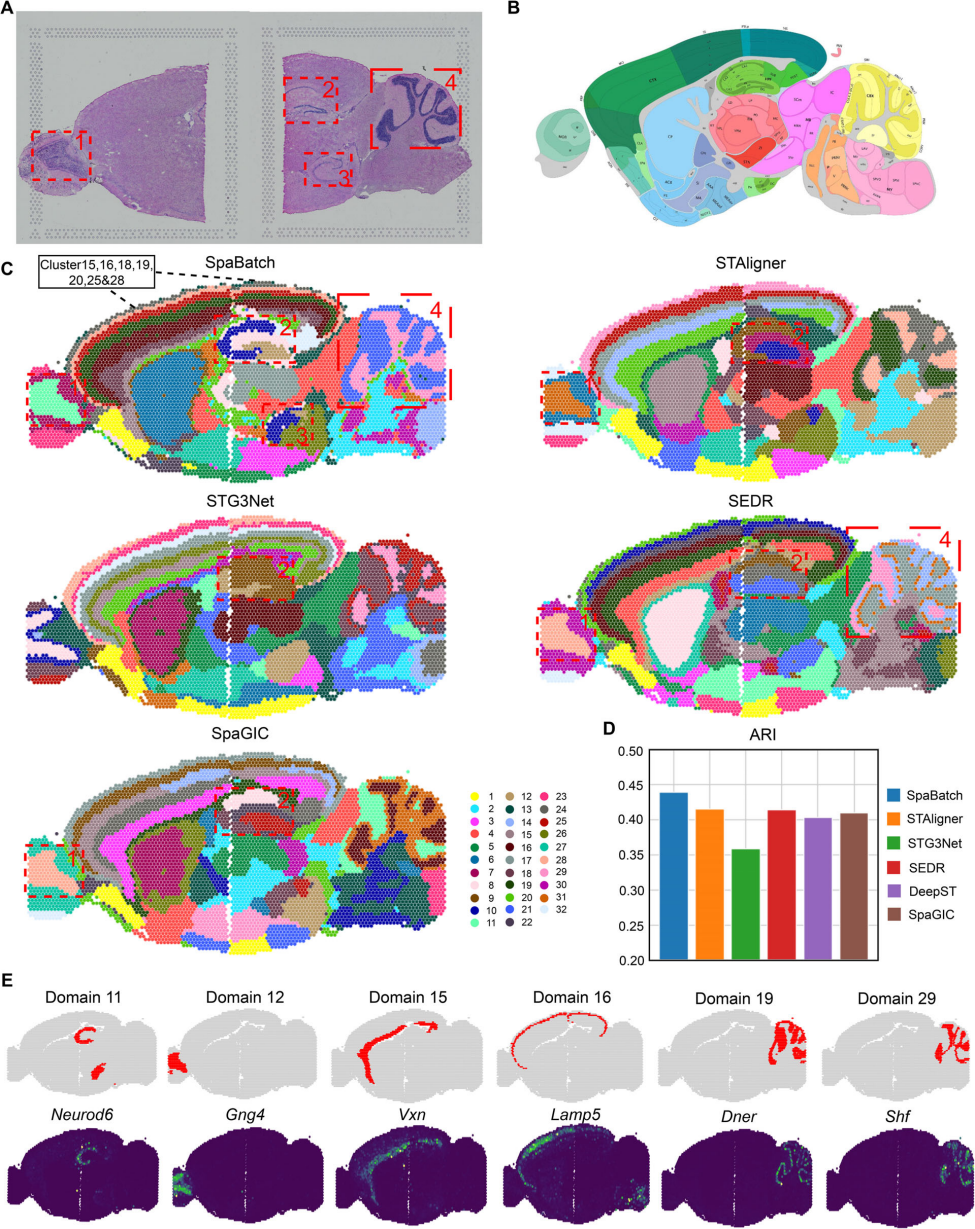
SpaBatch comprehensively depicts the sagittal mouse brain from the macro perspective. A) H&E images of sagittal anterior and posterior sections of Section 1, and the corresponding specific spatial subdomains. B) Manual annotation of the sagittal anterior mouse brain (Section 1), from the Allen Mouse Brain Reference Atlas. C) The spatial domain identification results of sagittal mouse brain Section 1 integrated by SpaBatch and other methods. We used red boxes and numbers to highlight specific spatial subdomains. D) The bar plot comparing the ARI values obtained from SpaBatch and other methods with the manual annotation. E) SpaBatch identifies six distinct fine spatial subdomains (top) and the spatial expression of marker genes associated with these subdomains (bottom).

Next, we evaluated the performance of SpaBatch on more complex tissue sections. We conducted experiments on Sections 1 and 2 of sagittal mouse brain data (**Figure** **4**A; Figures S8A and S9A, Supporting Information) generated by the 10x Visium protocol.^[^ ^2^ ^]^ Both Sections 1 and 2 contain paired samples of the anterior and posterior brain. We integrated the anterior and posterior regions of Sections 1 and 2 separately for the experiments. Among the two datasets, only the anterior region of Section 1 has manual annotations, which we used as ground truth (Figure S8B, Supporting Information). The data provided an overall anatomical structure for understanding the mouse brain's organization from a global perspective.

We first tested the integration capabilities of SpaBatch, STAligner, STG3Net, SEDR, and SpaGIC using Section 1. For comparison, the number of clusters was set to 32 for all methods. The mouse brain atlas provided by Allen Brain Atlas^[^ ^45^ ^]^ was used as a reference (Figure 4B). We found that SpaBatch, STAligner, STG3Net, and SEDR were all able to identify shared clusters between adjacent regions of consecutive slices in Section 1 and Section 2, primarily including the cortex, hippocampus, thalamus, and hypothalamus. For more delicate tissue structures, SpaBatch has a stronger detection capability. The main olfactory bulb in the anterior and the cerebellar cortex in the posterior (red box 1 and 4 of Figure 4C) detected by SpaBatch show high consistency with the Allen Brain Atlas mouse cortex reference atlas. Only SEDR identified both spatial domains simultaneously, but its accuracy in the cerebellar cortex region was still inferior to SpaBatch. Moreover, we further examined the dorsal and ventral regions of the hippocampus located in the posterior brain (red box 2 and 3 of Figure 4C). These regions span both anterior and posterior brain areas, posing a greater challenge for the continuous identification of spatial domains. The dorsal hippocampal region consists of CA1, CA2, CA3, and the dentate gyrus (DG), which together form the classic trisynaptic circuit. This circuit is crucial for memory formation and retrieval.^[^ ^46^ ^]^ Among all methods, only SpaBatch accurately identified the ventral region of the hippocampus and fully recognized the ammonic horns (CA), composed of CA1, CA2, and CA3, as well as the dentate gyrus (DG). Other methods failed to achieve accurate identification in this region. Finally, for the cortical regions shared by two slices, SpaBatch is able to comprehensively identify and accurately distinguish different subdomains, including the molecular layer (layer 1), external granular and pyramidal layers (layer 2/3), internal granular layer (layer 4), internal pyramidal layer (layer 5), and polymorphic layer (layer 6a and layer 6b), which align with the defined spatial domains 15, 16, 18, 19, 25, and 28 (Figure 4C). In terms of the ARI scores on the anterior brain slice of Section 1, SpaBatch achieved the highest performance (ARI = 0.44), outperforming all other methods (Figure 4D).

In addition, SpaBatch imputed gene expression and enhances the signals of domain marker genes. For example, the expression of the *Neurod6* gene aligned with the CA region identified in domain 11, while *Vxn* and *Lamp5* corresponded to domains 15 and 16, representing the layer 2/3 and layers 6a, 6b, respectively. Furthermore, the combination of *Dner* and *Shf* fully represented the cerebellar region identified in domains 19 and 29 (Figure 4E; Figure S8C,D, Supporting Information). This example further highlighted ability of SpaBatch to provide a comprehensive depiction of the sagittal mouse brain while mitigating batch effects, a strength that persisted in Section 2 (Figure S9, Supporting Information).

**Figure 5:**
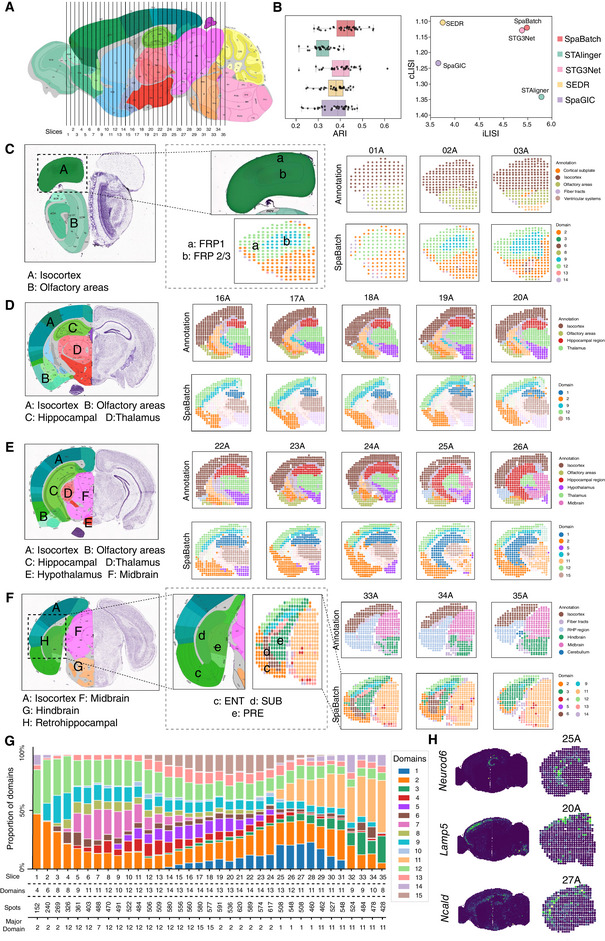
SpaBatch comprehensively depicts the sagittal mouse brain from the micro perspective. A) The locations of the 35 slices were mapped to the Allen Brain Atlas reference based on the spatial domain results identified by SpaBatch and manual annotations. B) The box plot of ARI values computed by SpaBatch and other methods on the 35 coronal mouse brain slices is shown (left). It displays the iLISI and cLISI scores calculated by SpaBatch and other methods on this dataset (right). C–F) SpaBatch accurately identifies spatial domains in the coronal mouse brain across different slices along the anterior‐posterior (AP) axis and corresponds to specific tissue structures through the Allen Brain Atlas reference. SpaBatch can also further identify regions that were not delineated in the manual annotations. G) The stacked bar plot of spatial domain proportions for the 35 slices by SpaBatch. H) The domain marker gene *Neurod6* in the hippocampus and the domain marker genes *Lamp5* and *Ncald* in the cortex exhibit the same spatial pattern in both sagittal and coronal mouse brain slices.

To further explore the adult mouse brain at a microscopic level, we collected 35 coronal slices spanning the anterior‐posterior (AP) axis,^[^ ^24^ ^]^ which include manual annotations of 15 clustering types.^[^ ^25^ ^]^ These slices cover the entire brain region from the mouse olfactory bulb to the emergence of the cerebellum (Figure S10, Supporting Information). Based on our clustering results and manual annotations, we mapped the locations of these coronal slices onto the Allen Brain Atlas reference and sagittal mouse brain slices obtained through SpaBatch (**Figure** **5**A; Figure S11, Supporting Information). This mapping provides a more comprehensive understanding of the spatial distribution and organization of brain regions in different anatomical planes. By analyzing these slices, we are able to observe the gradual changes in the adult mouse brain from a more microscopic perspective at different anterior‐posterior locations. The multi‐slice joint analysis task is challenging as it requires methods to account for batch effects across dozens of slices and to distinguish subtle variations in spatial domains within the adult mouse brain.

The spatial domain variations identified by SpaBatch across the 35 slices exhibited strong connectivity (Figure S11C,D, Supporting Information), and all these variations were validated within the Allen Brain Atlas reference. In the earliest adult mouse brain slices (01A‐03A), the regions primarily include the isocortex and olfactory areas. SpaBatch not only accurately identified these two regions but also detected areas that were not differentiated in the manual annotations. Specifically, it precisely subdivided the isocortex into FRP 1 and FRP 2/3 (Figure 5C). As the hippocampus gradually expanded (16A‐20A), SpaBatch accurately captured four key spatial domains, which closely aligned with manual annotations (Figure 5D). As the hippocampus continued to expand, the hypothalamus gradually shrank, and the midbrain began to emerge (22A‐26A). These dynamic changes were clearly detected by SpaBatch (Figure 5E). As the Hippocampus transitions to Retrohippocampal (33A‐35A), SpaBatch divides this area into ENT (entorhinal cortex), SUB (subiculum), and PRE (pre‐subiculum) (Figure 5F), which were not differentiated in the manual annotations. These regions play a crucial role in spatial perception, memory formation, and the flow of information between the cortex and the hippocampus.^[^ ^47^ ^]^ By analyzing the clustering proportions across all 35 coronal slices, SpaBatch offered a clear visualization of tissue composition and its dynamic changed throughout the mouse brain. For example, the hippocampal domain emerges at slice 15A and disappears after slice 31A (Figure 5G). Notably, in the sagittal mouse brain slices, SpaBatch identified several spatially specific marker genes, including *Neurod6* (hippocampus), *Lamp5*(cortex), and *Ncald* (cortex). Visualization of these marker genes revealed that their spatial expression patterns were consistently preserved across both sagittal and coronal sections of the mouse brain (Figure 5H). The consistent spatial expression patterns observed across different slicing orientations indicate that SpaBatch effectively corrects batch effects and enables cross‐slice 3D spatial domain identification with coherent marker gene expression, further demonstrating the biological relevance of the spatial domains identified by SpaBatch. Finally, SpaBatch achieved the best performance in clustering and batch effect correction metrics compared to the baseline methods (Figure 5B and Figure S11B, Supporting Information).

In summary, SpaBatch provided a comprehensive depiction of the adult mouse brain's spatial domains from both macroscopic and microscopic perspectives by analyzing sagittal and coronal mouse brain data. In the sagittal mouse brain, it effectively integrated regions across different brain areas and detected complex structures of cross‐slice spatial domains, such as the hippocampus and cerebellar cortex. In the 35 coronal slices spanning the anterior‐posterior axis, SpaBatch offered a clear spatial distribution map of mouse brain regions, revealing dynamic changes in structures like the hippocampus and cortex, and links the two datasets through marker genes specific to each spatial domain. With its superior 3D spatial domain identification and batch effect correction capabilities, SpaBatch outperformed other methods across multiple evaluation metrics, highlighting its potential as a powerful tool for spatial transcriptomics analysis.

### 3.3 Identifying Shared and Correlated Spatial Domains Between Multiple Tissue Slices of E9.5 Mouse Embryos Using SpaBatch

We also applying SpaBatch to the early mouse embryo dataset generated by the Stereo‐seq platform, to identify shared spatial domains across four slices (E2S1, E2S2, E2S3, and E2S4) from the same developmental stage (E9.5).

**Figure 6:**
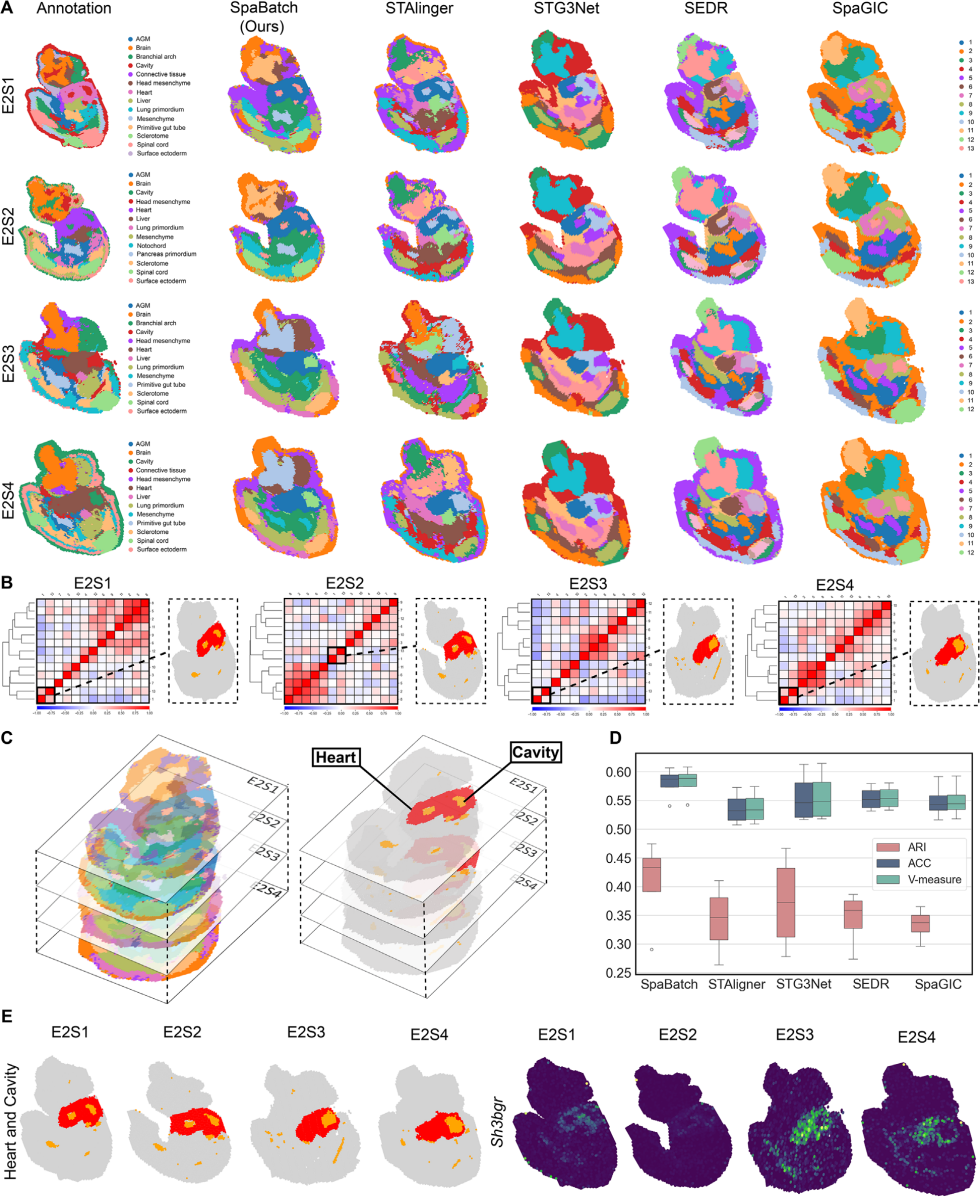
SpaBatch cross‐slice matching of shared spatial domains on the mouse embryo dataset. A) Spatial domains identified by SpaBatch and other methods on the E2S1, E2S2, E2S3, and E2S4 slices of the E9.5 embryo. B) SpaBatch identified correlated spatial domains 1 and 13 across the E2S1, E2S2, E2S3, and E2S4 slices of the E9.5 embryo through a correlation heatmap, and these domains highly overlap with the manually annotated heart and cavity regions. C) Spatial domains were identified by SpaBatch across all four slices, E2S1, E2S2, E2S3, and E2S4 (left). The regions of correlation between the heart and cavity identified by SpaBatch on all four sections (right). D) Box plots of ARI, ACC, and V‐measure calculated by SpaBatch and other methods on the mouse embryo E2S1, E2S2, E2S3, and E2S4 slices. E) SpaBatch identified spatial domains related to the heart and cavity across all four slices (left), along with the spatial expression of the marker gene *Sh3bgr* associated with this region (right).

Despite the spatial differences in tissue structure and the presence of significant batch effects across these four sections, SpaBatch can effectively integrate the four sections into the embedding space and align the spatial domains (**Figure** **6**A). We compared the performance of SpaBatch with STAligner, STG3Net, SEDR, and SpaGIC in integrating the four consecutive tissue sections. Our analysis indicated that SpaBatch was the only method capable of fully identifying the heart region in all four sections. It demonstrated exceptional ability to align spatial domains, effectively integrating data from all four sections. In contrast, STAligner emerged as the most competitive method, showing excellent performance in aligning and integrating tissue sections, although it could not fully capture the heart region across all slices as SpaBatch did. SpaBatch not only comprehensively identified the heart region in the mouse embryo but also excelled in aligning other tissues. It successfully recognized additional spatial domains across the four consecutive sections, including Mesenchyme (domain 8), Sclerotome (domain 9), Primitive gut tube (domain 10), Brain (domain 11) and Spinal cord (domain 12) (Figure S12A– C, Supporting Information). SpaBatch shows significantly better ARI, ACC, and V‐measure on the four slices compared to the baseline methods, indicating its more precise recognition of spatial structures (Figure 6D). This ability to accurately capture and align various tissue structures across different spatial domains further highlights the robustness and versatility of SpaBatch in handling complex and heterogeneous spatial transcriptomics data, enabling a comprehensive understanding of the tissue organization within the developing embryo.

We further explored the relationships between different spatial domains using a spatial domain correlation heatmap and observed a significant correlation between domain 1 and domain 13 (Figure 6B,C). This finding drew our attention, as the close connection between these two domains may reveal their potential functional association during mouse embryonic development. In subsequent analysis, we compared these two domains with manually annotated regions in the embryo and found that domain 1 and domain 13 strongly overlapped with the annotated heart and cavity regions. The marker gene *Sh3bgr* exhibited significant and specific expression in this region across all fourslices, further supporting their correlation (Figure 6E). *Sh3bgr* has been reported to be associated with myocardial development.^[^ ^48^ ^]^ Moreover, the associated regions also include domain 11 and domain 12. From the correlation heatmap, it was observed that these two spatial domains highly overlap with the manually annotated brain and spinal cord (Figure S12A, Supporting Information). We performed GO enrichment analysis on the two spatial domains. The spatial domain highly associated with the brain primarily includes neuron development, synapse organization, axon guidance, and other functions, which are closely related to the complex neural network development and functional regulation in brain tissues. In the spatial domain highly associated with the spinal cord, similar neuron function‐related terms also appeared, such as neuron migration, axon repair, and motor neuron regulation. There is significant functional overlap in the GO enrichment results of these two spatial domains, demonstrating highly consistent functional directions (Figure S12D, Supporting Information). This overlap and similarity in terms validate the high correlation between domain 11 and domain 12 in the correlation heatmap.

### 3.4 SpaBatch Identifies the Development of the Human Heart

**Figure 7:**
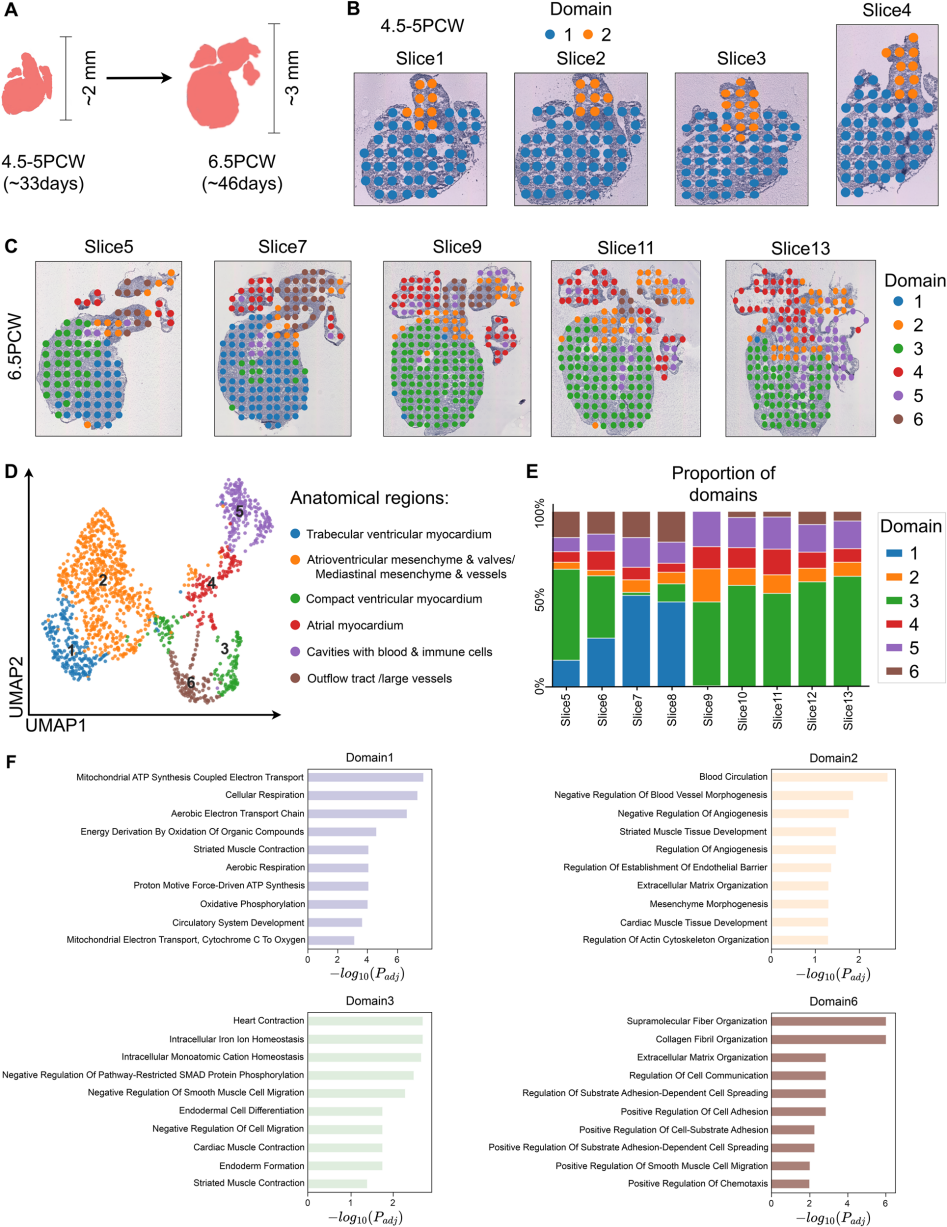
SpaBatch identifies the development of the human heart. A) The development of the human heart from 4.5‐5 PCW to 6.5 PCW. B) SpaBatch spatial domain identification in the human heart at 4.5‐5 PCW. C) SpaBatch spatial domain identification in the human heart at 6.5 PCW. D) UMAP visualization of the human heart at 6.5 PCW and its corresponding anatomical regions. E) SpaBatch spatial domain proportion stacked bar plot in the human heart at 6.5 PCW. F) GO enrichment analysis of spatial domains in the 6.5 PCW heart.

We applied SpaBatch to the human heart ST dataset, which includes two sets of slices collected from human embryonic hearts at 4.5‐5 and 6.5 post‐conception weeks (PCW) (**Figure** **7**A). This data was used to explore SpaBatch's ability to identify the dynamic changes in tissue structures across slices with developmental processes.

At 4.5‐5 PCW, SpaBatch identified two distinct spatial domains, which further developed at 6.5 PCW (Figure 7B). Next, we focused on the human heart at 6.5 PCW, where SpaBatch identified six spatial domains across nine sections (Figure 7C and Figure S13, Supporting Information). Based on the anatomical region annotations provided by a previous report,^[^ ^26^ ^]^ we mapped the identified spatial domains to their corresponding anatomical regions (Figure 7D). For example, spatial domains 1, 3, and 4 corresponded to the trabecular ventricular myocardium, compact ventricular myocardium, and atrial myocardium, respectively. These were all important components of the cardiac muscle. The trabecular myocardium and compact myocardium were typically present in the ventricles. The compact myocardium provided strong contractile force, while the trabecular myocardium was responsible for structural support and local blood flow. The atrial myocardium supported the overall blood flow of the heart and coordinated the contraction between the atria and ventricles.^[^ ^49^ ^]^ By visualizing the proportion of spatial domains identified by SpaBatch, the dynamic changes in the 6.5 PCW human heart tissue could be intuitively observed (Figure 7E).

The human heart data did not have manual annotations as ground truth. For the identified spatial domains, we performed GO enrichment analysis to characterize their functions.^[^ ^50^ ^]^ Domain 1 corresponds to the trabecular ventricular myocardium region, and the top enriched GO terms are primarily related to cellular respiration. The GO terms enriched in domain 2 involve blood circulation, vasculature morphogenesis, angiogenesis, etc., which align with its corresponding anatomical region. Domain 3 corresponds to the compact ventricular myocardium region and is enriched with processes related to cardiac development and heart contraction. The terms enriched in domain 6 are associated with extracellular matrix (ECM) tissue and are related to the integrity and stability of the cardiovascular system (Figure 7F).

### 3.5 SpaBatch Leverages Limited Annotations for Accurate Spatial Domain Detection in the HER2‐positive Breast Cancer Dataset

In addition to normal tissue, we applied SpaBatch to the HER2‐positive breast cancer dataset to evaluate its ability to make biological discoveries in abnormal tissue slices. The dataset consists of tumor samples from 8 different patients, represented as groups A‐H. Groups A‐D each contain 6 slices, while groups E‐H each contain 3 slices. We performed separate analyses for each of the A‐H groups.^[^ ^27^, ^51^ ^]^

**Figure 8:**
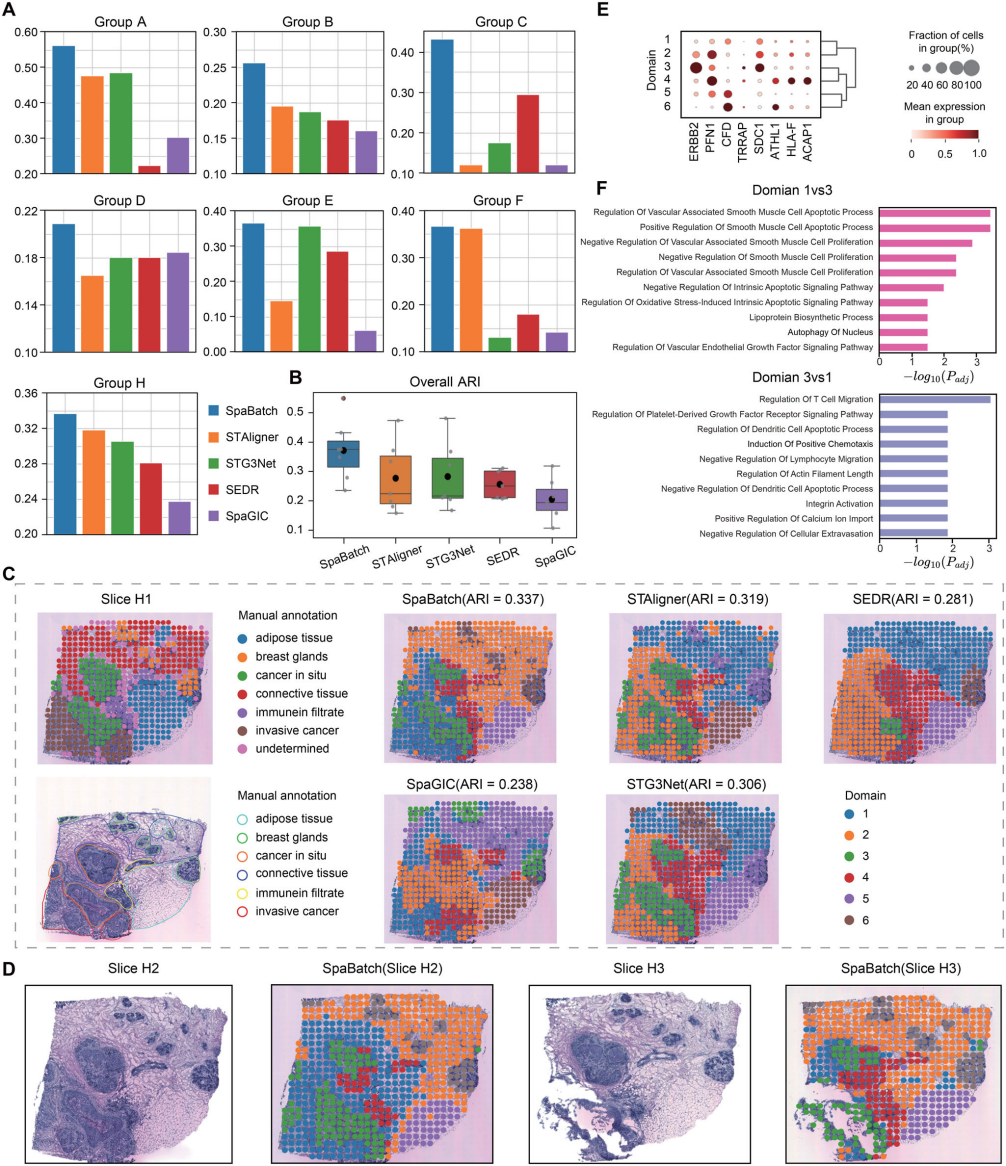
Leveraging limited annotations for accurate spatial domain detection in the HER2‐positive breast cancer dataset. A) The ARI calculated for the first slice of group A‐H in the HER2‐positive breast cancer dataset using SpaBatch compared with other methods. B) The overall boxplot of ARI values for each group. C) Manual annotation of slice H1 (left), and the results of spatial domain identification on slice H1 using SpaBatch and other methods (right). D) The spatial domains of slices H2 and H3 predicted by SpaBatch through joint training on the H group data. E) Bubble plot of the differential analysis between cancer regions and other regions. F) GO enrichment analysis results of the in situ cancer and invasive cancer.

In groups A‐H, only the first slice was annotated by a pathologist. We conducted experiments with SpaBatch, STAligner, STG3Net, SEDR, and SpaGIC on this dataset, and calculated the ARI on the first slice (Figure S14, Supporting Information). As shown in **Figure** **8**A, SpaBatch outperformed other methods in 3D spatial clustering performance across all slices in all groups. In terms of overall clustering performance, SpaBatch also achieved the highest median (ARI = 0.367) and mean (ARI = 0.362), outperforming the second‐place STAligner with a median (ARI = 0.196) and mean (ARI = 0.255), showing a significant improvement (Figure 8B). We then focused on the H group data, which contains most diverse spatial domains. Neither SpaGIC nor SEDR were able to identify the cancer in situ region. STG3Net failed to correctly identify the breast glands. SpaBatch and STAligner performed the best on slice H1, correctly identifying continuous regions such as cancer in situ and adipose tissue, as well as discrete regions such as breast glands. SpaBatch outperformed STAligner in spatial domain range and boundary identification, achieving a higher ARI (Figure 8C). Interestingly, in the joint multi‐slice training of Group H, SpaBatch achieves 3D spatial domain identification across slices using manual annotations from only slice H1. As shown in the annotations of H1, the spatial domain 1 identified by SpaBatch aligns closely with the region of invasive cancer, while domain 3 nearly overlaps with the cancer in situ. Furthermore, visualizations of slices H2 and H3 reveal that the predicted domains 1 and 3 consistently correspond to the dark lesion areas in the H&E images, exhibiting spatial patterns similar to those in H1. These results demonstrate that SpaBatch effectively leverages limited annotation data to perform semi‐supervised learning, enabling accurate cross‐slice spatial domain identification without requiring full annotations for every slice (Figure 8D). In practical research and clinical applications, ST data with complete annotations is often scarce. SpaBatch can achieve accurate 3D spatial domain identification with only partial annotations, thereby enabling scalable and reliable spatial analysis in real scenarios.

We performed differential analysis between cancer regions and other regions. As shown in Figure 8E, SpaBatch indeed identified that the cancer in situ region recovered from domain 3 highly expresses the breast cancer marker *ERBB2* compared to other regions.^[^ ^22^ ^]^ Next, we investigated the heterogeneity of the cancer regions. Spatial domain 3 exhibited various immune response‐related GO terms, including signaling pathways for B cells and T cells, as well as the regulation of respiratory burst, suggesting a strong immune response in the in situ cancer region. On the other hand, spatial domain 1 was enriched in pathways that inhibit cell apoptosis, which may indicate the presence of more anti‐apoptotic mechanisms in invasive cancer, helping tumor cells evade apoptosis.^[^ ^52^ ^]^ The differences in enriched biological processes highlight the distinctions between these two regions, emphasizing their respective immune/stromal microenvironments (Figure 8F).

### 3.6 SpaBatch Accurately Maps Spatial Domains in the Preoptic Area of the Mouse Hypothalamus From the MERFISH Platform and Enables 3D Reconstruction

To evaluate the broad applicability of SpaBatch across different sequencing technologies, we applied it to a dataset generated using the imaging‐based MERFISH technique, which comprises consecutive coronal slices of the mouse hypothalamus. Specifically, the dataset includes three sequential sections: hypothalamus 0.09, 0.14, and 0.19, with manual annotations provided for each slice. A total of eight anatomical regions were identified, including the bed nucleus of the stria terminalis (BST), medial preoptic area (MPA), medial preoptic nucleus (MPN), paraventricular nucleus of the hypothalamus (PV), paraventricular hypothalamic nucleus (PVH), paraventricular nucleus of the thalamus (PVT), third ventricle (V3), and fornix (fx).^[^ ^29^ ^]^

**Figure 9:**
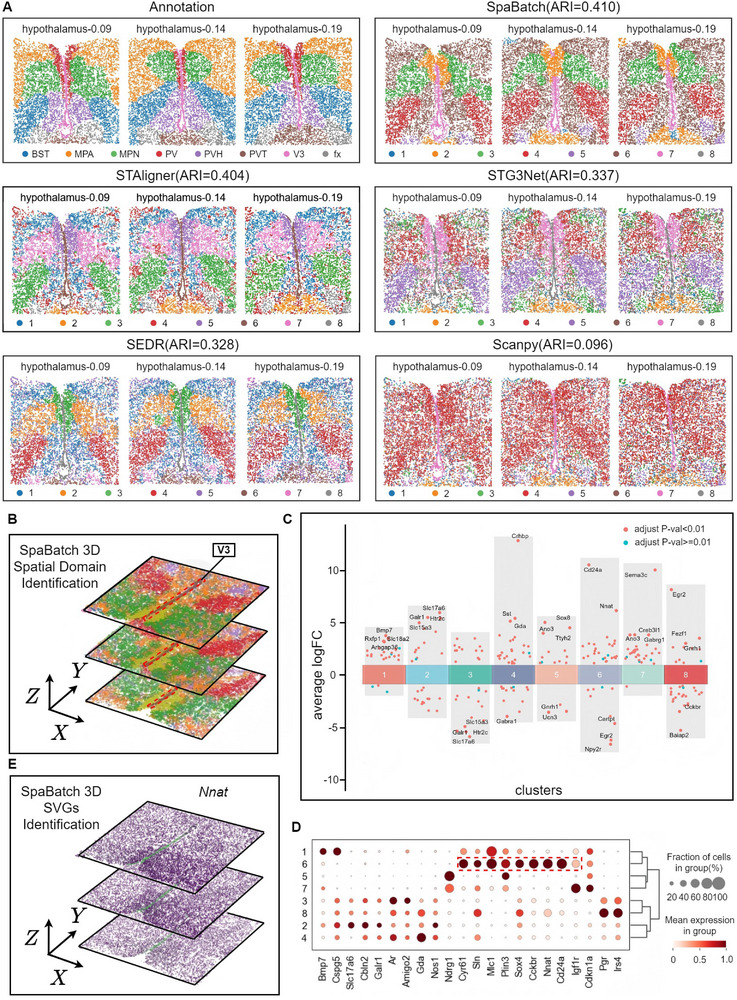
SpaBatch accurately maps spatial domains in the preoptic area of the mouse hypothalamus from the MERFISH platform and enables 3D reconstruction. A) Spatial domains identified by SpaBatch and other methods on three consecutive slices of the mouse hypothalamic preoptic area profiled by the MERFISH platform. B) 3D spatial domains reconstructed by SpaBatch after aligning the coordinates of three consecutive slices. C) Differential gene expression analysis showing up‐ and down‐regulated genes across all eight clusters. An adjusted p value < 0.01 is indicated in red, while an adjusted p value R 0.01 is indicated in teal. D) Bubble plot of differential analysis across eight clusters. E) SpaBatch achieved the 3D spatial reconstruction of the spatially variable gene (SVG) *Nnat* in the V3 region (Domain 6).

We compared SpaBatch with four methods, among which Scanpy^[^ ^30^ ^]^ represents a conventional approach. Scanpy applies principal component analysis (PCA) to reduce the dimensionality of gene expression data, followed by clustering, without involving explicit nonlinear modeling or batch effect correction. When visualizing the clustering results mapped back to the original tissue sections, Scanpy failed to capture the spatial patterns corresponding to distinct anatomical structures in the mouse hypothalamus. Compared with other deep learning‐based models, the clustering boundaries in Scanpy appeared more dispersed, and the resulting clusters exhibited lower compactness and spatial consistency. This highlights the limitations of traditional methods in handling complex spatial transcriptomic patterns. In contrast, SpaBatch outperformed other deep learning methods in identifying most spatial domains and showed greater agreement with histological annotations. It achieved the highest mean ARI score of 0.410 across the three consecutive coronal sections, and also yielded the best performance in terms of ACC and V‐measure (**Figure** **9**A; Figure S15A, Supporting Information). SpaBatch accurately identified major regions such as BST, V3, and MPA. Most notably, it produced more compact clusters with smoother boundaries. By comparison, other methods frequently misassigned spots within a given spatial domain to neighboring regions. Furthermore, SpaBatch also demonstrated superior performance in batch effect removal metrics (Figure S15A, Supporting Information). To evaluate the performance of non‐Euclidean distance metrics compared to the Euclidean distance within the SpaBatch framework on the MERFISH dataset, we incorporated adjacency matrices constructed using Euclidean, Manhattan, Chebyshev, Cosine, and Hamming distances. Among these, the graph constructed with the Euclidean distance achieved the best performance, followed by the Chebyshev distance (Figure S15B, Supporting Information). It is worth noting that we introduced a center‐alignment method to adjust the spatial coordinates of the three sections, which enabled us to align and visualize the 2D spatial domain identification results in 3D space (Figure 9B).

The third ventricle (V3) is a midline structure in the brain filled with cerebrospinal fluid, located within the diencephalon, primarily between the thalamus and hypothalamus. It extends along the midsagittal plane of the brain, connecting to the lateral ventricles anteriorly and to the fourth ventricle posteriorly via the cerebral aqueduct. SpaBatch accurately identified this region, which exhibited clear boundaries from surrounding areas. We further performed differential analysis across multiple spatial domains using volcano plots, where *Cd24a* and *Nnat* were identified as significant marker genes for spatial domain 6 (V3 region) (Figure 9C; Figure S15B, Supporting Information). These marker genes were further validated using bubble plots to assess their expression proportions and average expression levels across spots (Figure 9D). The results confirmed that *Cd24a* and *Nnat* were highly expressed in the V3 region but showed low expression in other spatial domains. We visualized marker genes such as *Cd24a* and *Nnat*. With the help of our center‐alignment algorithm, which adjusts the spatial coordinates of the three sections, these genes were also visualized in 3D space (Figure 9E; Figure S15C, Supporting Information).

In addition, we performed GO enrichment analysis^[^ ^50^ ^]^ on the identified V3 region (Figure S15D, Supporting Information). The results revealed several terms associated with neurodevelopment and differentiation, including noradrenergic neuron differentiation (GO:0003357) and neuroepithelial cell differentiation (GO:0060563). *SOX4* was identified as a key transcription factor involved in regulating the differentiation of neural progenitor cells and influencing the development of both the central and peripheral nervous systems. Another enriched term was negative regulation of calcium ion import (GO:0090281), which is related to calcium signaling, a fundamental mechanism underlying neuronal excitability and synaptic plasticity. The gene *SLN* may indirectly regulate neural function through this pathway. Consistent with a previous study,^[^ ^53^ ^]^ we also observed significant enrichment in pathways related to glucose and monosaccharide response (GO:0050796 and GO:0032024). This may be attributed to the fact that the V3 region is primarily composed of ependymal cells, suggesting a potential role in carbohydrate transport.^[^ ^54^ ^]^ These pathways were enriched due to the expression of the gene *Nnat*.

SpaBatch performed well on the MERFISH dataset, further validating its broad applicability and robustness in 3D spatial domain identification across different platforms, sequencing technologies, species, and dataset sizes. Although imaging‐based and sequencing‐based ST technologies exhibit distinct spatial distributions of spots, SpaBatch constructs graphs based on Euclidean distances and employs a graph neural network to aggregate features within local neighborhoods. As a result, its clustering performance remains unaffected by whether the spot distribution is regular or irregular, demonstrating strong adaptability and stability.

### 3.7 SpaBatch Accurately Identifies Spatial Domains on Partially Overlapping Slices

**Figure 10:**
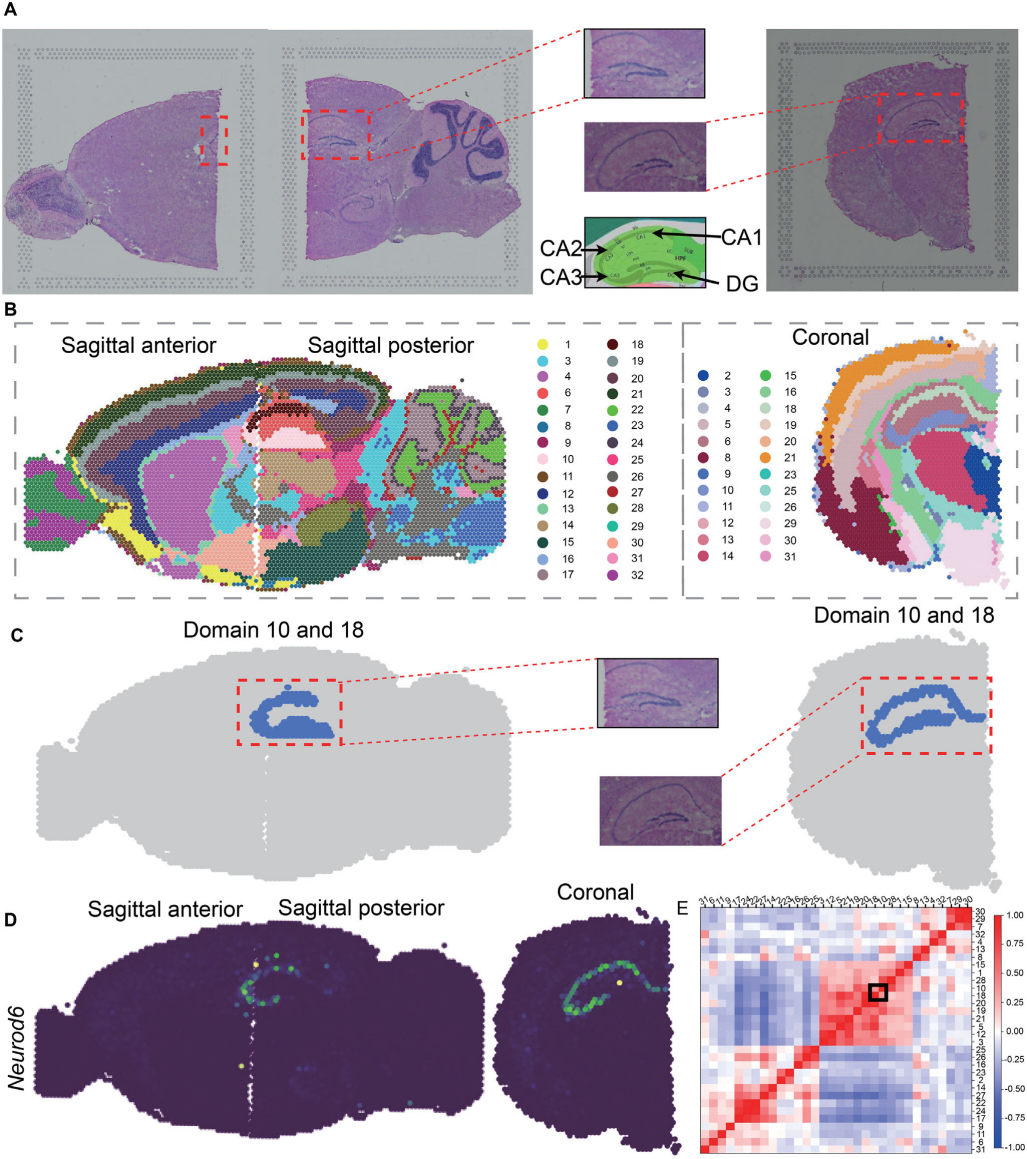
SpaBatch accurately identifies spatial domains on partially overlapping slices. A) H&E images of sagittal mouse anterior‐posterior brain sections (from Section 1), a coronal section, the mouse cortex reference atlas from the Allen Brain Atlas, along with their corresponding specific spatial subdomains (hippocampus). B) SpaBatch spatial domain identification results on sagittal anterior‐posterior and coronal sections of the mouse brain. C) Spatial subdomains within the hippocampus identified by SpaBatch on sagittal anterior‐posterior and coronal sections of the mouse brain. D) The spatial marker gene *Neurod6* in sagittal anterior‐posterior and coronal sections of the mouse brain shows concentrated expression in the hippocampal region. E) SpaBatch identifies correlation heatmaps across all spatial domains.

To evaluate the robustness of SpaBatch in handling partially overlapping slices, we designed an experiment to assess its performance in integrating morphologically distinct sections. Building upon the previously used sagittal anterior and posterior mouse brain slices, we introduced an additional coronal slice that differs in structural orientation and morphology, and performed joint integration analysis with the two sagittal slices (**Figure** **10**A).

Despite the significant morphological and spatial directional differences between the coronal and sagittal sections, all three slices originate from the same tissue (mouse brain), thus sharing consistent gene expression patterns. SpaBatch is capable of effectively capturing this cross‐slice expression consistency, enabling accurate integration even across structurally divergent slices.

In the analysis using only sagittal slices, SpaBatch successfully identified anatomically distinct regions, such as the olfactory bulb in the anterior brain and the cerebellar cortex in the posterior brain. More importantly, SpaBatch also demonstrated strong performance in identifying fine‐grained structures spanning both anterior and posterior regions, including multiple cortical regions and the hippocampus (Figure 4).

After incorporating the coronal slice with low spatial overlap,SpaBatch still performed robustly. The model accurately delineated the hippocampal region within the coronal slice, which closely aligned with the corresponding region in the histological (H&E stained) image (Figure 10B,C). Although there was a slight decrease in hippocampal identification accuracy compared to using only sagittal slices, possibly due to added noise during cross‐slice integration, the model maintained strong spatial consistency overall. Additionally, SpaBatch successfully identified cortical regions in the coronal slice that shared similar laminar structures with those found in the sagittal slices, located just above the hippocampus. Visualization of the domain marker gene *Neurod6*, originally identified in the sagittal slices, further confirmed its role as a hippocampus‐specific marker in the coronal slice (Figure 10D). The spatial correlation matrix also supports the notion that clusters 10 and 18 jointly constitute the hippocampal structure, as they appear adjacent and highly correlated in the heatmap (Figure 10E).

We would like to emphasize that partially overlapping slices pose a significant challenge for cross‐slice alignment due to limited shared spatial regions and inconsistent coordinate systems. Nevertheless, SpaBatch is still able to achieve effective multi‐slice integration under such conditions, which is technically explainable. SpaBatch does not rely on explicit inter‐slice adjacency or registration information. Instead, it enables information sharing by jointly optimizing the gene expression representations across all slices using shared trainable parameters within the model. Although no direct inter‐slice connections are constructed in the graph structure, the block‐diagonal adjacency matrix allows the GNN to aggregate spatial information within each individual slice, while the shared encoder and decoder learn a unified latent space that captures gene expression patterns across slices.

This architecture enables indirect cross‐slice information sharing, allowing SpaBatch to learn consistent and robust spatial representations even in scenarios with partial overlaps or morphological discrepancies. Therefore, the effectiveness of SpaBatch in such cases is supported not only by empirical results but also by its model design principles.

### 3.8 SpaBatch Denoises Gene Expression and Enhances the Expression Patterns of Spatial Marker Genes

In our previous analysis, SpaBatch identified spatial marker genes corresponding to different cortical layers, such as *AQP4* (layer 1), *CARTPT* (layer 2), *ENC1* (layer 3), *PCP4* (layer 4), *TMSB10* (layers 4 and 5), and *MBP* (white matter) (Figure 2).

Here, we further investigate these spatial marker genes identified by SpaBatch. Using four sections from Donor 3 as an example, the spatial domains corresponding to cortical layers 1–6 and white matter identified by SpaBatch are consistent with the classical anatomical lamination of the cerebral cortex (Figure S16A, Supporting Information). By visualizing the spatial distribution of the raw (top) and SpaBatch‐denoised (bottom) expressions of *PCP4*, *TMSB10*, and *MBP*, we observed that these spatial marker genes are strongly expressed in specific cortical layers, while exhibiting low expression in others. The denoised expression signal appears more concentrated and clearer, demonstrating higher spatial specificity, substantially reduced background noise, and enhanced distinguishability of spatial expression patterns for marker genes (Figure S16B, Supporting Information). Violin plots of raw and denoised expression across different layers and corresponding marker genes show that the median expression levels of *TMSB10* and other spatial marker genes (e.g., *PCP4*, *ENC1*) are noticeably increased after denoising, with more concentrated distributions. This reflects strengthened expression signals and suppressed noise (Figure S16C, Supporting Information). Red dashed boxes highlight layers and genes with significant denoising effects, further validating that SpaBatch effectively enhances the biological markers of spatial domains through denoising.

In conclusion, SpaBatch not only accurately identifies spatial domains but also significantly improves the spatial expression patterns of key marker genes through denoising, facilitating downstream interpretation and analysis of spatial biological functions.

### 3.9 Ablation Study and Mask Rate Selection

**Figure 11:**
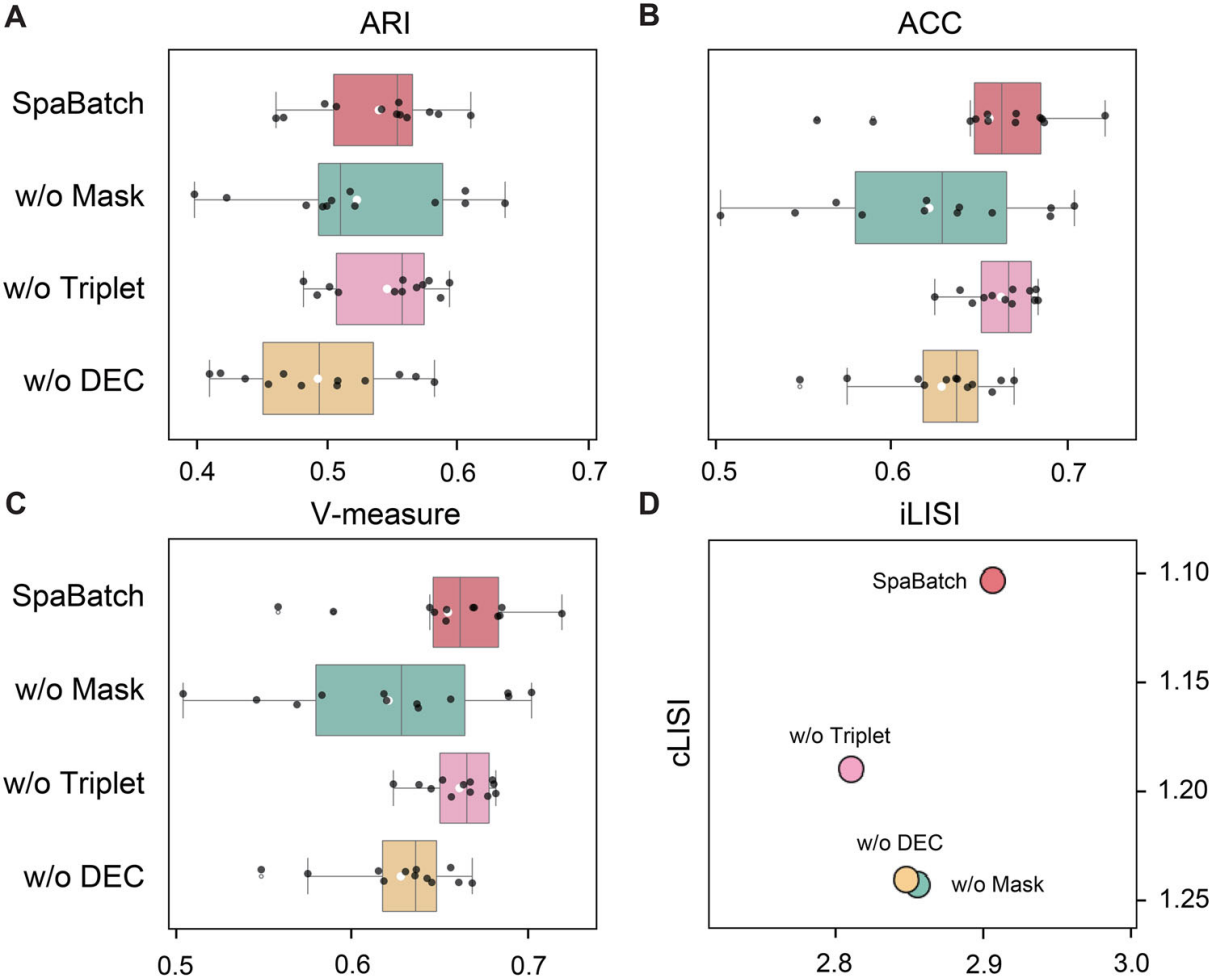
Comparison results of SpaBatch and its ablated versions (without Mask, without Triplet, and without DEC) on the DLPFC dataset in terms of clustering metrics (ARI, ACC, V‐measure) and batch correction metrics (iLISI, cLISI).

To evaluate the contribution of each key component in SpaBatch to the overall performance, we conducted ablation experiments by individually removing three important modules from the model: the Mask mechanism for data augmentation, the DEC module for compact clustering, and the triplet loss for batch effect correction. **Figure** **11** presents the performance of the full model and its ablated versions on the DLPFC dataset, reporting three clustering evaluation metrics (ARI, ACC, and V‐measure) as well as two batch correction metrics (iLISI and cLISI).

The results show that the full SpaBatch model achieves the best or near‐best performance across all clustering metrics, indicating that the three modules work synergistically to enhance clustering quality. In particular, the removal of the DEC module (w/o DEC) leads to a significant drop in clustering performance, demonstrating its critical role in guiding the formation of compact latent clustering structures. Similarly, removing the Mask and Triplet modules also results in decreased clustering performance, suggesting that both components contribute substantially to improving clustering effectiveness.

In terms of batch correction, the iLISI and cLISI results indicate that SpaBatch, with the inclusion of the triplet loss, achieves the best balance between data integration and preservation of biological structure. Overall, the incorporation of the Mask, DEC, and Triplet modules plays an indispensable role in enhancing model performance, validating the rationality and effectiveness of the SpaBatch design.

**Figure 12:**
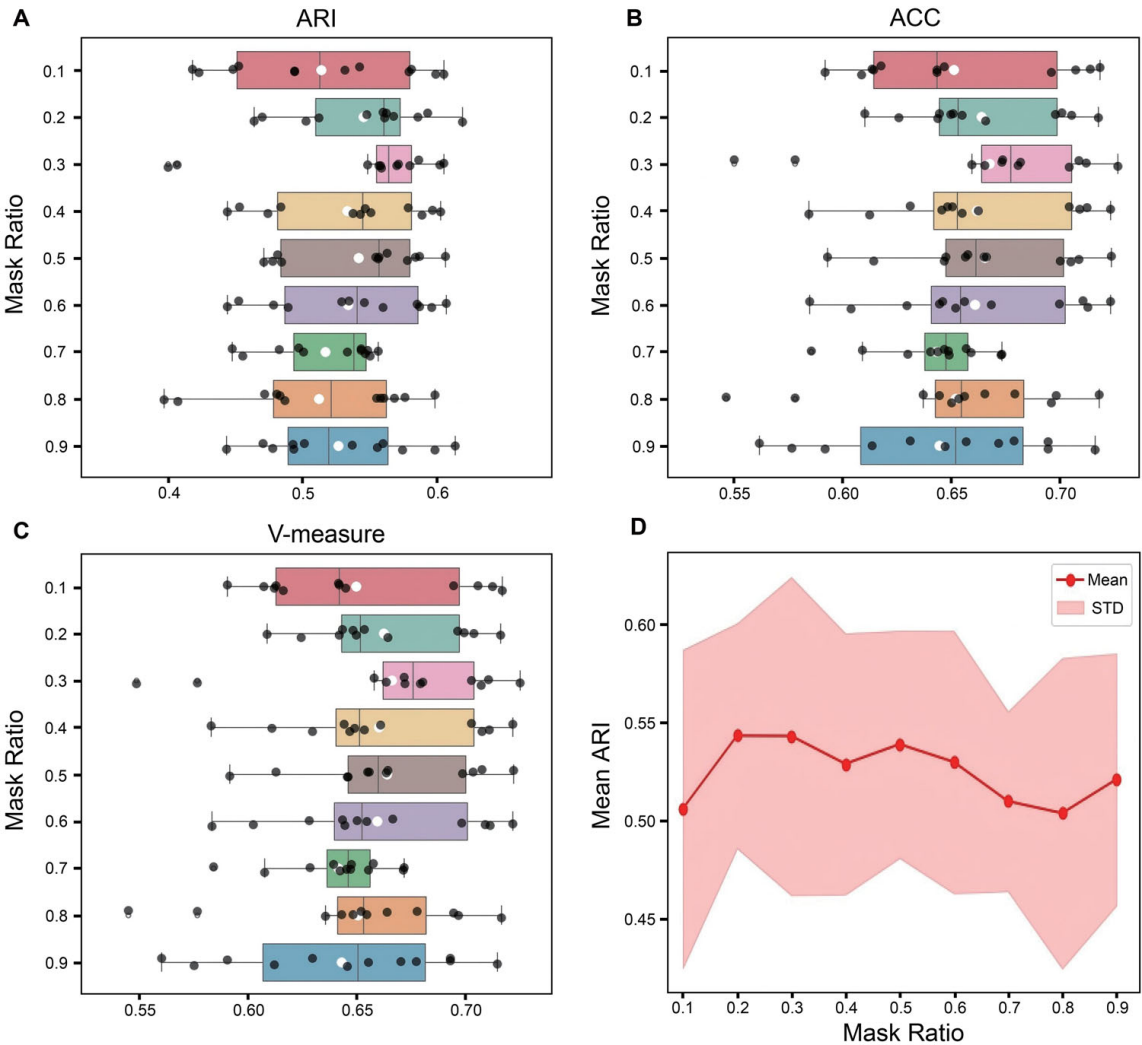
Impact of mask ratio on spatial domain identification performance on the DLPFC dataset. The mask ratio was set from 0.1 to 0.9 with an interval of 0.1, and A) ARI, B) ACC, and C) V‐measure were calculated. D) In the line plot (bottom‐left), the red line indicates the average ARI, and the red shaded area represents the standard deviation (STD).

To investigate the effect of the mask ratio on model performance and to justify the choice of 0.2 as the default setting, we conducted experiments with mask ratios ranging from 0.1 to 0.9 in increments of 0.1. The results showed that as the mask ratio increased, model performance first improved and then declined, with 0.2 and 0.3 achieving the best results. Although the setting with a mask ratio of 0.3 exhibited a higher median and a more compact distribution in the box plot, it also had more outliers, leading to a slightly lower overall mean compared to the default value of 0.2 and potentially introducing instability in downstream analyses. Therefore, we selected 0.2 as the default setting, as it demonstrated better stability and higher average performance. (**Figure** **12**).

To validate whether the outliers observed at a mask ratio of 0.3 would affect downstream analyses, we conducted additional experiments on a sagittal mouse brain dataset, which features more complex tissue structures (Figure S17, Supporting Information). At a mask ratio of 0.2, SpaBatch most accurately identified continuous spatial domains across adjacent slices, particularly in regions such as the cortex, hippocampus, thalamus, and hypothalamus. Notably, only under this setting was the complete trisynaptic circuit of the hippocampus, including CA1, CA2, CA3, and the dentate gyrus (DG), clearly delineated. When the mask ratio exceeded 0.6, we observed discontinuities in cortical regions, while extreme values such as 0.1 and 0.9 impaired the depiction of the cerebellum. Nevertheless, SpaBatch consistently captured fine anatomical structures under most settings, demonstrating its robustness and supporting the choice of 0.2 as the default mask ratio.

### 3.10 Effect of Different Graph Convolution Operators

GCNConv^[^ ^33^ ^]^ features a simple structure and high computational efficiency, enabling it to stably capture local expression patterns from spatial adjacency graphs. It strikes a good balance between model stability, neighborhood structure modeling, and computational cost, making it particularly suitable for large‐scale graph‐structured ST data. Its effectiveness has been demonstrated in various spatial transcriptomics tasks.^[^ ^7^, ^10^, ^23^ ^]^

To validate the rationale for using GCNConv as the graph convolution layer in SpaBatch, we conducted a comparative experiment. Specifically, we replaced GCNConv with GATConv, GraphConv, SAGEConv, and FeastConv, respectively, and integrated each into the backbone of SpaBatch to evaluate their performance in multi‐slice spatial domain identification. The experiment was conducted on the DLPFC dataset, where 12 tissue slices were grouped into three subsets based on donor: Donor 1 (151507–151510), Donor 2 (151669–151672), and Donor 3 (151673–151676).

We computed several clustering evaluation metrics under each graph convolution configuration, including ARI, ACC, V‐measure, and LISI (iLISI and cLISI), and visualized the results using boxplots to assess performance stability (Figure S18A, Supporting Information). As expected, GCNConv achieved the best overall performance, with the highest means and medians across all metrics, and the smallest variance, demonstrating strong robustness. Other convolution layers, such as SAGEConv and FeastConv, also performed reasonably well, with ARI medians around 0.55, suggesting that SpaBatch is relatively insensitive to the choice of convolution type. LISI analysis further confirmed that GCNConv was the most effective in mitigating batch effects.

We further visualized and presented the clustering results on Donor 3 (Figure S18B, Supporting Information). GCNConv achieved the highest ARI score (0.613), followed by FeastConv (0.598) and SAGEConv (0.588), while GATConv performed the worst (0.448). This discrepancy may be attributed to the attention mechanism of GATConv being more sensitive to sparsity or noise in gene expression data. Compared to manual annotations, all convolution layers except GCNConv failed to fully capture certain cortical layers. For example, although SAGEConv achieved results relatively close to GCNConv, its recognition of Layer_6 was incomplete, indicating a performance gap. GCNConv also showed strong results in the other two Donor subsets (Figure S19, Supporting Information).

We also examined the UMAP visualization of the learned embeddings to assess whether the clustering structure aligned with the biologically expected laminar organization (Figure S18C, Supporting Information). Overall, GCNConv's neighborhood aggregation strategy demonstrated superior robustness on this dataset, whereas GATConv may require further parameter tuning to enhance its performance.

It is worth noting that no architectural or hyperparameter tuning was performed for the different convolution layers in this experiment, which may introduce some unfairness, particularly for GATConv. GATConv incorporates a learnable attention mechanism that assigns different weights to neighboring nodes, enabling more flexible and adaptive feature aggregation. In theory, this mechanism allows the model to dynamically focus on the most informative neighbors, offering greater representational power and adaptability. Although GATConv underperformed in this study, possibly due to its sensitivity to noise or suboptimal default settings, we believe it still holds great potential for ST. Future work could explore enhancements such as incorporating edge weights, spatial location awareness, or hierarchical attention mechanisms to better leverage its strengths in modeling complex tissue architecture.

### 3.11 Selection of the Number of Positive Samples in the Readout‐Based Triplet Aggregation Strategy

We introduced a readout‐based triplet aggregation strategy to address the potential errors that may arise when selecting positive samples at the individual spot level. By aggregating features from positive samples prior to contrastive comparison, the model can form a more robust and stable representation of the target structure, which helps reduce errors caused by incorrectly selected individual positives.

We conducted an experiment to analyze how varying the number of aggregated positive samples in the readout‐based triplet strategy affects model performance, and to demonstrate its advantage over both removing the module and using point‐wise contrastive learning. Specifically, we observed the performance of SpaBatch on the anterior and posterior mouse brain sagittal sections dataset by either removing the module (Num of Aggregate Positive = 0) or gradually increasing the number of aggregated positive samples.

As shown in the previous ablation study on the DLPFC dataset, removing this module led to a noticeable drop in all three clustering metrics (ARI, ACC, and V‐measure) relative to the full model. The performance of LISI, including iLISI and cLISI, which evaluates batch effect correct, also decreased significantly after removing the module (Figure 11). We observed that as the number of aggregated positive samples increased, the ARI score first improved, then declined, and eventually stabilized (Figure S20A, Supporting Information). Visualization results further revealed changes in the recognition of fine‐grained structures. Without this module, the compactness of clusters and the continuity across spatial domains were significantly compromised. For example, the caudate‐putamen (CP) region included many misassigned spots that did not belong to the domain, substantially lowering its identification accuracy. In addition, cross‐slice cortical alignment became less smooth.

After introducing the module, these problems were significantly mitigated. However, when the number of aggregated positives was set to one, the hippocampus was not fully recovered, although larger spatial domains were identified relatively accurately. At an aggregation number of two, the model achieved optimal performance, successfully identifying both fine structures and cross‐slice regions such as the hippocampus. When the number was increased to four, five, or six, performance remained stable and largely consistent with the results at two. However, the ventral part of the hippocampus was not recognized, making the performance slightly inferior (Figure S20B– D, Supporting Information). As the number of positive samples continued to increase, performance started to decline. We speculate that excessive aggregation of positive samples may introduce boundary noise, which makes it harder for the model to accurately distinguish between adjacent but biologically distinct spatial domains.

In summary, choosing an appropriate number of positive samples for aggregation is critical for model performance and accurate spatial domain identification. Moderate aggregation strikes a balance between stability and detail capture, whereas over‐aggregation may lead to feature mixing and degraded performance.

## 4 Conclusion

The biological activities of organisms and tissues are inherently 3D. Limiting spatial transcriptomics to 2D analysis hinders the exploration of complex tissue architectures. Compared with single‐slice spatial domain identification, multi‐slice joint analysis presents greater technical and biological complexity and challenges. Single‐slice analysis focuses solely on the spatial structure within an individual tissue section and explores local gene expression patterns, making it suitable for preliminary studies of spatial heterogeneity within tissues. In contrast, multi‐slice integration analyzes data from multiple consecutive or non‐consecutive sections. It not only identifies spatial structures within each slice but also aligns and integrates spatial information across slices to construct a consistent spatial expression map. This process must address challenges such as batch effects and spatial misalignment between slices and requires algorithms with strong alignment capabilities and cross‐slice feature extraction.

With the rapid advancement of sequencing technologies, an increasing number of high‐quality spatial transcriptomics (ST) datasets are being generated. This creates a pressing need for methods capable of integrating multiple slices from the same individual, slices of the same tissue obtained from different platforms, and consecutive slices across developmental stages. These datasets often vary in experimental protocols, sequencing platforms, dimensions, shapes, and orientations. To address these challenges, we propose SpaBatch, a novel framework designed for multi‐slice ST data integration and 3D spatial domain identification. In the data preprocessing stage, SpaBatch applies a mask‐based data augmentation strategy to the gene expression data to improve robustness to sparsity and noise. During training, SpaBatch adopts a pretraining‐and‐finetuning paradigm: the backbone VGAE network is first pretrained to capture initial spatial structure and transcriptional features, followed by finetuning with a deep embedded clustering (DEC) module to enhance cluster compactness. Additionally, a triplet contrastive loss based on a readout strategy is introduced to explicitly correct for batch effects.

Compared to classical spatial domain identification methods such as STAGATE and SEDR, which first learn low‐dimensional embeddings of ST data using graph convolutional networks and then correct batch effects using Harmony in a step‐by‐step manner, SpaBatch adopts an end‐to‐end joint learning framework that directly maps the raw input to the final output. All intermediate steps are integrated into a unified architecture and handled automatically by the model, eliminating the need for post hoc batch correction (Supplementary Figure S21). During training, SpaBatch incorporates multiple functional modules and simultaneously performs embedding learning, batch effect correction, and clustering optimization. These tasks are jointly optimized within a single model through parameter sharing and collaborative learning, which facilitates consistent cross‐slice representations and accurate spatial domain identification, while preserving the underlying spatial structure.

SpaBatch yields many biologically meaningful discoveries across different datasets. On the DLPFC dataset, SpaBatch achieves 3D spatial domain identification results most consistent with manual annotations, accurately delineating layers 1 to 6 and the white matter (WM), with well‐mixed clusters within the same cortical layer. UMAP visualization further confirms SpaBatch's effectiveness in batch correction, demonstrating excellent inter‐slice mixing. On the mouse sagittal brain dataset, SpaBatch accurately identifies spatial domains specific to the forebrain or hindbrain—such as the olfactory bulb and cerebellar cortex, respectively. More importantly, it also excels at recognizing shared domains spanning the forebrain and hindbrain, including the continuous identification of dorsal and ventral hippocampal regions, with remarkably consistent cortical layers. In the mouse coronal brain dataset, SpaBatch performs 3D spatial domain identification to manually map each slice onto the Allen Brain Atlas mouse cortex reference, showing tissue composition ratios and anatomical structures, thereby constructing a comprehensive atlas of the mouse coronal brain. On a mouse embryo dataset, SpaBatch accurately identifies 3D spatial domains and uses heatmaps to validate the relevant spatial domains, achieving alignment with specific anatomical structures by stacking 3D predictions. SpaBatch also accurately captures the dynamic changes of the human embryonic heart. In the HER2‐positive breast cancer dataset, SpaBatch employs semi‐supervised learning by leveraging limited manual annotations from the first slice to guide the training process, achieving accurate spatial domain identification in the other slices, and precisely labeling regions of carcinoma in situ, invasive carcinoma and other areas. Across various spatial transcriptomics datasets, SpaBatch demonstrates superior performance and broad applicability—not only achieving high‐precision 3D spatial domain identification but also uncovering biologically meaningful structures. In mouse hypothalamic slices sequenced using the MERFISH technique, SpaBatch accurately identifies the slender and fine anatomical structure of the third ventricle (V3), with spatial domains and spatial marker genes reconstructed in 3D using a center‐alignment algorithm.

In conclusion, SpaBatch is a universal and efficient framework for 3D spatial domain identification that effectively addresses the heterogeneity in source, scale, orientation, and batch effects inherent in multi‐slice ST datasets. By integrating mask‐based augmentation, pretraining‐finetuning strategy, deep clustering, and triplet contrastive learning, SpaBatch significantly improves clustering accuracy and consistency while enabling cross‐slice alignment and batch transferability. In real datasets across various scenarios, SpaBatch reconstructs complex tissue architectures, uncovers key biological features and developmental dynamics, and demonstrates great potential in structural analysis, developmental biology, and disease characterization. SpaBatch provides robust technical support for advancing spatial transcriptomics from 2D to 3D and from static to dynamic representations, showing strong promise for future applications and research.

## Conflict of Interest

The authors declare no conflict of interest.

## Author Contributions

J.Niu and W. Min contributed equally to this work. W. Min conceived and supervised the project. J. Niu and W. Min developed and implemented the SpaBatch algorithm. J. Niu, W. Min, D. Fang, J. Chen, Y. Xiong and Juan Liu validated the methods and wrote the manuscript. All authors read and approved the final manuscript.

## Supporting information

Supporting Information

## Data Availability

The data that support the findings of this study are openly available in Zenodo at https://doi.org/10.5281/zenodo.15233992 , reference number 15233992.
