# Supplementary material for: SpaBatch: Deep Learning‐Based Cross‐Slice Integration and 3D Spatial Domain Identification in Spatial Transcriptomics
Source: Adv Sci (Weinh). 2025 Sep 15;12(44):e09090. doi: 10.1002/advs.202509090 (PMC12667491; doi:10.1002/advs.202509090)
Supplement: Supplementary file 1 — Supporting Information [file ADVS-12-e09090-s001.pdf]

Supplementary Materials for
SpaBatch: Deep learning-based cross-slice integration and 3D spatial domain identification in spatial transcriptomics

Jinyun Niu[1], Donghai Fang[1], Jinyu Chen[2], Yi Xiong[3], Juan Liu[4], and Wenwen Min[1]

School of Information Science and Engineering, Yunnan University, 650091, Yunnan, China[1]
School of Mathematics, Statistics and Mechanics, Beijing University of Technology, Beijing 100124, China[2]
School of Life Sciences and Biotechnology, Shanghai Jiao Tong University, Shanghai 200240, China[3]
School of Artificial Intelligence, School of Computer Science, Wuhan University,
Wuhan 430072, Hubei, Chain[4]

1 Supplementary Figures

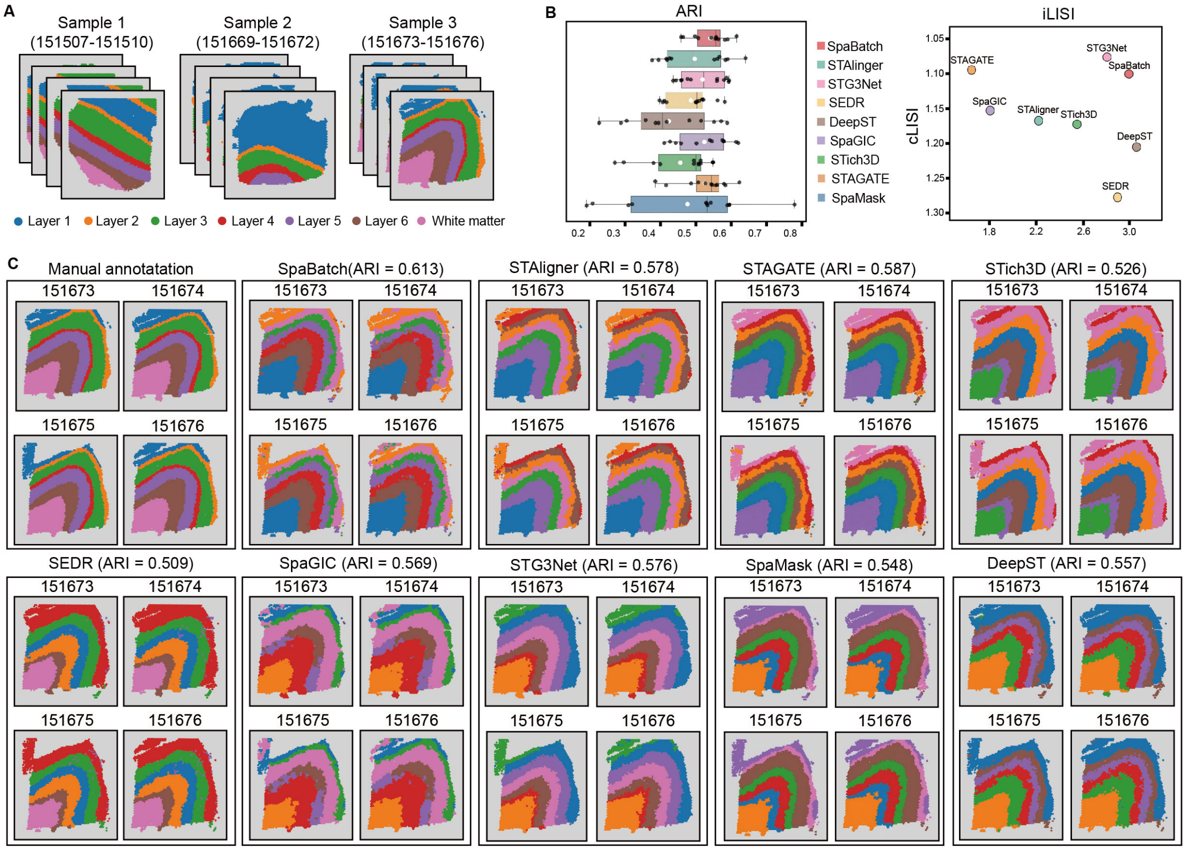

Fig. S1 Results of multi-slice joint analysis on the DLPFC dataset. (**A**) Three samples of the DLPFC dataset and their manual annotations. (**B**) Boxplots of ARI values calculated by SpaBatch and other methods across 12 slices in three samples of the DLPFC dataset. In the boxplots, the central line and the solid white dot represent the median and the mean, respectively. The swarm plot illustrates the accuracy distribution across all slices (left). The iLISI and cLISI scores calculated for SpaBatch and other methods on three samples of the DLPFC dataset are shown (right). The x-axis represents batch mixing scores, and the y-axis represents spatial domain mixing scores. Points closer to the top-right corner indicate better performance. (**C**) Results of SpaBatch and baseline methods in integrating the four slices of Sample 3 from the DLPFC dataset and identifying the spatial domains.

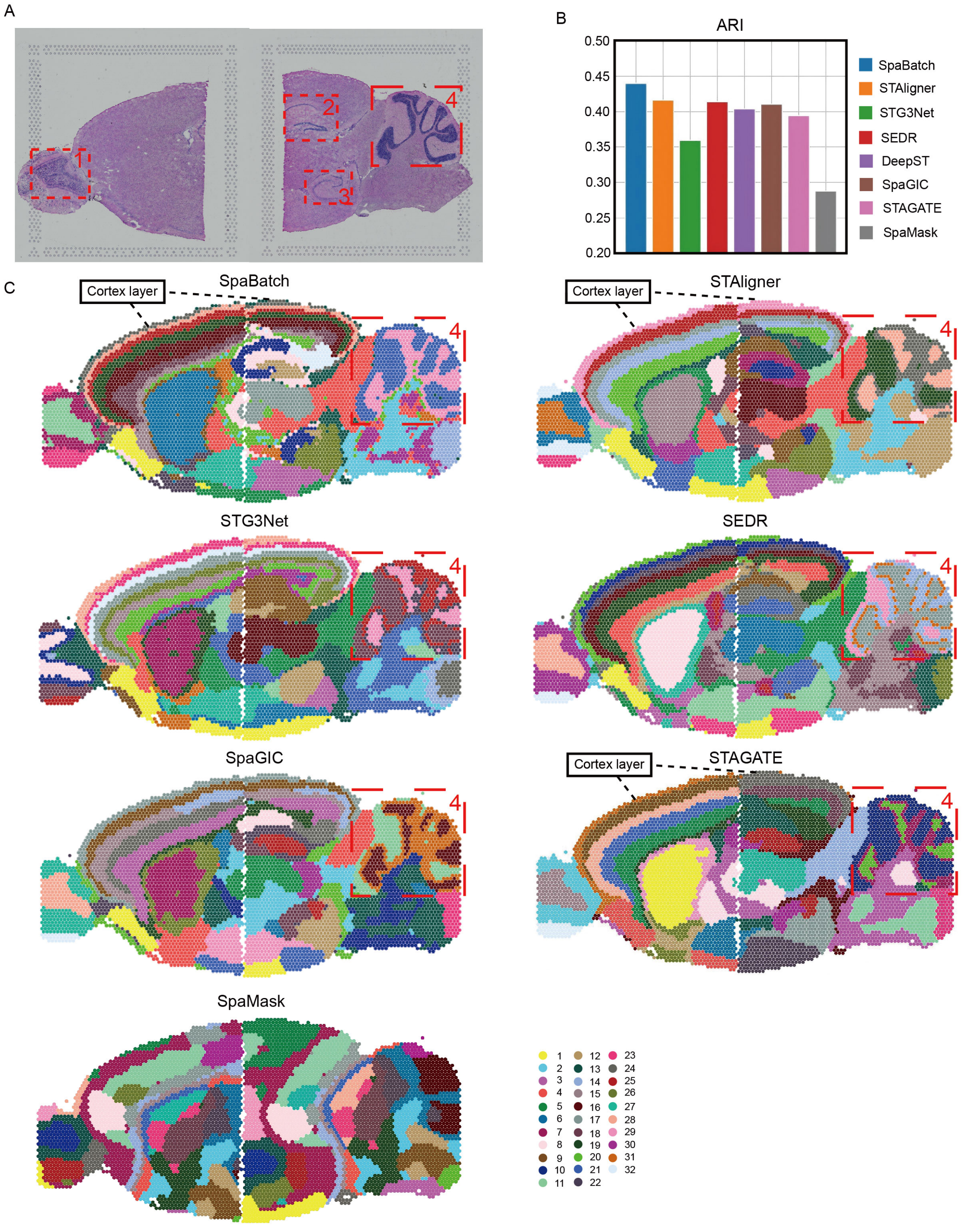

Fig. S2 Results of multi-slice joint analysis of the sagittal mouse brain dataset. (**A**) H&E images of sagittal anterior and posterior sections of Section 1, and the corresponding specific spatial subdomains. (**B**) The bar plot comparing the ARI values obtained from SpaBatch and other methods with the manual annotation. (**C**) The spatial domain identification results of sagittal mouse brain Section 1 integrated by SpaBatch and other methods.

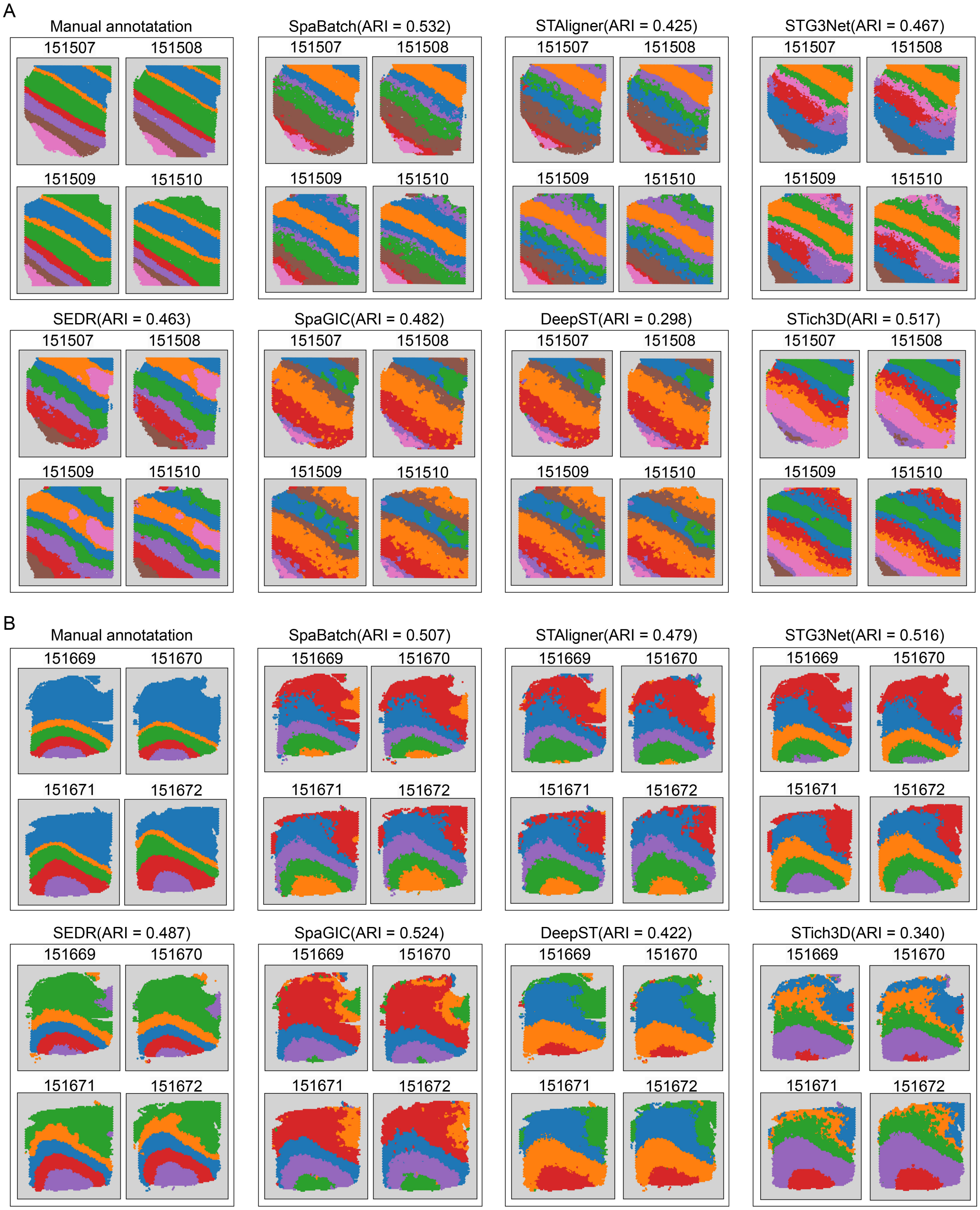

Fig. S3 Spatial domain identification results obtained by different methods on four slices from **(A)**. Donor 1 and **(B)**. Donor 2.

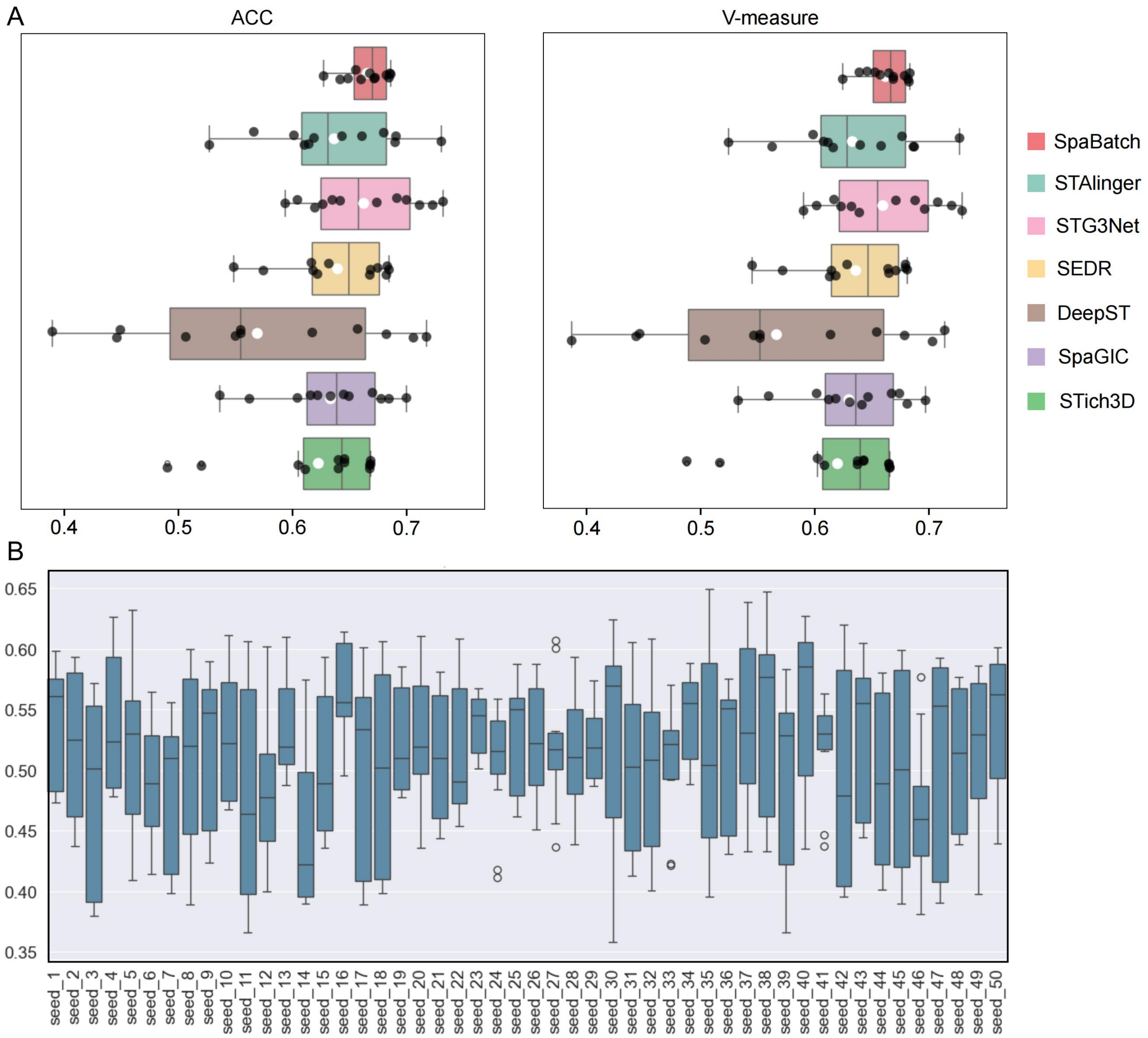

Fig. S4 Comparison of SpaBatch and baseline methods on the DLPFC dataset in terms of clustering metrics ARI and V-measure, as well as performance evaluation of SpaBatch under different random seeds. **(A)**. Boxplot of clustering accuracy across all the 12 slices grouped by Donor of the DLPFC dataset in terms of Average Clustering Consistency (ACC) and V-measure. **(B)**. The clustering ARI of SpaBatch in all 12 slices grouped by Donor under the default hyperparameters with different random seeds.

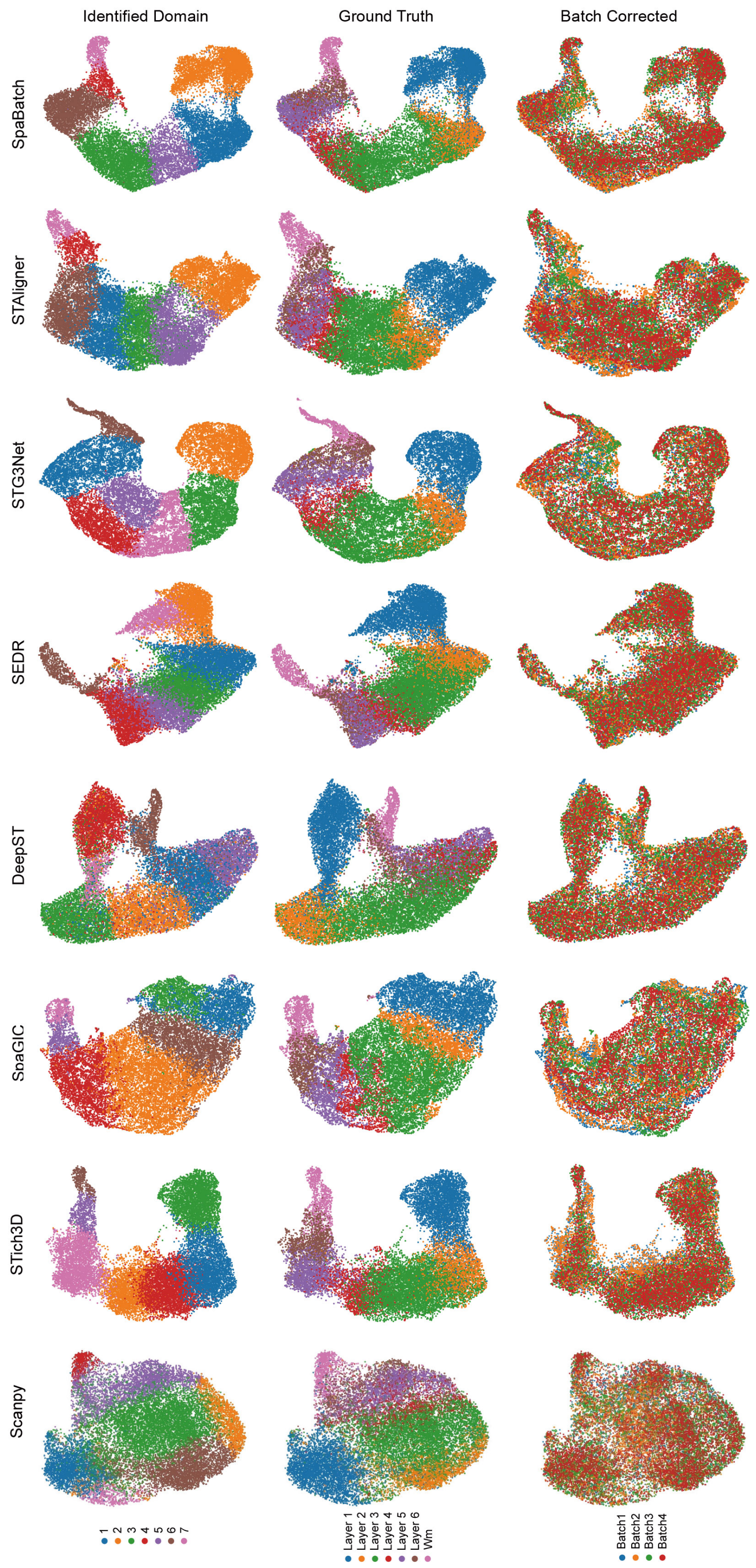

Fig. S5 UMAP visualization of the embeddings on Donor 1 from the DLPFC dataset, colored by identified spatial domains (left), ground truth (middle), and batch-corrected results (right).

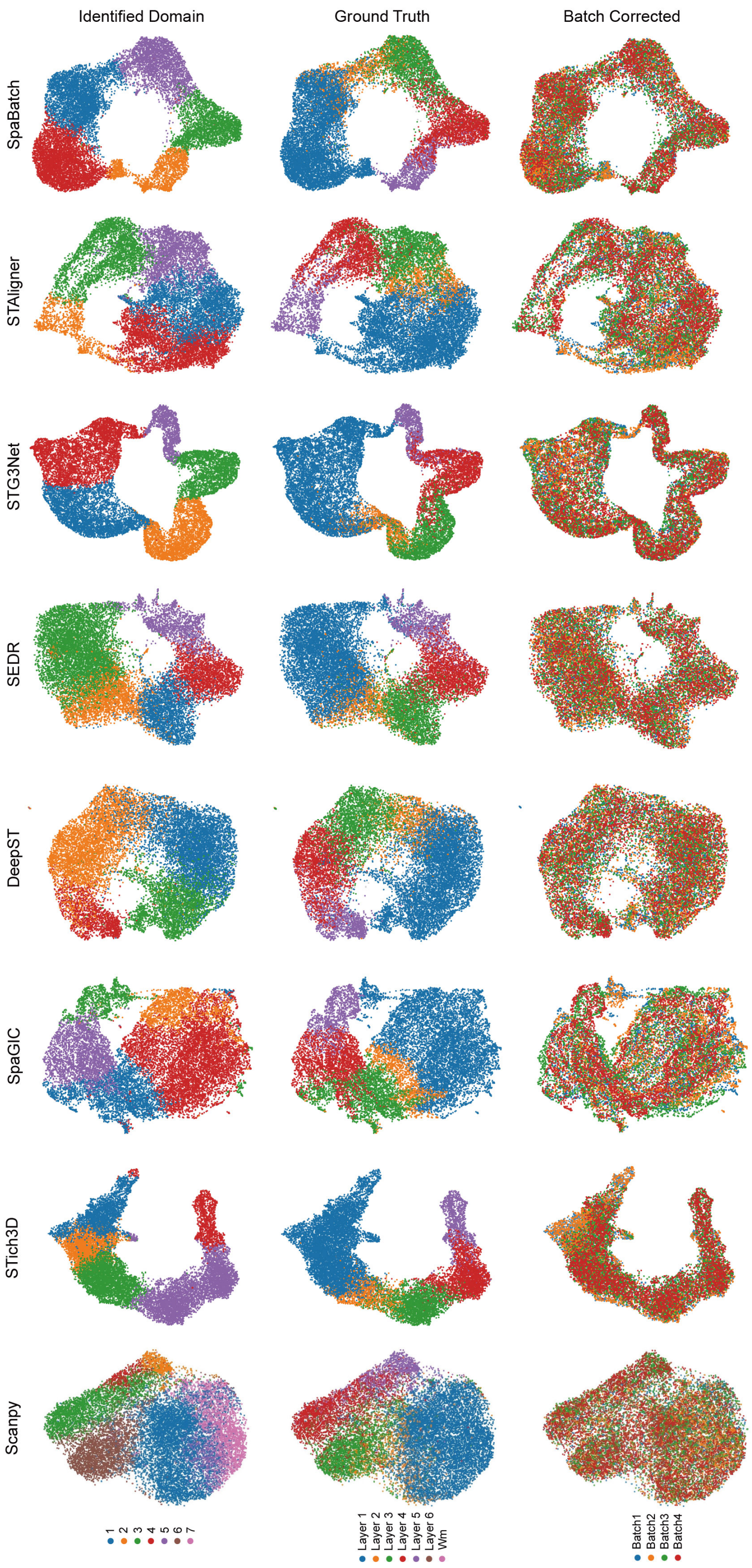

Fig. S6 UMAP visualization of the embeddings on Donor 2 from the DLPFC dataset, colored by identified spatial domains (left), ground truth (middle), and batch-corrected results (right).

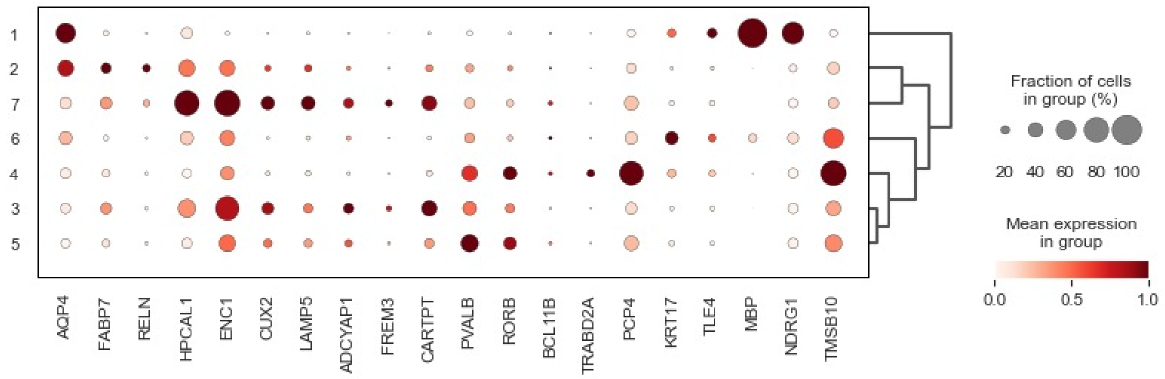

Fig. S7 Dot plot of layer marker genes defined by SpaBatch, visualizing the expression patterns of marker genes across the seven layers (layer 1−6 and WM) identified in sample 3 of the DLPFC dataset.

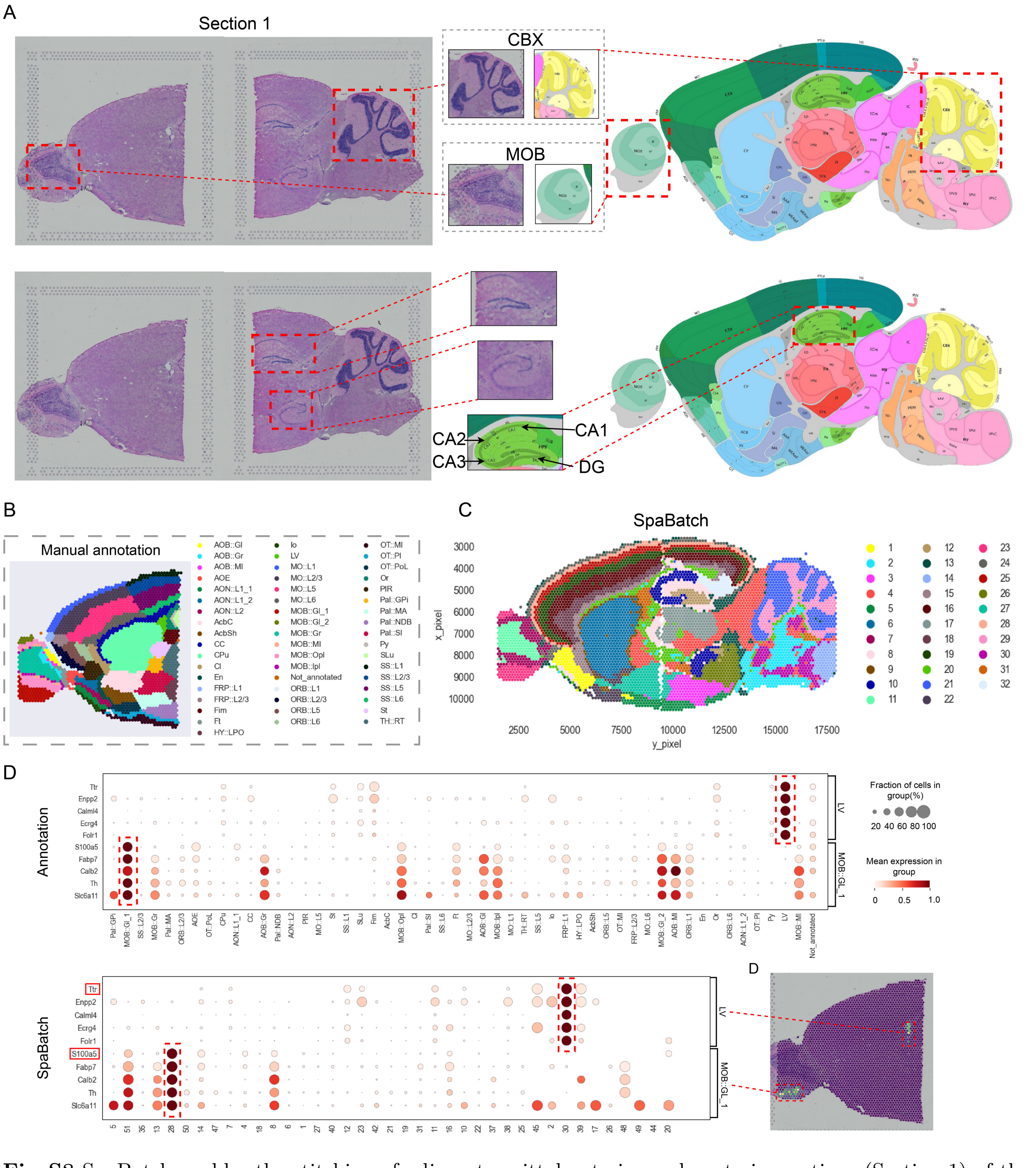

Fig. S8 SpaBatch enables the stitching of adjacent sagittal anterior and posterior sections (Section 1) of the mouse brain to create a large composite section while accurately identifying specific spatial subdomains. **(A)**. H&E images of sagittal anterior and posterior sections of Section 1, the mouse cortex reference atlas provided by the Allen Brain Reference Atlas, and the corresponding specific spatial subdomains. **(B)**. Spatial domain identification results of sagittal anterior and posterior sections of Section 1 using SpaBatch. **(C)**. Differential analysis results of different spatial domains (Domain 28 and 30) in sagittal anterior and posterior sections of Section 1 using SpaBatch, which align well with the manually annotated spatial domains (MOB::Gl and LV). **(D)**. Visualization of marker genes (*S100a5* and *Ttr*) for spatial domains 28 and 30.

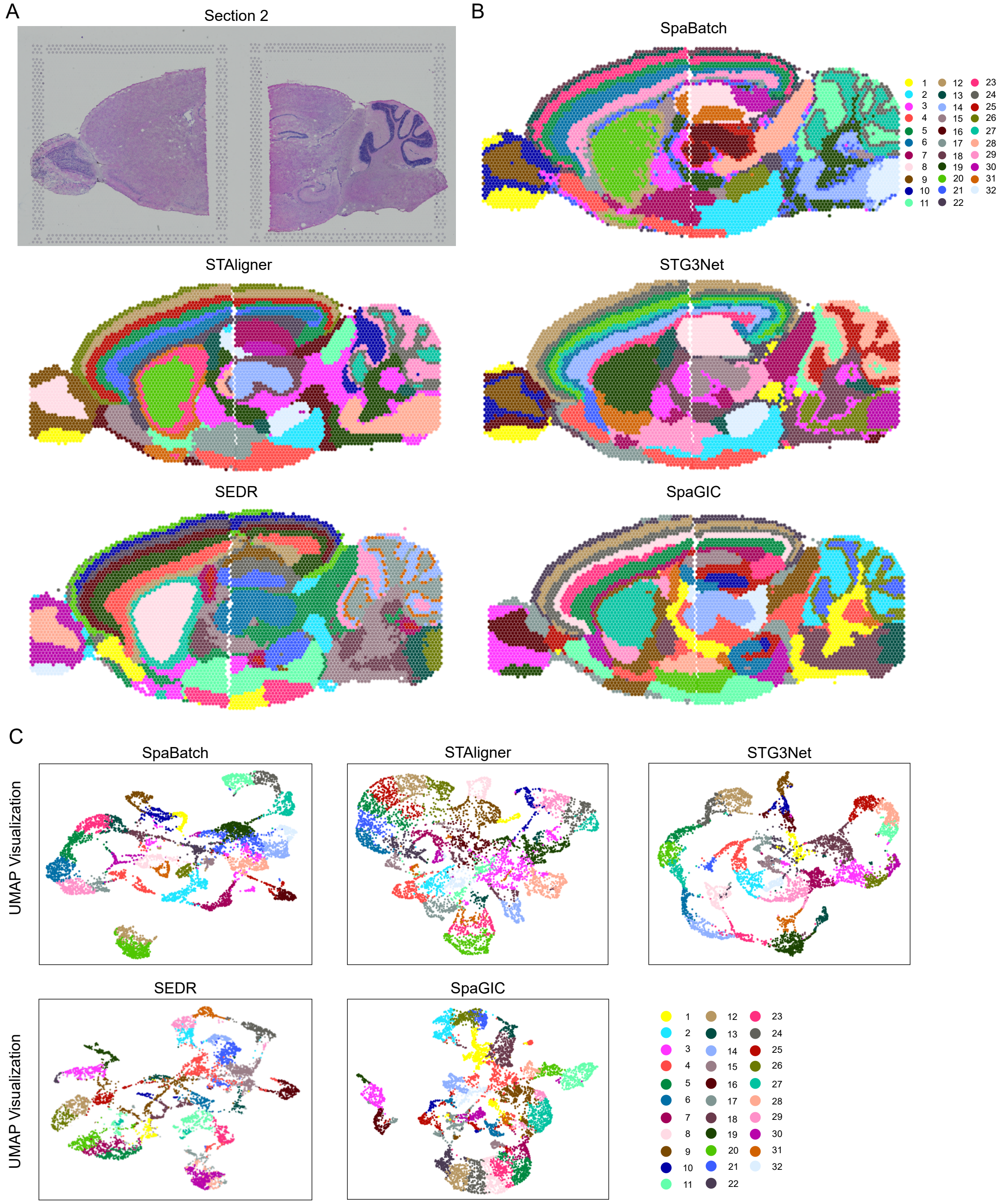

Fig. S9 SpaBatch can stitch adjacent sagittal anterior and posterior sections of the mouse brain (section 2) to generate a large composite slice. **(A)**. The H&E images of mouse sagittal anterior and posterior Section 2. **(B)**. Results of spatial domain identification using SpaBatch and other methods on the sagittal anterior and posterior Section 2 of mouse. **(C)**. Results of UMAP visualization using SpaBatch and other methods on the sagittal anterior and posterior Section 2 of mouse.

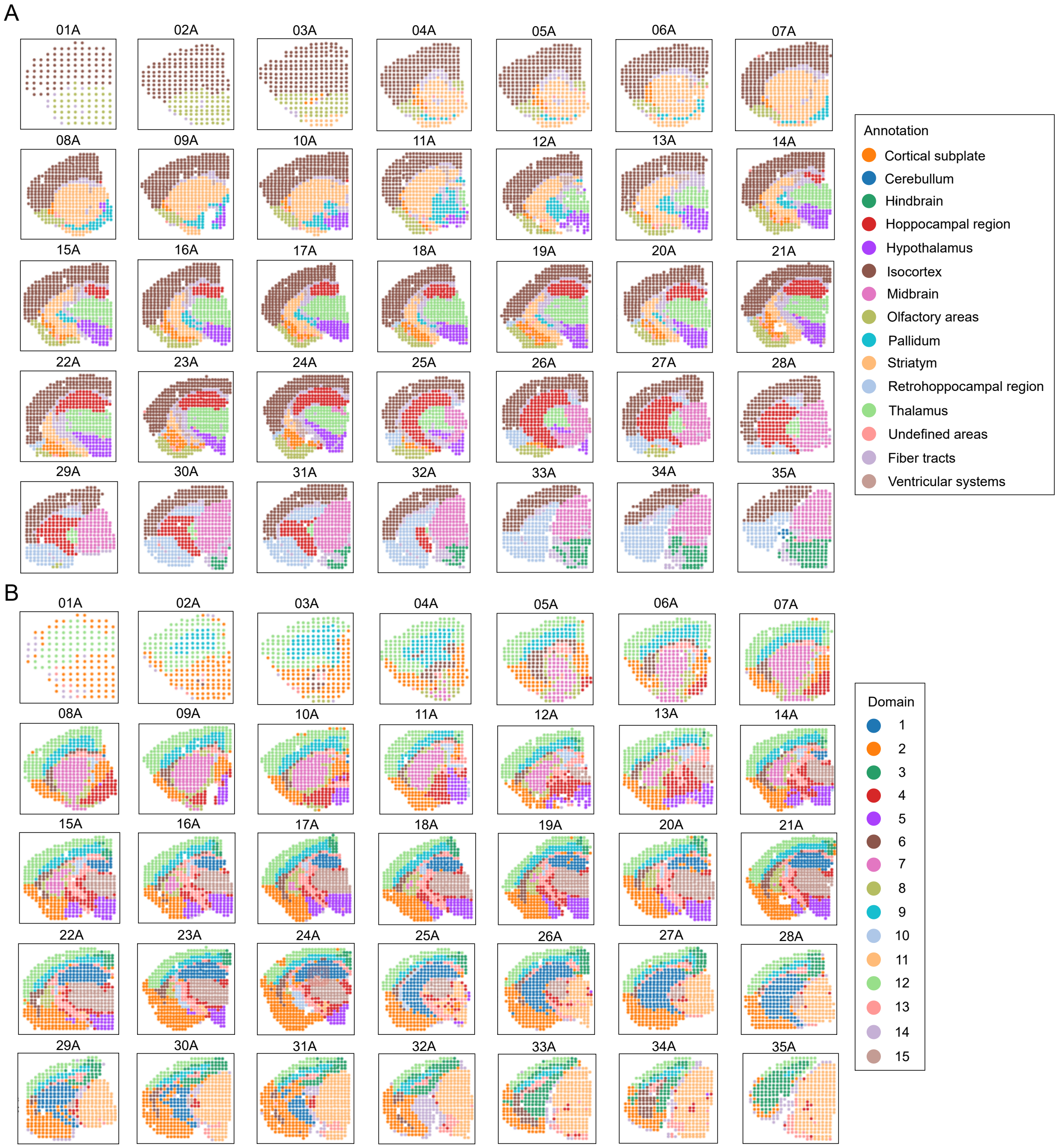

Fig. S10 (A). Manual annotation of mouse brain data from 35 slices. **(B)**. SpaBatch spatial domain identification results.

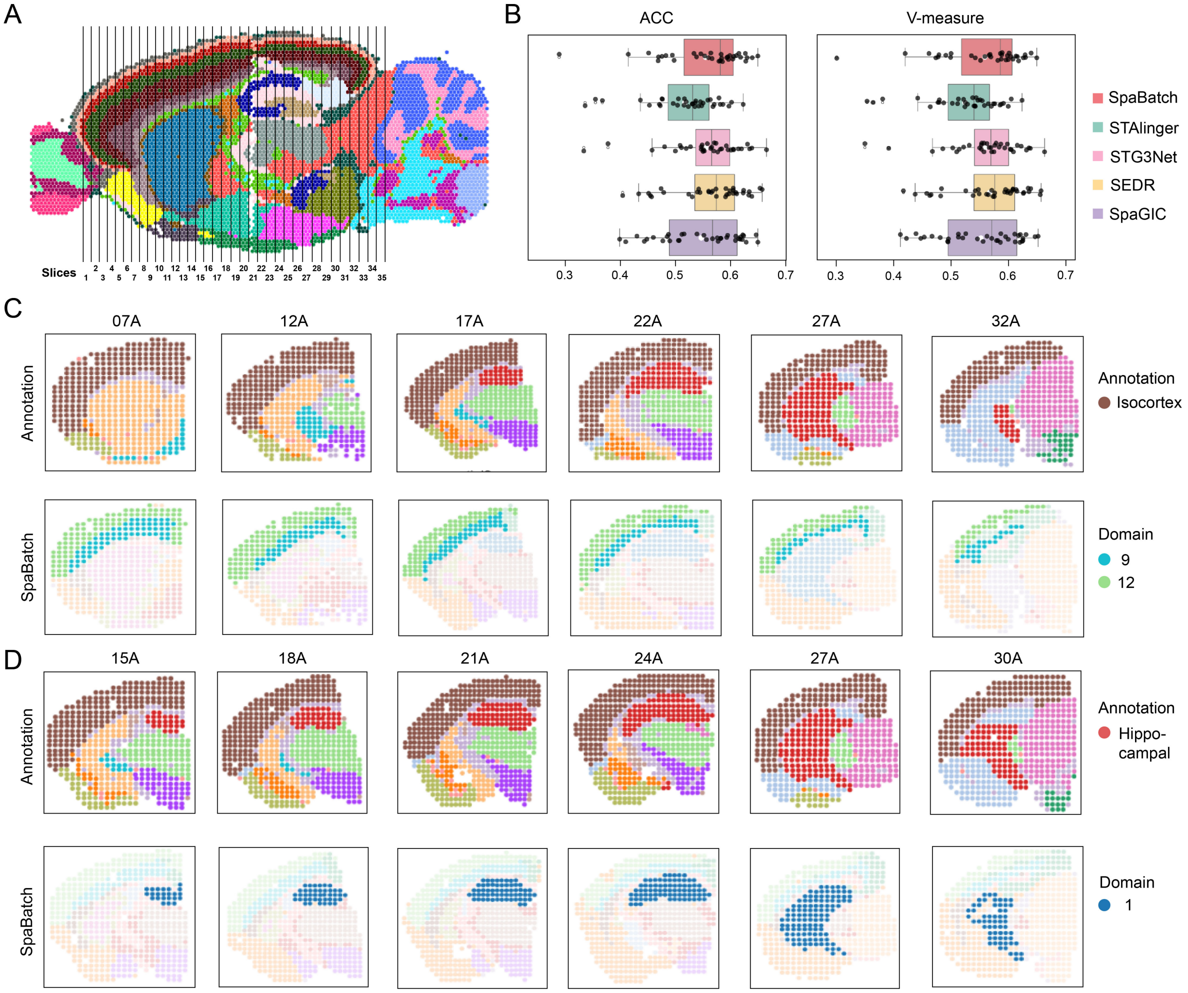

Fig. S11 **(A)**. Spatial domain identification results using SpaBatch on sagittal adult mouse brain, with the 35 slices mapped onto the sagittal mouse brain. **(B)**. Box plots of ACC and V-measure values for clustering the 35 adult mouse brain slices using SpaBatch and other methods. **(C)**. Spatial domains 9 and 12 identified by SpaBatch correspond with the manually annotated Isocortex in cross-slice spatial domain identification. **(D)**. Spatial domain 1 identified by SpaBatch corresponds with the manually annotated Hippocampal region in cross-slice spatial domain identification.

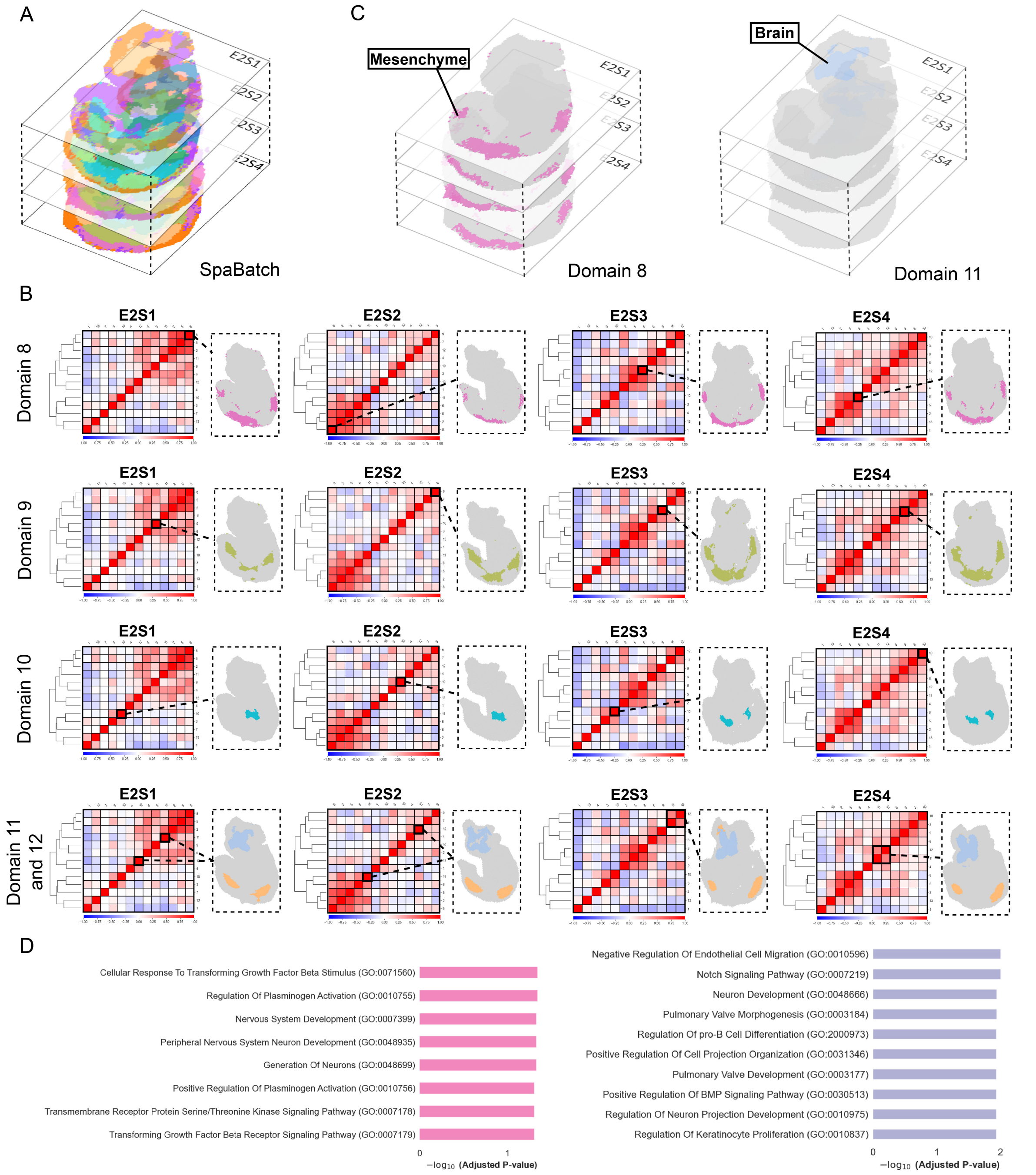

Fig. S12 (A). The spatial domains identified by SpaBatch on four sections of E2S1, E2S2, E2S3, and E2S4 in E9.5 embryos. **(B)**. The correlation matrix of clusters identified by SpaBatch highlights a group of highly correlated clusters, marked by the black box, concentrated in the mesenchyme, sclerotome, primitive gut tube, and brain regions. **(C)**. The regions exhibiting correlation in the mesenchyme and brain were consistently identified by SpaBatch across all four sections. **(D)**. GO enrichment pathway of domain 11 and 12.

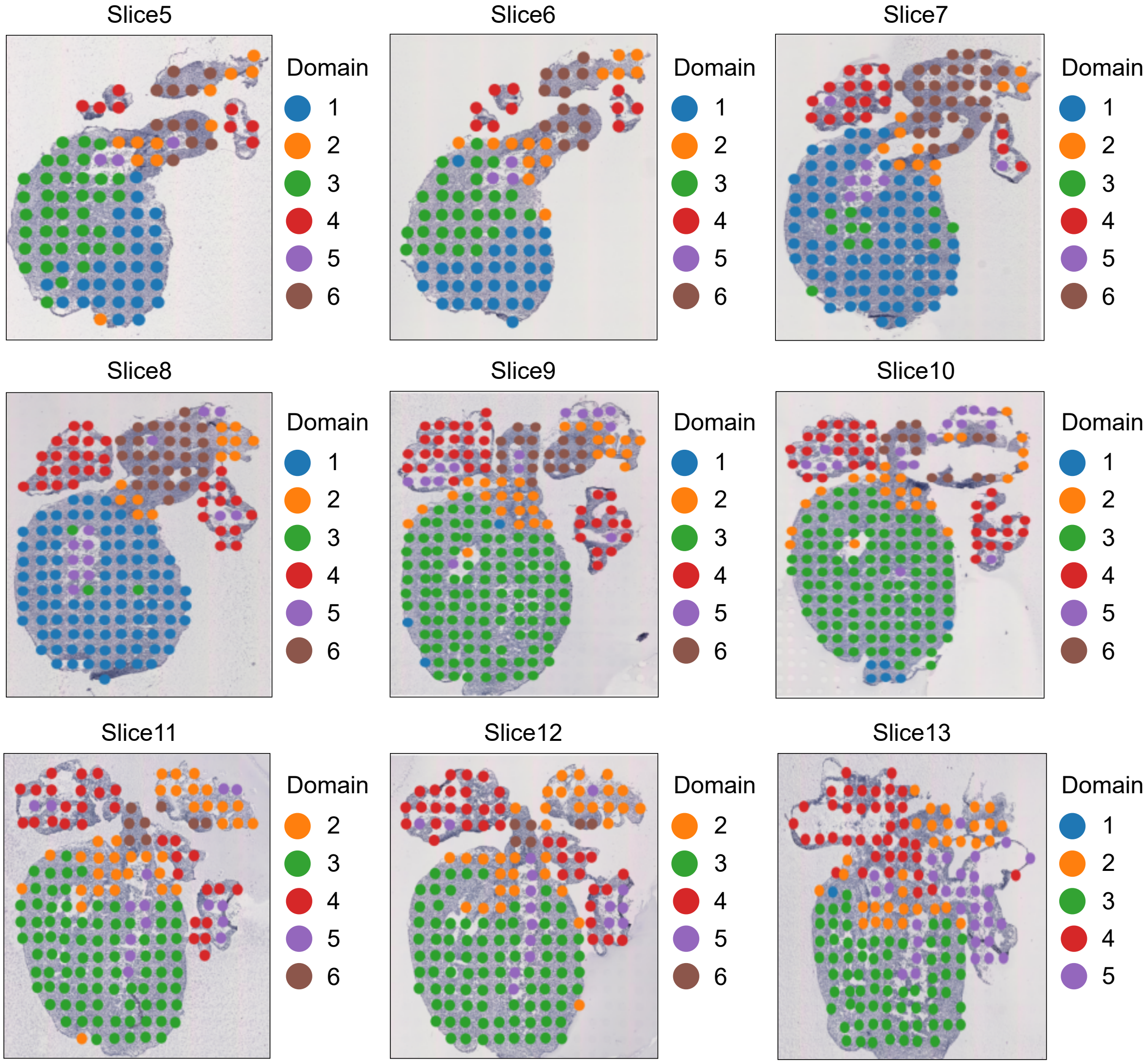

Fig. S13 SpaBatch performs spatial domain identification across all sections of the human heart at 6.5 PCW.

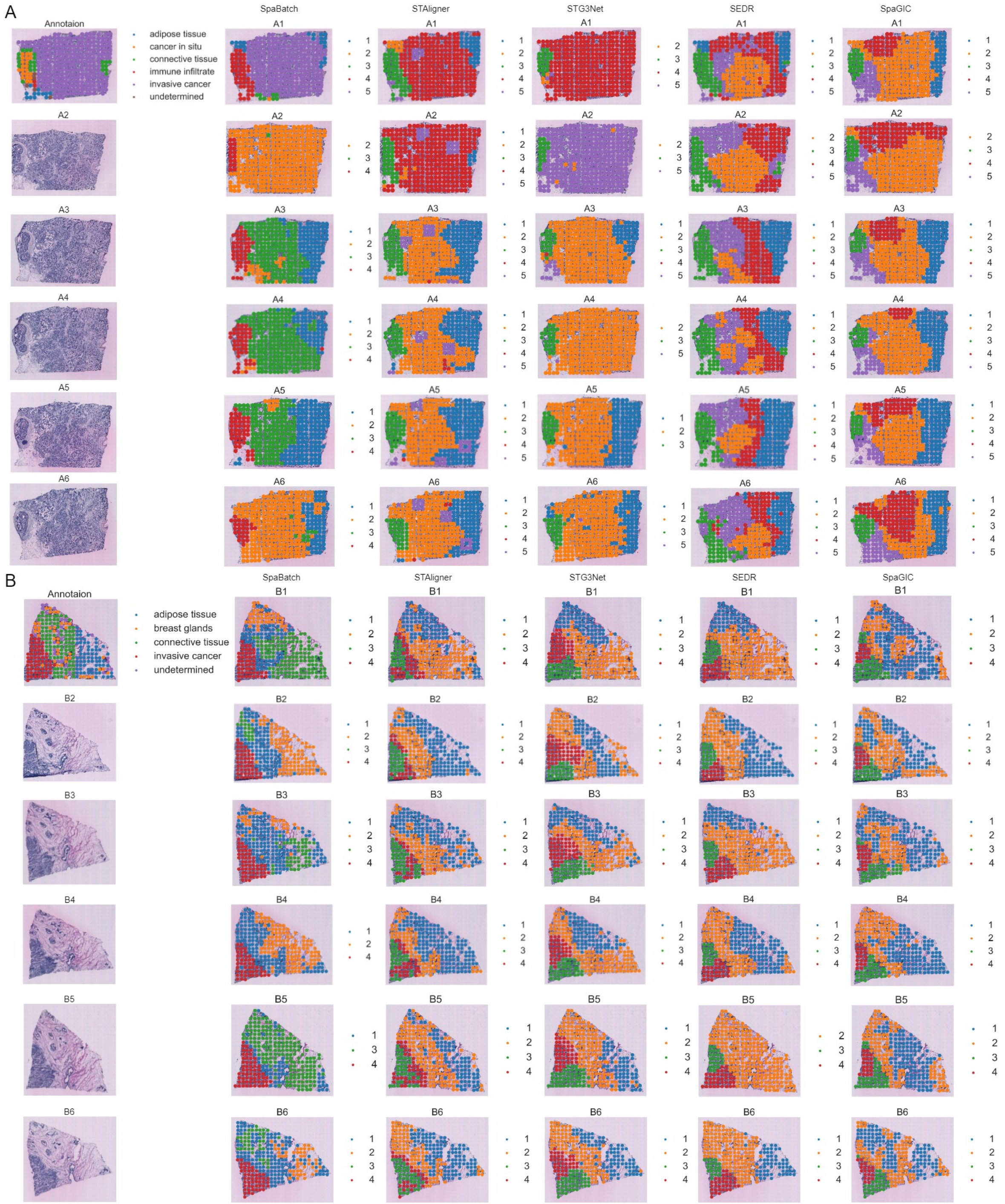

See next page

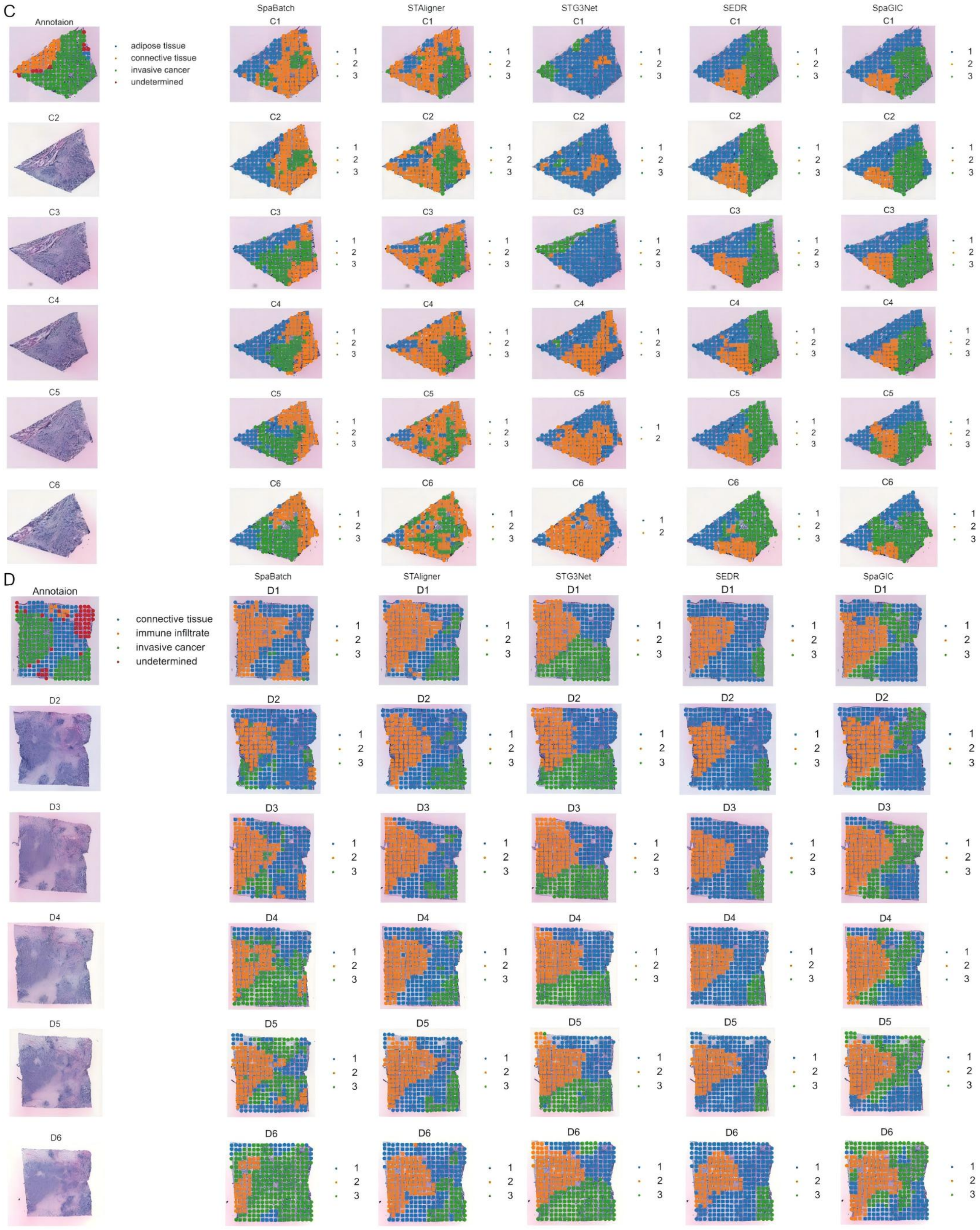

See next page

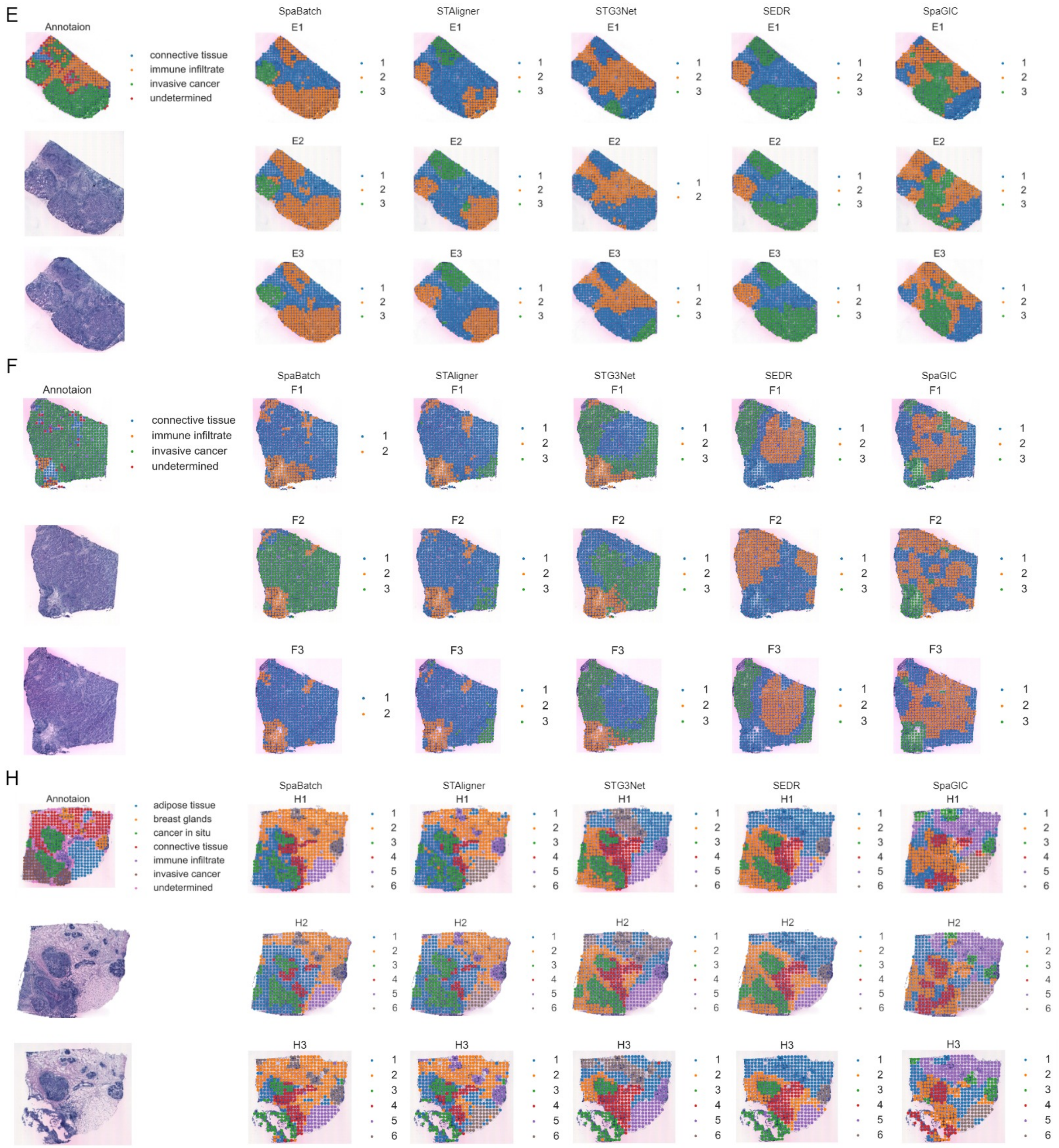

Fig. S14 Spatial domain detection results of SpaBatch and other methods across all slice groups (A-H) in the HER2-positive breast cancer dataset.

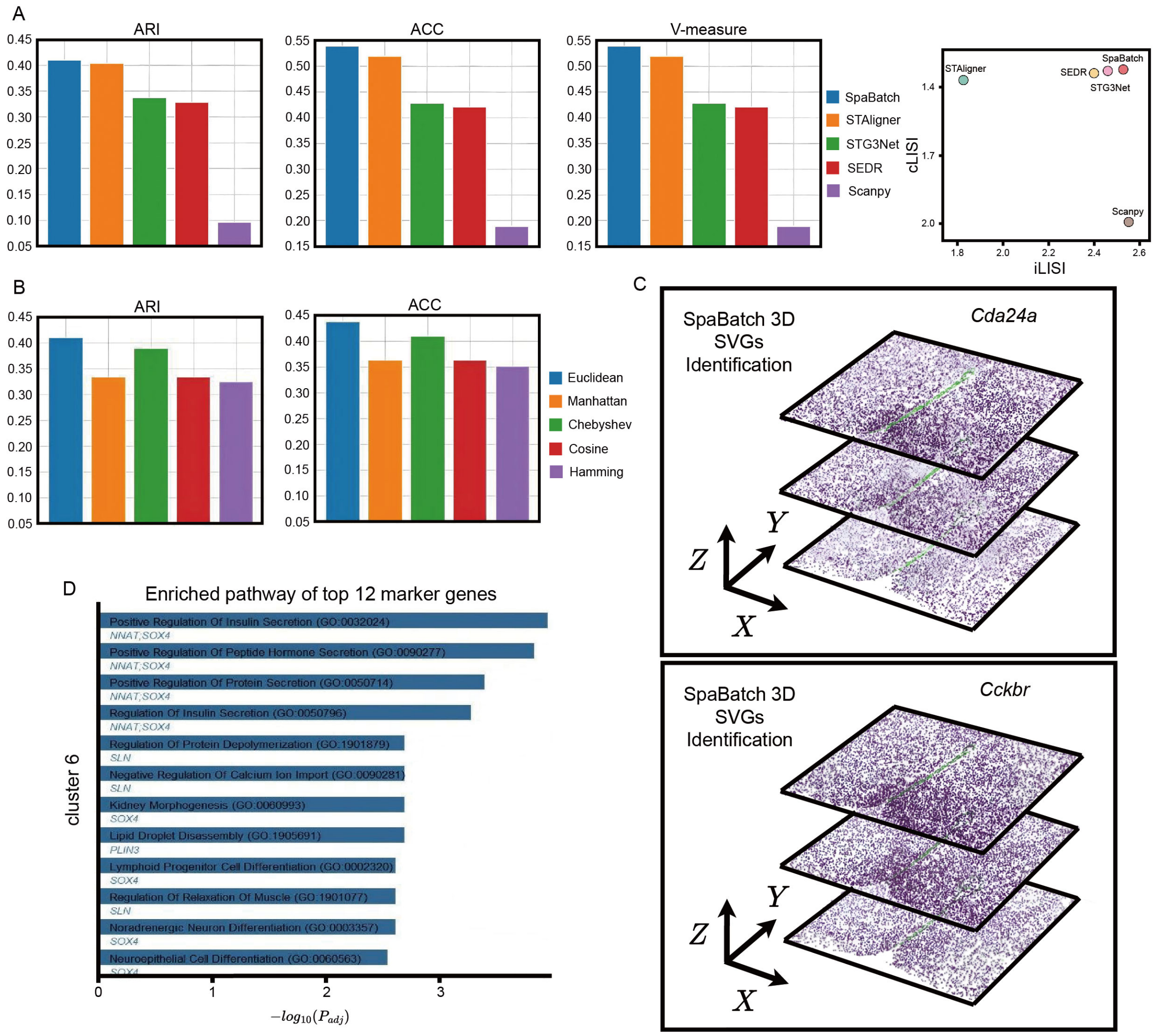

Fig. S15 SpaBatch accurately maps spatial domains in the preoptic area of the mouse hypothalamus from the MERFISH platform and enables 3D reconstruction. **(A)**. Bar plots showing ARI, ACC, V-measure and LISI scores for the three consecutive sections of the mouse hypothalamic preoptic area, obtained using SpaBatch and other methods. **(B)**. Box plots show the clustering performance of three consecutive mouse hypothalamic preoptic area slices under the SpaBatch framework, using adjacency matrices constructed with different distance metrics, including Euclidean, Manhattan, Chebyshev, Cosine, and Hamming distances. **(C)**. SpaBatch achieved the 3D spatial reconstruction of the spatially variable gene (SVGs) *Nnat* and *Cckbr* in the V3 region (Domain 6). **(D)**. The GO enrichment analysis results of the V3 region (Domain 6) show the top 12 terms.

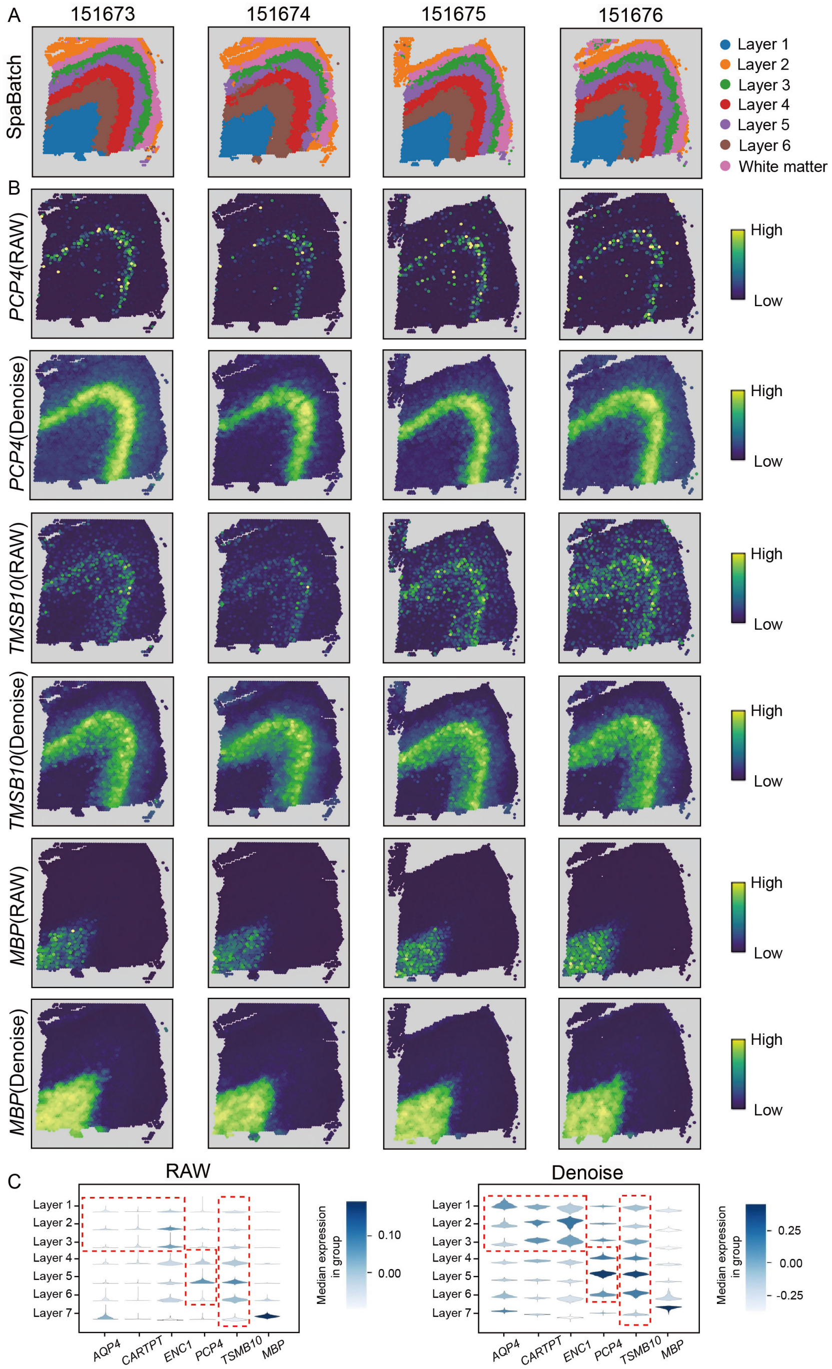

Fig. S16 SpaBatch denoises gene expression and enhances the expression patterns of spatial marker genes on the DLPFC dataset. (**A**) SpaBatch spatial domain identification results on the four slices of Donor 3 (151673-151676). (**B**) Visualization of spatial marker genes *PCP4*, *TMSB10*, and *MBP*, raw gene expression (top) versus SpaBatch-denoised gene expression (bottom). (**C**) Violin plots of raw and denoised expression of layer-specific marker genes.

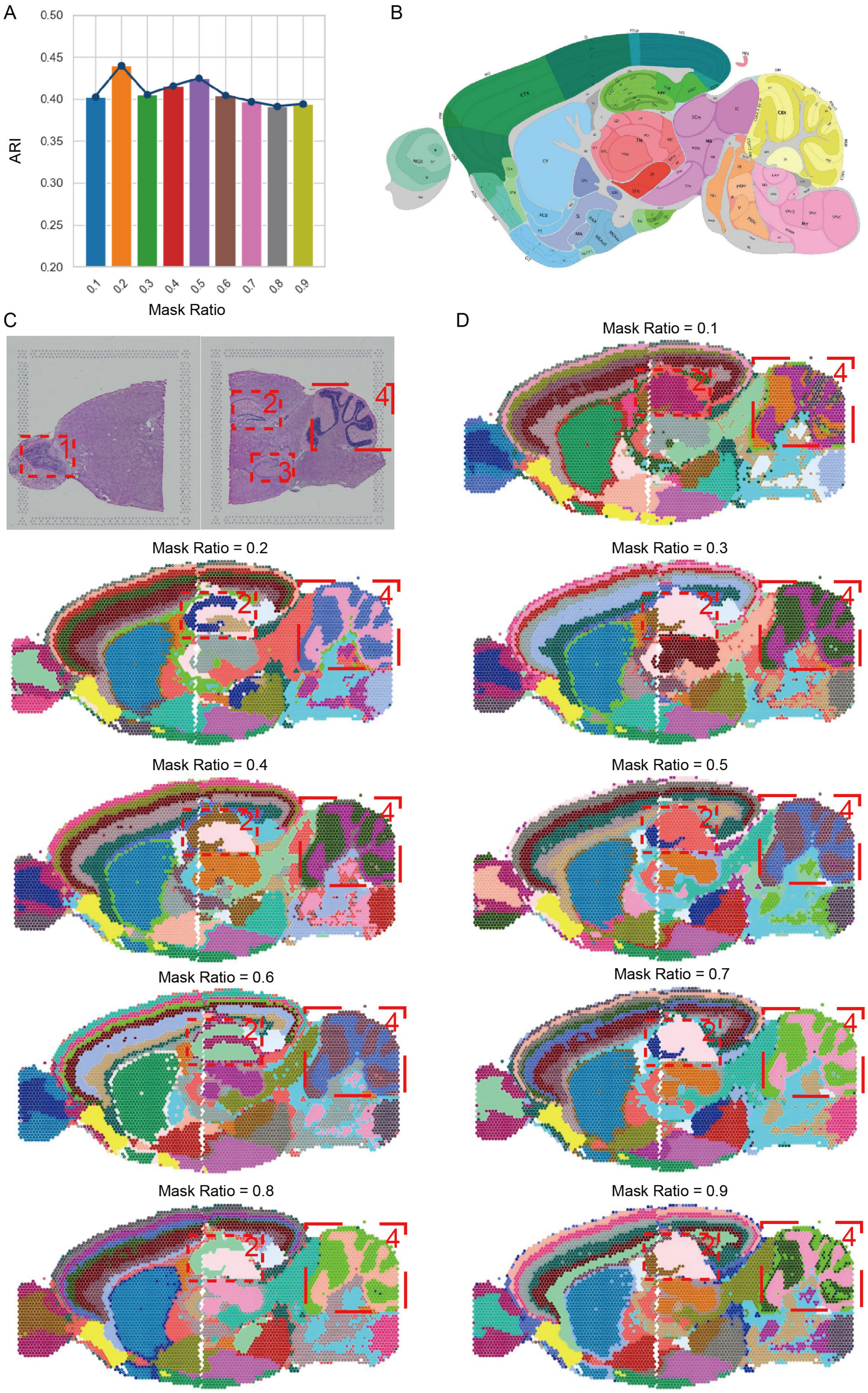

Fig. S17 Impact of mask ratio on spatial domain identification performance on the sagittal mouse brain dataset. **(A)**. The bar plot compares the changes in the clustering metric ARI under different masking rates, while the line plot represents the overall trend. **(B)** Manual annotation of the sagittal anterior mouse brain (Section 1), from the Allen Mouse Brain Reference Atlas. **(C)**. H&E images of sagittal anterior and posterior sections of Section 1, and the corresponding specific spatial subdomains. **(D)**. Spatial domain identification results on the sagittal anterior mouse brain dataset under different masking rates.

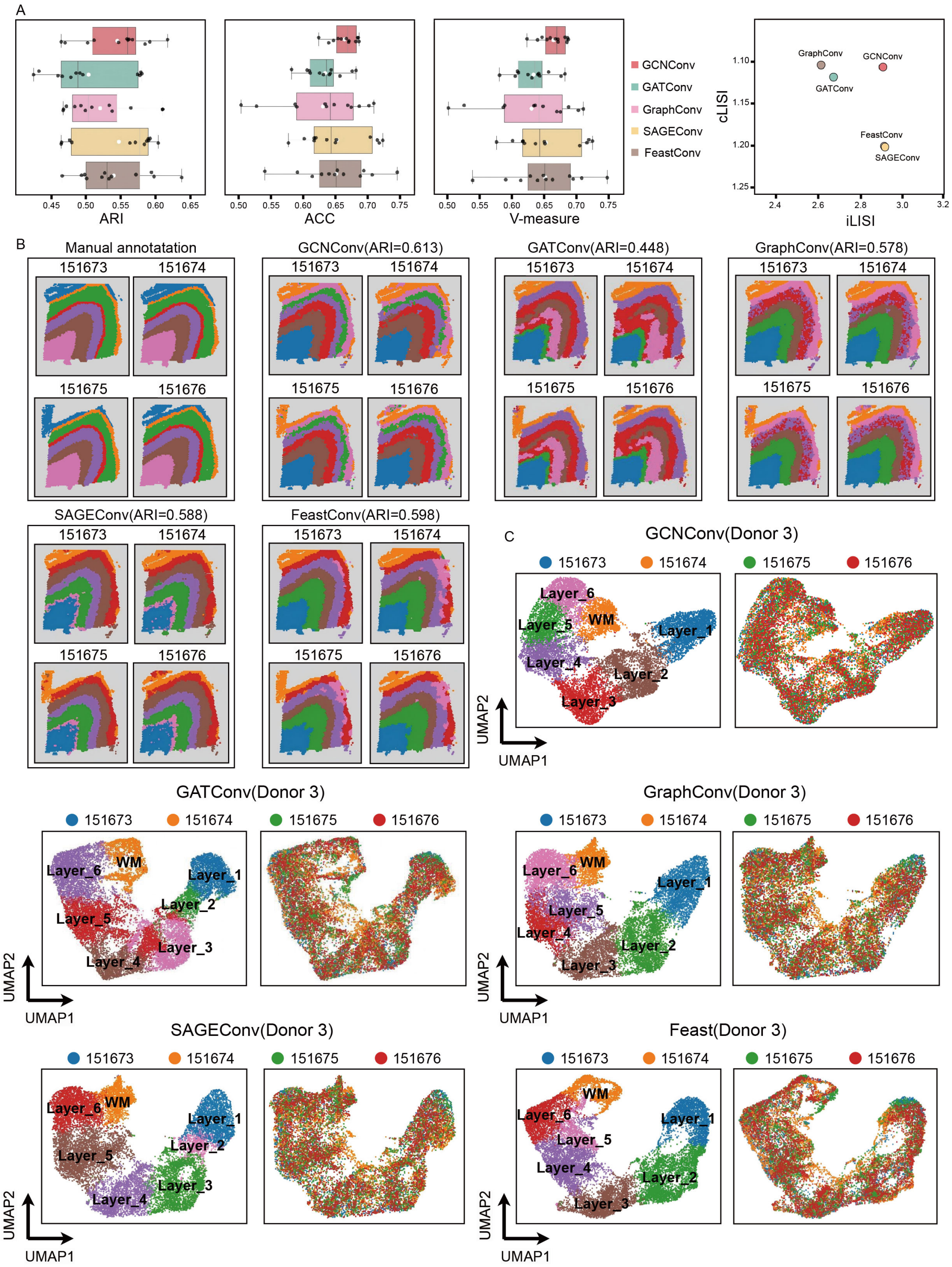

Fig. S18 The impact of applying different graph convolution layers in the backbone network on performance. (**A**) Bar plots of clustering performance in terms of ARI, ACC, and V-measure scores by applying different graph convolution layers to the SpaBatch backbone network on the DLPFC dataset. The plots also show the iLISI and cLISI scores computed on this dataset. (**B**) Integration results of four slices from sample 3 of the DLPFC dataset by different graph convolution layers, with identification of spatial domains. (**C**) UMAP visualizations of the embeddings for Donor 3 (slices 151673-151676) under different graph convolution layers ,colored by the identified domains (left) and by batch (right).

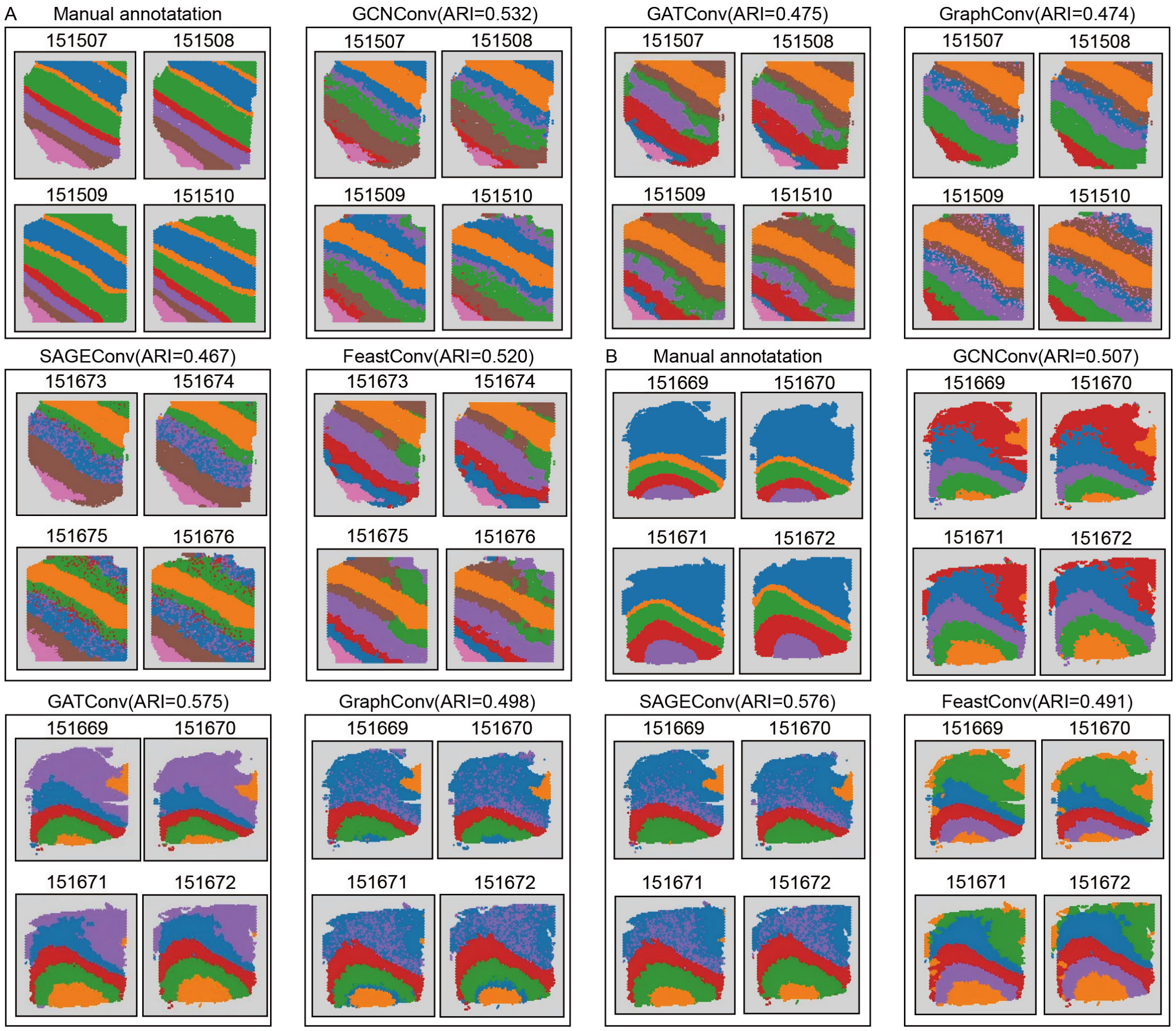

Fig. S19 Integration results of four slices from **(A)**. Donor 1 and **(B)**. Donor 2 of the DLPFC dataset by different graph convolution layers, with identification of spatial domains.

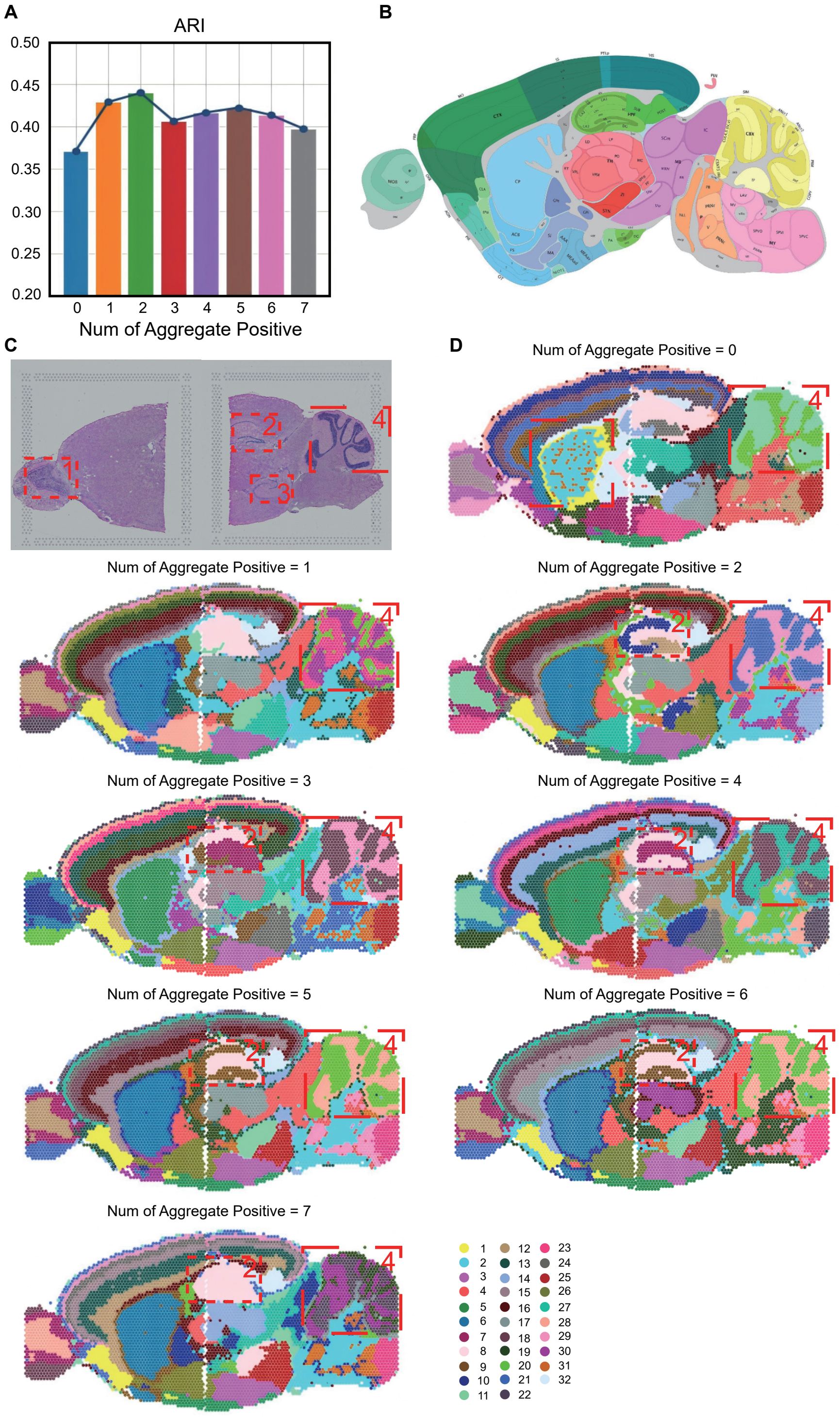

Fig. S20 The impact of the number of positive samples in the Readout triplet aggregation strategy on performance. (**A**) The bar plot compares the changes in the clustering metric ARI under different numbers of positive samples, while the line plot represents the overall trend. (**B**) Manual annotation of the sagittal anterior mouse brain (Section 1), from the Allen Mouse Brain Reference Atlas. (**C**) H&E images of sagittal anterior and posterior sections of Section 1, and the corresponding specific spatial subdomains. (**D**) Spatial domain identification results on the sagittal anterior mouse brain dataset under different number of positive samples.

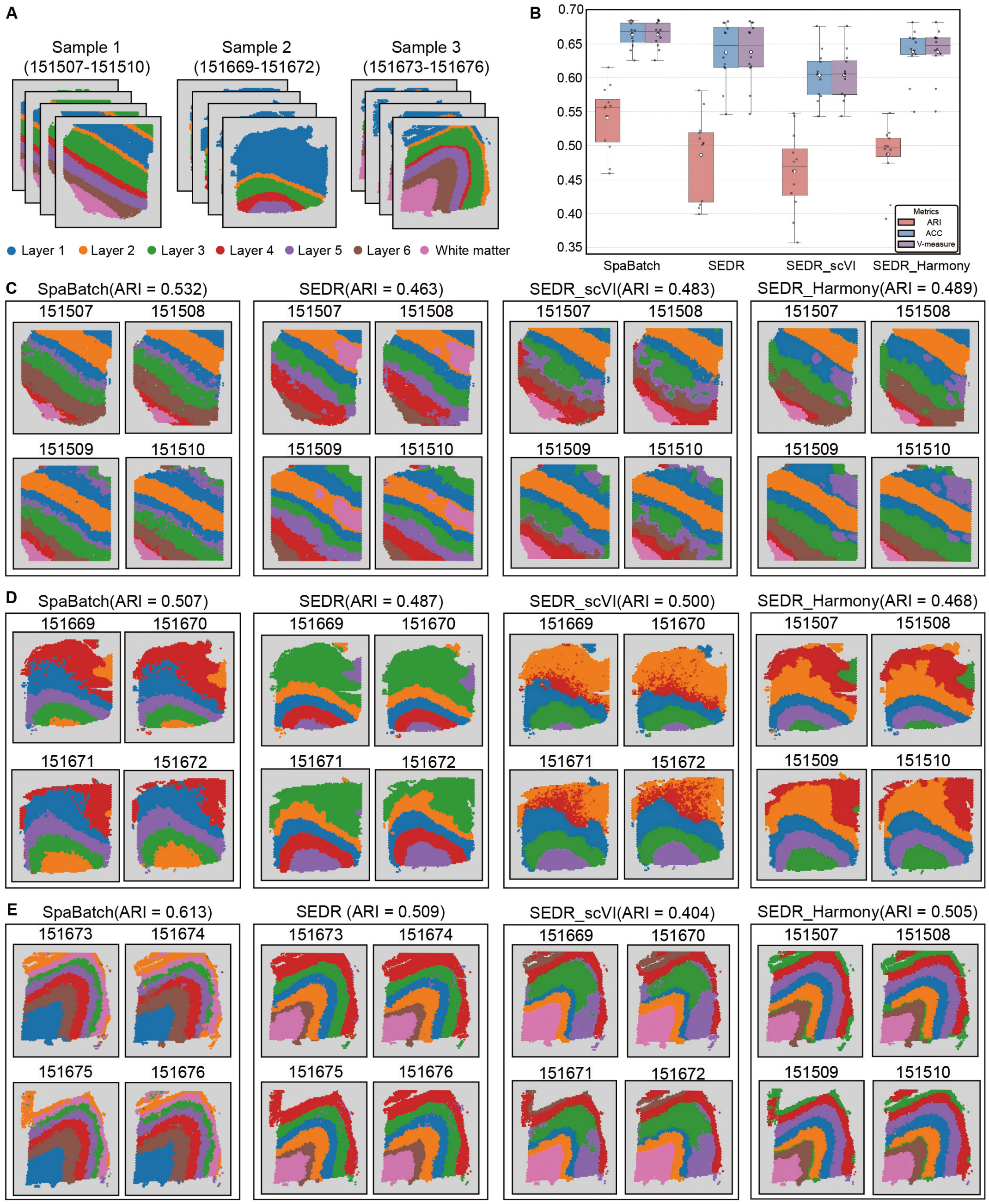

Fig. S21 Performance comparison on the DLPFC dataset with batch correction using scVI and Harmony, followed by graph neural network training. SEDR_scVI and SEDR_Harmony denote batch correction performed first using scVI and Harmony, respectively, followed by training with the graph neural network SEDR. **(A)**. Three samples of the DLPFC dataset and their manual annotations. **(B)**. Box plots compare the clustering performance of SpaBatch, SEDR, SEDR_scVI, and SEDR_Harmony on the DLPFC dataset across three metrics. **(C)**. Spatial domain identification results on the DLPFC dataset sample 1. **(D)**. Spatial domain identification results on the DLPFC dataset sample 2. **(E)**. Spatial domain identification results on the DLPFC dataset sample 3.

2 Supplementary Tables

Table S1 Summary of the ST datasets used in this study.

Platform	Tissue	Section	Number of domains	Spots	Related figures
10x Visium	Human dorsolateral prefrontal cortex (DLPFC)	151507	7	4226	Figure 2-3<br>Figure S1-S5
		151508	7	4384	
		151509	7	4789	
		151510	7	4634	
		151669	5	3661	
		151670	5	3498	
		151671	5	4110	
		151672	5	4015	
		151673	7	3639	
		151674	7	3673	
		151675	7	3592	
		151676	7	3460	
10x Visium	Sagittal mouse brain	Section 1 anterior	32	2695	Figure 4<br>Figure S6-S7
		Section 1 posterior	32	3355	
		Section 2 anterior	32	2825	
		Section 2 posterior	32	3289	
ST	Coronal mouse brain	01A	4	152	Figure 5<br>Figure S8-S9
		02A	6	240	
		03A	8	269	
		04A	8	326	
		05A	9	361	
		06A	11	403	
		07A	11	488	
		08A	12	470	
		09A	10	491	
		10A	12	522	
		11A	12	494	
		12A	13	506	
		13A	12	509	
		14A	14	580	
		15A	13	556	
		16A	14	560	
		17A	14	580	
		18A	14	577	
		19A	13	591	
		20A	13	536	
		21A	14	620	
		22A	14	589	
		23A	14	574	
		24A	13	617	
		25A	13	508	
		26A	12	548	
		27A	11	508	
		28A	11	460	
		29A	11	462	
		30A	11	527	
		31A	11	548	
		32A	9	524	
		33A	9	484	
		34A	10	478	
		35A	8	428	

See next page

Platform	Tissue	Section	Number of domains	Spots	Related figures
Stereo-seq	Mouse embryos	E2S1	14	5913	Figure 6<br>Figure S10
		E2S2	13	5292	
		E2S3	13	4356	
		E2S4	13	5059	
10x Visium	Human heart	PCW4.5-5 01	2	54	Figure 7<br>Figure S11
		PCW4.5-5 02	2	57	
		PCW4.5-5 03	2	66	
		PCW4.5-5 04	2	55	
		PCW6.5 01	6	100	
		PCW6.5 02	6	101	
		PCW6.5 03	6	155	
		PCW6.5 04	6	182	
		PCW6.5 05	6	212	
		PCW6.5 06	6	203	
		PCW6.5 07	6	173	
		PCW6.5 08	6	180	
		PCW6.5 09	6	174	
10x Visium	HER2-positive breast cancer	Group A 01	5	346	Figure 8<br>Figure S12
		Group A 02	5	325	
		Group A 03	5	359	
		Group A 04	5	343	
		Group A 05	5	332	
		Group A 06	5	360	
		Group B 01	4	295	
		Group B 02	4	270	
		Group B 03	4	298	
		Group B 04	4	283	
		Group B 05	4	289	
		Group B 06	4	277	
		Group C 01	3	176	
		Group C 02	3	187	
		Group C 03	3	180	
		Group C 04	3	184	
		Group C 05	3	181	
		Group C 06	3	178	
		Group D 01	3	306	
		Group D 02	3	303	
		Group D 03	3	301	
		Group D 04	3	302	
		Group D 05	3	306	
		Group D 06	3	315	
		Group E 01	3	587	
		Group E 02	3	572	
		Group E 03	3	570	
		Group F 01	3	691	
		Group F 02	3	695	
		Group F 03	3	712	
		Group H 01	6	613	
		Group H 02	6	603	
		Group H 03	6	510	

See next page

Platform	Tissue	Section	Number of domains	Spots	Related figures
MERFISH	Mouse hypothalamus	hypothalamus-0.09	8	5557	Figure 9<br>Figure S13
		hypothalamus-0.14	8	5926	
		hypothalamus-0.19	8	5803	

Table S2 Dataset information with training cost.

Dataset	Spots	Run Time	Memory
DLPFC_Donor_1	18033	218.16s	15.05GB
DLPFC_Donor_2	15284	171.25s	12.42GB
DLPFC_Donor_3	14364	160.20s	11.57GB
Sagittal_Mouse_Brain_Section1	6050	66.32s	6.52GB
Sagittal_Mouse_Brain_Section2	6114	67.32s	3.23GB
Coronal_Mouse_Brain	17381	283.89s	13.52GB
Mouse_Embryo	20620	384.43s	21.79GB
Human_Heart	1712	70.09s	2.68GB
Breast_Cancer_H	1726	25.25s	1.73GB
Mouse_Hypothalamus	17286	114.10s	10.65GB

Table S3 Overview of comparative spatial domain identification methods.

**Method**	**Methodology**	**Input Data**	**Downstream tasks**	**Link**
Scanpy [Wolf et al., 2018]	Analysis Toolkit	Gene expression data	Spatial domain identification<br>Visualization<br>Trajectory inference	https://scanpy.readthedocs.io/<br>en/stable/
STAGATE [Dong and Zhang, 2022]	Graph attention<br>autoencoders	Spatial location data<br>Gene expression data	Spatial domain identification<br>Visualization<br>Trajectory inference<br>Denosing	https://github.com/<br>zhanglabtools/STAGATE/
STAligner [Zhou et al., 2023]	Graph attention<br>autoencoders	Spatial location data<br>Gene expression data	Spatial domain identification<br>Visualization<br>Trajectory inference<br>GO enrichment analysis	https://github.com/<br>zhoux85/STAligner/
STG3Net [Fang et al., 2024]	Graph neural<br>autoencoder	Spatial location data<br>Gene expression data	Spatial domain identification<br>Visualization<br>Spatial domain detect<br>Denosing	https://github.com/<br>wenwenmin/STG3Net/
DeepST [Xu et al., 2022]	Variational graph<br>autoencoders	Spatial location data<br>Gene expression data<br>Histology information	Spatial domain identification<br>Visualization<br>Trajectory inference	https://github.com/<br>JiangBioLab/DeepST/
SEDR [Xu et al., 2024]	Variational graph<br>autoencoders	Spatial location data<br>Gene expression data	Spatial domain identification<br>Visualization<br>Trajectory inference<br>Denosing	https://github.com/<br>HzFu/SEDR/
SpaGIC [Liu et al., 2024]	Graph neural<br>autoencoder	Spatial location data<br>Gene expression data	Spatial domain identification<br>Visualization<br>Trajectory inference<br>Denosing	https://github.com/<br>Liuwei-CS/SpaGIC
STitch3D [Wang et al., 2023]	Graph neural<br>autoencoder	Spatial location data<br>Gene expression data<br>sc-RNA data	Spatial domain identification<br>Cell-type deconvolution<br>Visualization<br>Trajectory inference<br>Denosing	https://github.com/<br>YangLabHKUST/STitch3D

Table S4 Benchmark results on three metrics (ARI, ACC, V-measure) across three donor combinations on the DLPFC dataset.

Metric	Section	STAligner	STG3Net	STitch3D	SEDR	DeepST	SpaGIC	SpaBatch
ARI ↑	151507	0.419 ± 0.0187	0.486 ± 0.0133	0.515 ± 0.0119	0.413 ± 0.0181	0.361 ± 0.0195	0.413 ± 0.0288	0.508 ± 0.0035
	151508	0.433 ± 0.0098	0.478 ± 0.0092	0.496 ± 0.0078	0.418 ± 0.0133	0.292 ± 0.0153	0.428 ± 0.0131	0.498 ± 0.0013
	151509	0.421 ± 0.0126	0.459 ± 0.0295	0.548 ± 0.0081	0.501 ± 0.0057	0.231 ± 0.0078	0.546 ± 0.1274	0.564 ± 0.0325
	151510	0.427 ± 0.0156	0.446 ± 0.0245	0.509 ± 0.0098	0.522 ± 0.0314	0.308 ± 0.0301	0.540 ± 0.1314	0.557 ± 0.0332
	151669	0.396 ± 0.0048	0.461 ± 0.0016	0.300 ± 0.0035	0.408 ± 0.0144	0.397 ± 0.0160	0.460 ± 0.0348	0.465 ± 0.0046
	151670	0.379 ± 0.0154	0.438 ± 0.0029	0.275 ± 0.0096	0.399 ± 0.0114	0.420 ± 0.0129	0.445 ± 0.0357	0.459 ± 0.0036
	151671	0.553 ± 0.0071	0.580 ± 0.0001	0.383 ± 0.0043	0.561 ± 0.0088	0.483 ± 0.0091	0.614 ± 0.0407	0.544 ± 0.0055
	151672	0.590 ± 0.0144	0.580 ± 0.0001	0.401 ± 0.0072	0.581 ± 0.0095	0.389 ± 0.0103	0.578 ± 0.0017	0.558 ± 0.0051
	151673	0.566 ± 0.0089	0.560 ± 0.0002	0.515 ± 0.0059	0.504 ± 0.0625	0.515 ± 0.0583	0.620 ± 0.0643	0.556 ± 0.0155
	151674	0.639 ± 0.0520	0.592 ± 0.0006	0.496 ± 0.0315	0.519 ± 0.0265	0.589 ± 0.0240	0.574 ± 0.0616	0.589 ± 0.0217
	151675	0.562 ± 0.0052	0.593 ± 0.0002	0.548 ± 0.0037	0.510 ± 0.0136	0.551 ± 0.0143	0.503 ± 0.0499	0.615 ± 0.0283
	151676	0.583 ± 0.0044	0.560 ± 0.0011	0.509 ± 0.0033	0.503 ± 0.0276	0.572 ± 0.0269	0.580 ± 0.0425	0.582 ± 0.0092
	Mean	0.497 ± 0.0141	0.520 ± 0.0068	0.458 ± 0.0089	0.487 ± 0.0197	0.426 ± 0.0201	0.525 ± 0.0475	0.541 ± 0.0137
ACC ↑	151507	0.612 ± 0.0177	0.640 ± 0.0027	0.667 ± 0.0103	0.614 ± 0.0099	0.505 ± 0.0110	0.602 ± 0.0342	0.656 ± 0.0096
	151508	0.609 ± 0.0141	0.624 ± 0.0005	0.638 ± 0.0093	0.616 ± 0.0142	0.444 ± 0.0135	0.620 ± 0.0253	0.646 ± 0.0083
	151509	0.617 ± 0.0094	0.618 ± 0.0252	0.666 ± 0.0061	0.629 ± 0.0060	0.387 ± 0.0062	0.632 ± 0.0460	0.672 ± 0.0142
	151510	0.599 ± 0.0096	0.602 ± 0.0192	0.644 ± 0.0058	0.620 ± 0.0145	0.447 ± 0.0165	0.614 ± 0.0565	0.649 ± 0.0083
	151669	0.564 ± 0.0095	0.633 ± 0.0065	0.518 ± 0.0067	0.573 ± 0.0123	0.553 ± 0.0139	0.560 ± 0.0146	0.590 ± 0.0041
	151670	0.525 ± 0.0069	0.591 ± 0.0098	0.488 ± 0.0042	0.546 ± 0.0148	0.548 ± 0.0151	0.534 ± 0.0152	0.558 ± 0.0086
	151671	0.641 ± 0.0016	0.689 ± 0.0007	0.609 ± 0.0010	0.681 ± 0.0074	0.615 ± 0.0083	0.668 ± 0.0088	0.656 ± 0.0041
	151672	0.659 ± 0.0072	0.672 ± 0.0001	0.603 ± 0.0043	0.683 ± 0.0007	0.553 ± 0.0012	0.642 ± 0.0084	0.672 ± 0.0090
	151673	0.688 ± 0.0133	0.709 ± 0.0005	0.667 ± 0.0086	0.666 ± 0.0212	0.680 ± 0.0238	0.698 ± 0.0368	0.687 ± 0.0164
	151674	0.728 ± 0.0141	0.730 ± 0.0009	0.638 ± 0.0097	0.680 ± 0.0190	0.715 ± 0.0182	0.682 ± 0.0381	0.686 ± 0.0117
	151675	0.678 ± 0.0099	0.721 ± 0.0005	0.666 ± 0.0069	0.672 ± 0.0015	0.655 ± 0.0021	0.648 ± 0.0343	0.723 ± 0.0206
	151676	0.688 ± 0.0064	0.698 ± 0.0019	0.644 ± 0.0041	0.666 ± 0.0064	0.704 ± 0.0074	0.675 ± 0.0265	0.688 ± 0.0080
	Mean	0.634 ± 0.0100	0.661 ± 0.0068	0.621 ± 0.0069	0.637 ± 0.0116	0.567 ± 0.0119	0.631 ± 0.0275	0.657 ± 0.0106
V-measure ↑	151507	0.613 ± 0.0183	0.640 ± 0.0133	0.667 ± 0.0105	0.614 ± 0.0099	0.505 ± 0.0111	0.603 ± 0.0341	0.656 ± 0.0096
	151508	0.609 ± 0.0130	0.625 ± 0.0092	0.639 ± 0.0091	0.616 ± 0.0143	0.444 ± 0.0135	0.620 ± 0.0253	0.646 ± 0.0083
	151509	0.617 ± 0.0108	0.618 ± 0.0295	0.666 ± 0.0061	0.630 ± 0.0061	0.388 ± 0.0062	0.632 ± 0.0461	0.673 ± 0.0142
	151510	0.600 ± 0.0098	0.603 ± 0.0245	0.644 ± 0.0058	0.620 ± 0.0147	0.447 ± 0.0165	0.614 ± 0.0565	0.649 ± 0.0083
	151669	0.564 ± 0.0094	0.633 ± 0.0016	0.518 ± 0.0067	0.573 ± 0.0122	0.553 ± 0.0139	0.561 ± 0.0146	0.591 ± 0.0041
	151670	0.525 ± 0.0067	0.591 ± 0.0029	0.489 ± 0.0040	0.547 ± 0.0148	0.548 ± 0.0153	0.534 ± 0.0152	0.558 ± 0.0086
	151671	0.641 ± 0.0016	0.689 ± 0.0001	0.610 ± 0.0010	0.681 ± 0.0074	0.615 ± 0.0082	0.668 ± 0.0088	0.656 ± 0.0041
	151672	0.659 ± 0.0072	0.672 ± 0.0001	0.603 ± 0.0041	0.683 ± 0.0006	0.553 ± 0.0012	0.643 ± 0.0084	0.672 ± 0.0090
	151673	0.688 ± 0.0132	0.710 ± 0.0002	0.667 ± 0.0086	0.667 ± 0.0212	0.681 ± 0.0240	0.698 ± 0.0368	0.687 ± 0.0164
	151674	0.729 ± 0.0141	0.731 ± 0.0006	0.639 ± 0.0100	0.681 ± 0.0190	0.716 ± 0.0183	0.683 ± 0.0381	0.686 ± 0.0117
	151675	0.678 ± 0.0097	0.721 ± 0.0002	0.666 ± 0.0069	0.673 ± 0.0015	0.656 ± 0.0021	0.648 ± 0.0343	0.723 ± 0.0206
	151676	0.689 ± 0.0062	0.698 ± 0.0011	0.644 ± 0.0043	0.666 ± 0.0064	0.705 ± 0.0072	0.676 ± 0.0265	0.688 ± 0.0080
	Mean	0.634 ± 0.0100	0.661 ± 0.0070	0.621 ± 0.0064	0.638 ± 0.0116	0.568 ± 0.0117	0.632 ± 0.0275	0.657 ± 0.0106

3 Supplementary Algorithm

Algorithm 1 SpaBatch Algorithm for Spatial Domain Identification

Input: Multi-slice gene expression matrix X, multi-slice spatial location matrix, masking rate ρ, number of pre-training epochs T_p, number of fine-tuning epochs T_f, linear encoder L_e, graph encoder G_e, graph decoder G_d.

Output: Spatial domain identification results.

Obtain the preprocessed feature matrix X;
Construct the cross-slice spatial adjacency matrix A;
Pre-training phase:
for $t = 0$ to $T_p - 1$ **do**
    Divide the spot set into a masked subset V_m and an unmasked subset using the ρ;
    Compute the masked feature matrix X_{mask};
    Learn the low-dimensional representation $Z_f = L_e(X_{mask})$;
    Obtain the output of the first graph convolutional layer $Z'_g = G_e(A, Z_f)$;
    Obtain μ and $\log(\sigma^2)$ from the second graph convolutional layer according to Eq. (3);
    Integrate μ and $\log(\sigma^2)$ using the reparameterization method to obtain Z_g;
    Concatenate Z_f and Z_g to obtain the low-dimensional embedding Z;
    Reconstruct the spatial adjacency matrix $\widetilde{A}$ via the inner product decoder;
    Reconstruct the feature $\widetilde{X}_{mask} = G_d(A, Z)$;
    Calculate the reconstruction adjacency matrix loss $\mathcal{L}_{\text{graph}}(A, \widetilde{A})$ according to Eq. (7);
    Calculate the Kullback−Leibler (KL) loss $\mathcal{L}_{\text{KL}}(\mu, \log(\sigma^2))$ according to Eq. (8);
    Calculate the reconstruction feature loss $\mathcal{L}_{\text{sce}}(X_{mask}, \widetilde{X}_{mask})$ according to Eq. (9);
    Add $\mathcal{L}_{\text{graph}}$, $\mathcal{L}_{\text{KL}}$, and $\mathcal{L}_{\text{sce}}$ to obtain $\mathcal{L}_{\text{VGAE}}$;
    Update the parameters of L_e, G_e, and G_d by minimizing $\mathcal{L}_{\text{VGAE}}$;
end
Fine-tuning phase:
for $t = 0$ to $T_f - 1$ **do**
    Initialize the clustering layer in Deep Embedded Clustering (DEC) using the embeddings Z obtained from the pre-training phase;
    Initialize the triplet positive and negative pairs based on the readout strategy using the embeddings Z obtained from the pre-training phase;
    Compute Z and $\mathcal{L}_{\text{VGAE}}$ using the same strategy as in the pre-training phase;
    Compute $\mathcal{L}_{\text{DEC}}$ every 20 epochs based on Z according to Eq. (12);
    Compute $\mathcal{L}_{\text{Tri}}$ every 500 epochs based on Z according to Eq. (13);
    Construct the overall loss function $\mathcal{L}_{\text{Overall}}$ using $\mathcal{L}_{\text{VGAE}}$, $\mathcal{L}_{\text{DEC}}$, and $\mathcal{L}_{\text{Tri}}$ according to Eq. (14);
    Update the parameters of L_e, G_e, and G_d by minimizing $\mathcal{L}_{\text{Overall}}$;
end
Compute the final embedding Z according to encoder;
Cluster the latent representation Z using mclust method;
return Clustering results, where each cluster corresponds to a **3D spatial domain**.

References

Dong and Zhang, 2022. Dong, K. and Zhang, S. (2022). STAGATE: Deciphering spatial domains from spatially resolved transcriptomics with an adaptive graph attention auto-encoder. *Nature Communications*, 13(1):1739.

Fang et al., 2024. Fang, D., Zhu, F., et al. (2024). Multi-slice spatial transcriptomics data integration analysis with stg3net. In *2024 IEEE International Conference on Bioinformatics and Biomedicine (BIBM)*, pages 509–514. IEEE.

Liu et al., 2024. Liu, W., Wang, B., Bai, Y., Liang, X., Xue, L., and Luo, J. (2024). Spagic: graph-informed clustering in spatial transcriptomics via self-supervised contrastive learning. *Briefings in Bioinformatics*, 25(6):bbae578.

Wang et al., 2023. Wang, G., Zhao, J., Yan, Y., et al. (2023). Construction of a 3D whole organism spatial atlas by joint modelling of multiple slices with deep neural networks. *Nature Machine Intelligence*, 5(11):1200–1213.

Wolf et al., 2018. Wolf, F. A., Angerer, P., et al. (2018). SCANPY: large-scale single-cell gene expression data analysis. *Genome Biology*, 19:1–5.

Xu et al., 2022. Xu, C., Jin, X., et al. (2022). DeepST: identifying spatial domains in spatial transcriptomics by deep learning. *Nucleic Acids Research*, 50(22):e131–e131.

Xu et al., 2024. Xu, H., Fu, H., et al. (2024). SEDR: Unsupervised spatially embedded deep representation of spatial transcriptomics. *Genome Medicine*, 16(1):1–15.

Zhou et al., 2023. Zhou, X., Dong, K., et al. (2023). Integrating spatial transcriptomics data across different conditions, technologies and developmental stages. *Nature Computational Science*, 3(10):894–906.
